# Antagonistic roles of *Drosophila* Tctp and Brahma in chromatin remodelling and stabilizing repeated sequences

**DOI:** 10.1038/ncomms12988

**Published:** 2016-09-30

**Authors:** Sung-Tae Hong, Kwang-Wook Choi

**Affiliations:** 1 Department of Biological Sciences, Korea Advanced Institute of Science and Technology, 291 Daehak-ro, Yuseong-gu, Daejeon 305-701, Korea

## Abstract

Genome stability is essential for all organisms. Translationally controlled tumour protein (TCTP) is a conserved protein associated with cancers. TCTP is involved in multiple intracellular functions, but its role in transcription and genome stability is poorly understood. Here, we demonstrate new functions of *Drosophila* TCTP (Tctp) in transcription and the stability of repeated sequences (rDNA and pericentromeric heterochromatin). Tctp binds Brahma (Brm) chromatin remodeler to negatively modulate its activity. *Tctp* mutants show abnormally high levels of transcription in a large set of genes and transposons. These defects are ameliorated by *brm* mutations. Furthermore, Tctp promotes the stability of repeated sequences by opposing the Brm function. Additional regulation of pericentromeric heterochromatin by Tctp is mediated by *su(var)3-9* transcriptional regulation. Altogether, Tctp regulates transcription and the stability of repeated sequences by antagonizing excess Brm activity. This study provides insights into broader nuclear TCTP functions for the maintenance of genome stability.

## Abstract (web summary)

Genome stability is important for normal cellular function. Here, Hong and Choi show that translationally controlled tumour protein (TCTP) in *Drosophila* regulates pericentromeric chromatin remodelling and transcription via negatively regulating a chromatin remodeler Brahma.

## Graphical abstract

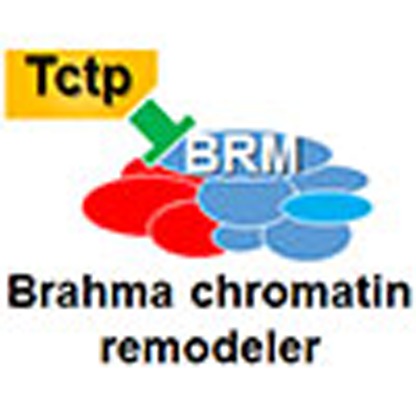

Genome stability is critical for development and survival of all organisms. The DNA of eukaryotic genome is packaged into nucleosomes. Continuous nucleosome arrays form more complex chromatin fibers, resulting in chromosomes^1^ composed of two types of domains: euchromatin and heterochromatin. In general, euchromatin is gene-rich, accessible to DNA binding factors and transcriptionally active, while heterochromatin is gene-poor^2^,^3^ and transcriptionally less active^1^. However, these two domains are distinguished further by other properties, including DNA sequence composition and gene density^1^,^3^.

In fission yeast, flies and mammals, pericentromeric heterochromatin is important for the regulation of sister-chromatid cohesion and centromere stability. Silencing histone marks such as H3K9me2/3, HP1a, and SU(VAR)3-9 are abundant in heterochromatin, and critical for DNA repair in highly repeated sequences^4^,^5^, homologous recombination between repetitive DNA sequences^6^,^7^ and suppression of transposons^8^. Interestingly, silencing histone marks are also required for the stability and repair of rDNA sequences, which are proximal to pericentromeric heterochromatin and partially silenced in *Drosophila*^2^,^3^,^9^. Di/tri-methylation of histone H3K9 in heterochromatic genes and repeats is catalysed by histone SU(VAR)3-9 methyltransferase (HMT) and HP1a, which is crucial for genome stability by maintaining heterochromatin structures^6^.

Besides histones, DNA packaging requires histone chaperones, histone-modifying enzymes and chromatin remodelling complexes (remodelers). Of these, chromatin remodelers are major contributors to the dynamic nature of chromatin because they use the energy from ATP-hydrolysis to organize nucleosomes^10^,^11^. These functions of remodeler complexes allow diverse proteins, such as RNA/DNA polymerases, transcription factors and DNA repair proteins, to access to the DNA and modulate transcription, DNA replication and DNA repairs. Four classes of ATPase remodelers have been identified (SWI/SNF, ISWI, CHD and INO80) according to their unique domains flanking the ATPase region: HSA and Bromo domains for SWI/SNF, SANT and SLIDE domains for ISWI, two tandemly arranged chromo domains for CHD, and HSA for INO80. In flies and mammals, mutants in these remodeler genes show various defects in development, transcription, chromatin structure formation, DNA damage repair, fertility^11^ or cell proliferation^12^,^13^.

The *Drosophila* SWI/SNF remodeler called the Brahma (Brm) complex consists of at least 11 subunits, including 7 core subunits and 4 accessories^14^,^15^. A catalytic ATPase subunit is encoded by brahma (*brm*) gene, which is essential for global transcription by RNA polymerase-II (Pol-II)^16^, inhibition of heterochromatin spreading^17^ and suppression of homeotic transformation by *Polycomb* gene mutations^11^. There are two distinct Brm complexes in *Drosophila* according to their specific compositions of core and accessary subunits: BAP (Brahma-associated proteins) and PBAP (Polybromo-associated BAP)^18^.

In eukaryotes, translationally controlled tumour protein (TCTP) is a conserved protein involved in cancer and various cell functions in both cytoplasm and nucleus^19^,^20^,^21^,^22^,^23^,^24^,^25^. TCTP interacts with many cytoplasmic binding partners to participate in cell growth, proliferation, tumour invasion and apoptosis^19^,^20^,^26^,^27^. In addition, there is evidence that TCTP functions as a transcription factor^21^,^24^ and a nuclear binding partner for ATM kinase during DNA repair^22^,^25^. In our previous study on the interaction between *Drosophila* TCTP (Tctp) and ATM for DNA repair, we observed that Tctp is widely distributed in interband regions of polytene chromosomes in salivary glands (SG). This prompted us to search for broader nuclear functions of Tctp at the level of chromatin.

Here we show that Tctp binding to Brm antagonizes the Brm complex function to regulate gene transcription, transposon activation, and the stability of repeated sequences (rDNA and pericentromeric heterochromatin). Loss of Tctp causes a reduction of *su(var)3-9* transcription, resulting in reduced H3K9me2/3 methylation. Our data suggest that Tctp regulates pericentromeric heterochromatin stability through histone modification and chromatin remodelling. This study provides new insights into the function of TCTP in genome stability and cancer.

## Results

### Tctp directly interacts and colocalizes with Brm

**Figure 1:**
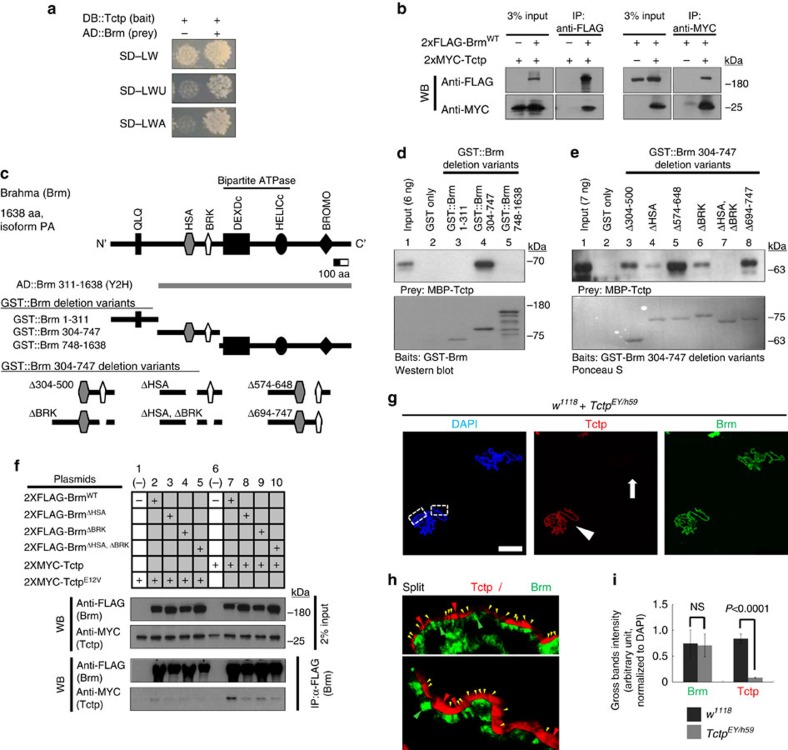
Tctp physically interacts and colocalizes with Brm. (**a**) Y2H (yeast two-hybrid) assay. A functional Gal4 formed by direct interaction between Tctp bait and Brm prey activates the expression of Gal4-responsive selection markers (*URA3* and *ADE2*), allowing yeast transformants to grow on the selection media SD-LWU (-uracil) or SD-LWA (–adenine). (**b**) Reciprocal co-IP. Brm (2xFLAG-Brm^WT^) is co-immunoprecipitated with Tctp (2xMYC-Tctp) in S2 cell and *vice versa*. (**c**–**e**) *in vitro* GST-Pull down assays. (**c**) Schematic domain structure of Brm and a list of mutated Brm polypeptide fragments used in pull-down assays. The full-length Brm protein has 6 conserved domains. (**d**) Direct Tctp binding to the middle part of Brm protein (304-747 amino-acid (aa) region, lane 4). (**e**) Both HSA and BRK domains of Brm are required for direct binding to Tctp (lane 4, 6, 7). (**f**) co-IP in S2 cell expressing 2xMYC-Tctp and 2xFLAG-full-length Brm with deletion of either HSA or BRK, or both domains. Deletion of either HSA or BRK domain of Brm reduces its interaction with Tctp (lanes 8, 9). Contrary to *in vitro* pull down assay, Brm^ΔHSA,ΔBRK^ shows a weak interaction with Tctp (lane 10). Tctp^E12V^ (E12V substitution) substantially interferes with the binding between Tctp and Brm (Lanes 2-5). (**g**–**j**) Tctp colocalizes with Brm in SG polytene chromosomes. (**g**) Squashed polytene chromosomes from the mixed SG tissues of wild-type and *Tctp* mutant larvae, stained with DAPI (blue), Tctp (red), and Brm antibodies (green). Scale bar, 50 μm. Contrary to wild-type chromosomes (red, arrowhead), *Tctp*^*EY/h59*^mutant chromosomes show reduced staining with anti-Tctp antibody (arrow). (**h**) Enlarged split images of the two boxed regions of wild-type chromosomes shown in **g**. Most Tctp signals are colocalized with Brm (yellow arrowheads), but some areas show preferentially either Tctp (red arrowheads) or Brm (green arrowheads). (**i**) The quantified gross bands intensities of Tctp and Brm staining normalized to DAPI (*n*=10 SGs, mean±s.d.). NS, not significant (*P*=0.8).

Tctp is detected in the cytoplasm as well as in the nucleus (Supplementary Fig. 1a) where it is broadly distributed in the interband regions of polytene chromosomes from SG cells^22^. Since the interband regions are transcriptionally active with less-condensed euchromatic conformation^1^, it is possible that Tctp may play a role in the regulation of chromatin structure and transcription. Thus, we used yeast two-hybrid screen to identify novel Tctp-interacting partners and found a Brm polypeptide binding to Tctp (Supplementary Fig. 1b; Fig. 1a,c). Brm is the catalytic ATPase subunit of the Brm chromatin remodeler involved in regulation of pericentromeric heterochromatin boundaries by antagonizing spreading of silencing and in transcriptional gene regulation^10^,^11^,^17^. The physical interaction between Tctp and Brm was reciprocally confirmed by co-immunoprecipitation (co-IP) using nuclear extracts from S2 cells transfected with FLAG-tagged full-length wild-type Brm (Brm^WT^) and MYC-tagged Tctp (Fig. 1b).

*In vitro* pull-down assay using MBP-Tctp and GST-Brm deletion variants (Fig. 1c) revealed that Tctp directly binds to a 304–747 aa region of Brm (Fig. 1d) containing both HSA and BRK domains. Pull-down assays between MBP-Tctp and GST-Brm 304–747 deletion variants indicated that deletion of either HSA or BRK domain strongly reduced the binding between Brm and Tctp, while deletion of both domains abolished the binding of these two proteins. Thus, HSA and BRK domains are partially redundant, and loss of both domains critically impairs the binding between Brm and Tctp *in vitro* (Fig. 1e, Lane 4, 6 and 7) compared with other parts of 304–747 aa region of Brm (Fig. 1e, Lane 3, 5 and 8). Co-IP using S2 cells transfected with 2xMYC-Tctp and 2xFLAG-Brm variants also showed that deletion of either HSA or BRK domain significantly impaired the physical interaction between Tctp and Brm (Fig. 1f, Lane 7–10). Interestingly, however, Brm deleted in both HSA/BRK domains still showed a weak binding to Tctp (Fig. 1f, lane 10). Thus, although HSA and BRK domains are important for proper binding of these two proteins, a weak interaction may still be possible through another region of Brm in culture cells. Glutamate at the position 12 in the N-terminal region of Tctp is necessary for Tctp functions^22^,^26^. The interaction between Tctp and Brm was considerably reduced by the E12 substitution (E12 to V) (Tctp^E12V^), indicating a role of E12 for binding between Tctp and Brm (Fig. 1f, Lane 2–5).

Consistent with these biochemical data, we also observed *in vivo* colocalization between Tctp and Brm in polytene chromosomes (Fig. 1g,h; Supplementary Fig. 2a). To check the effects of *Tctp* mutation, chromosomes were prepared from a mixed SG samples of wild-type and *Tctp*^*EY*^*/Tctp*^*h59*^ trans-heterozygote (hereafter *Tctp*^*EY/h59*^). This trans-heterozygote provides a strong hypomorph condition^22^ that can be identified by reduced Tctp expression. In Tctp-positive wild-type chromosomes, most Tctp colocalized with Brm within the interband regions (Fig. 1g, arrowhead; Fig. 1h, yellow arrowheads), although some sites showed preferentially either Tctp or Brm (Fig. 1h, red arrowheads for Tctp and green for Brm). In *Tctp*^*EY/h59*^ chromosomes, Tctp level was strongly reduced (Fig. 1g, arrow), but Brm seemed to be intact at the gross level (Fig. 1i), although *Tctp*^*EY/h59*^ chromosomes showed minor variations in the Brm banding intensity (Supplementary Fig. 2a, asterisks). Tctp knockdown by *ptc-Gal4* (*ptc>Tctp-i*) also did not cause gross changes in the Brm level (Supplementary Fig. 2c, white arrow). Similarly, Tctp levels were not changed significantly in polytene chromosomes with Brm silencing (*ptc>brm-i*) or dominant-negative Brm^28^ expression (*ptc>Brm*^*DN*^) (Supplementary Fig. 2d–e). Additional tests with RNA interference (RNAi), mutant clones and overexpression conditions indicated that reduction or overexpression of Tctp does not alter the gross level of Brm and *vice versa* (Supplementary Fig. 2f–l). Taken together, our data indicate that Tctp and Brm directly bind to each other, and colocalize in most interband regions of chromosomes.

### Tctp negatively modulates Brm ATPase activity

As shown above, Tctp loss does not greatly affect the level of Brm and *vice versa* in two different tissues. It led us to postulate that Tctp may interact with Brm to regulate the Brm activity rather than its recruitment. Previously, it has been shown that an HSA domain in yeast Sth1 or human Brg-1 ATPase subunit acts as the primary binding platform for nuclear actin-related proteins (ARPs) and β-actin (Act) and this interaction is required to facilitate maximum ATPase activity of the chromatin remodelers^29^,^30^. Since Tctp binds to the HSA domain of Brm and both *Drosophila* ARP4 and Act (ARP4/Act) also bind to the same domain (Supplementary Fig. 3), we examined whether Tctp binding to the HSA domain might promote or interfere with ARP4/Act binding to Brm for regulating Brm ATPase activity.

**Figure 2:**
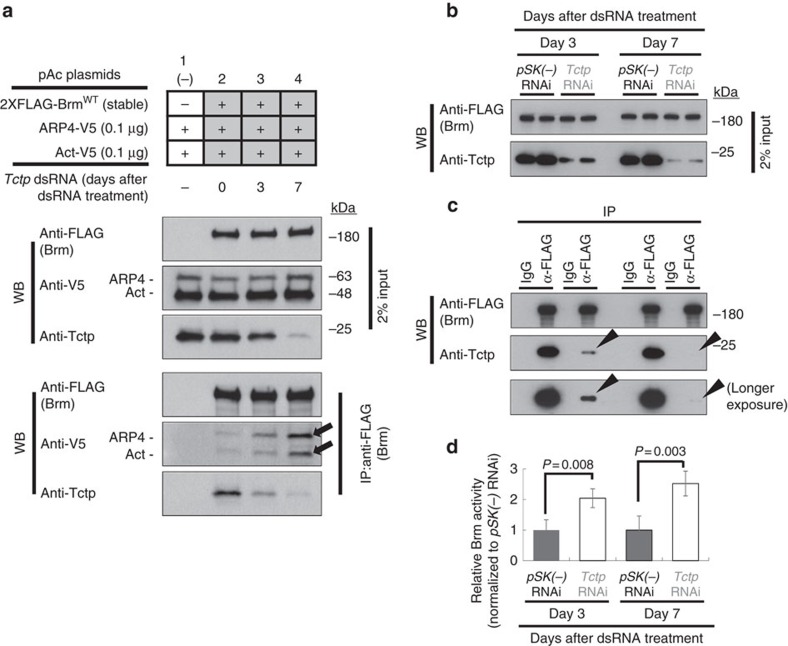
Tctp binding to Brm negatively modulates Brm ATPase activity. (**a**) Enhanced ARP4/Act binding to Brm by Tctp reduction. V5-tagged ARP4 and Act were transiently expressed in S2 cells, stably transfected to produce 2XFLAG-Brm^WT^. Tctp-knockdown by RNA interference (RNAi) using double-stranded RNA (dsRNA) was treated twice at day 0 and 3 to maximize RNAi effect. Immunoprecipitation was performed using FLAG antibody. ARP4/Act binding to Brm was increased by Tctp reduction (arrows). (**b**–**d**) *in vitro* ATPase activity assay in Tctp-knockdowned S2 cells. S2 cells were stably transfected to express 2xFLAG-Brm^WT^. (**b**) dsRNA was treated twice at day 0 and day3. (**c**–**d**) Brm immunoprecipitants from nuclear extracts with low levels of Tctp (arrowheads) were monitored for ATPase assay, relative to control precipitant (*pSK(−)* RNAi). (**d**) Brm ATPase activity was increased up to about 2.5-fold by Tctp-knockdown, compared with *pSK(−)* RNAi control. ATPase activity was normalized to that of *pSK(−)* control. *n*=3 tests (mean±s.d.).

First, we performed co-IP in Tctp-knockdowned S2 cells coexpressing V5-ARP4, V5-act, and FLAG-Brm^WT^ to observe the change in the binding affinity between Brm and ARP4/Act (Fig. 2a). Interestingly, ARP4/Act binding to Brm was increased by Tctp-knockdown (Fig. 2a, arrows). This inverse relationship suggests that Tctp may negatively regulate Brm ATPase activity by interfering with ARP/Act binding to Brm. To attest this postulation, we assayed ATPase activity of Brm using immunoprecipitations in Tctp-knockdowned S2 cell nuclear extracts (Fig. 2b–d). Brm ATPase activity was measured at day 3 and 7 after dsRNA treatment. *In vitro* Brm ATPase activity was determined with a colorimetric Malachite green inorganic phosphate assay^31^,^32^,^33^. In this assay, immunoprecipitants from Tctp-knockdowned S2 cell extract (Fig. 2c) showed about 2.5-fold higher Brm ATPase activity than *pSK(−)* dsRNAi control (Fig. 2d). This result suggests that Tctp binding to Brm negatively modulates Brm ATPase activity by competing with ARP4/Act for binding to the HSA domain.

### *Tctp* shows antagonistic genetic interaction with *brm*

**Figure 3:**
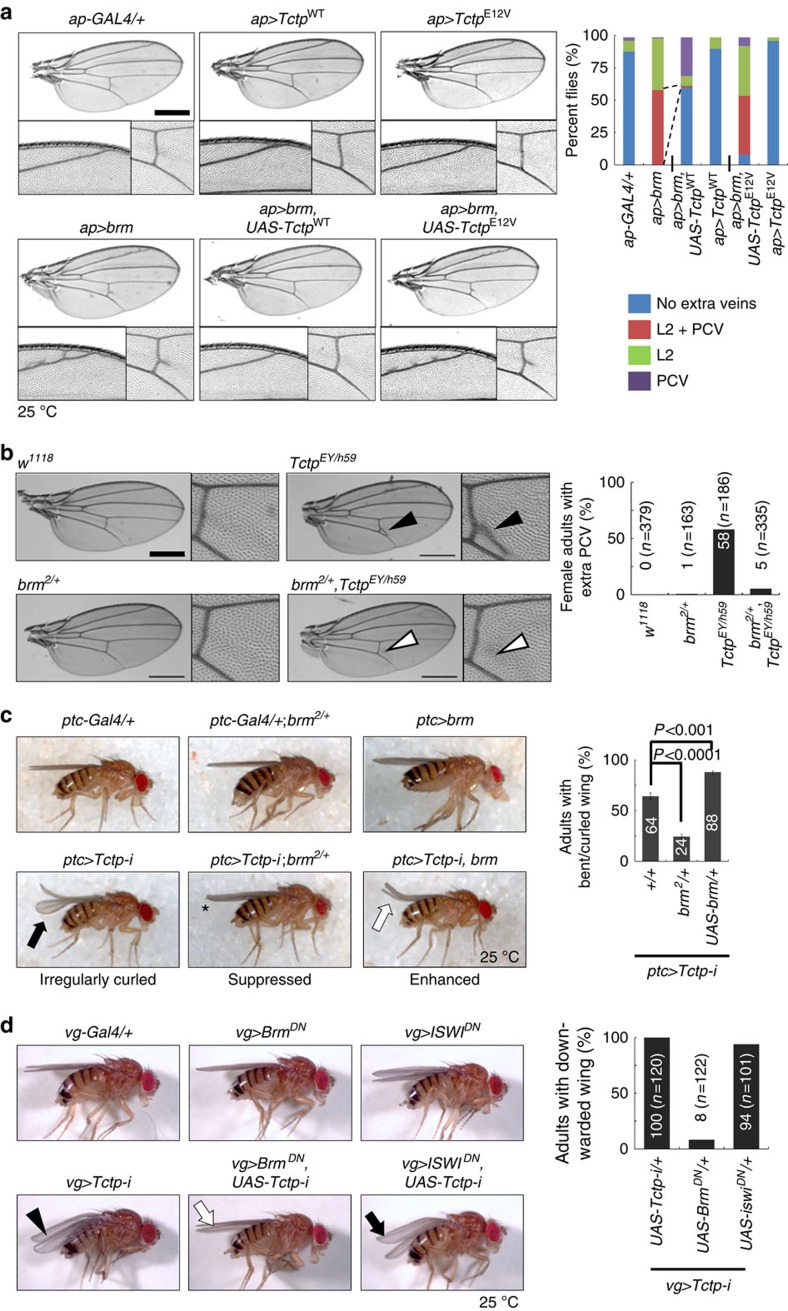
*Tctp* is genetically antagonistic to *brm.* (**a**) Genetic interaction of overexpressed Brm and Tctp. (Top) *ap-Gal4/+*, *ap>Tctp* and *ap>Tctp*^*E12V*^ do not affect wing veins. Below each wing, regions of L2 vein and posterior crossvein (PCV) are shown at a higher magnification. (Bottom) *ap>brm* causes ectopic veins near the L2 vein and the posterior crossvein. Co-expression of Tctp and Brm (*ap>brm*, *Tctp*) considerably suppresses the Brm overexpression phenotype. Overexpression of mutated Tctp^E12V^ fails to suppress the Brm phenotype. (Histogram) Quantification of genetic interactions in percent flies. L2: extra veins in L2 vein. PCV: extra veins in the posterior crossvein. L2+PCV: extra veins in both. *n*=260-330 flies. Scale bar, 500 μm. (**b**) Genetic interaction between *brm* and *Tctp* mutations. Extra PCV (black arrowhead) phenotype in adult *Tctp*^*EY/h59*^ transheterozygotes is suppressed by *brm*^*2*^*/+* mutation (white arrowhead). (Histogram) Quantification of extra PCV phenotype in female adult flies. Scale bar, 500 μm. n=number of flies. (**c**) Antagonistic genetic interaction in wing shape. (Top) *ptc-Gal4*/+, *ptc-Gal4*/+; *brm*^*2*^/+ and *ptc*>*brm* show normal straight wings. (Bottom) *ptc>Tctp-i* flies show partial curled-up wings (black arrow). This phenotype is suppressed (asterisk) or enhanced (white arrow, curled-up wing) by *brm* reduction or overexpression, respectively. (Histogram) Quantification of curled wing phenotype in adult flies (mean±s.d.). *n*=163–237 flies. (**d**) Genetic interaction between *Tctp*-knockdown and dominant-negative Brm with catalytically inactive ATPase (*Brm*^*DN*^). (Top) *vg-Gal4*/+, *vg>brm*^*DN*^, and *vg>ISWI*^*DN*^ show normal straight wings. (Bottom) Downward-kinked adult wing phenotype of *vg>Tctp-i* flies (black arrowhead) was suppressed by coexpressing Brm^DN^ (white arrow), but not by ISWI^DN^ (black arrow). ISWI is an ATPase subunit for other chromatin remodelers such as ACF, CHRAC, and NURF. (Histogram) Quantification of downward-kinked wing phenotype in adult flies. n=number of flies.

Biochemical data shown above indicate a negative relationship between Tctp and Brm. To determine whether Tctp and Brm are antagonistically related *in vivo*, we examined their genetic interactions in the wing. We took an advantage of the observation that Brm participates in wing vein formation by regulating EGFR signalling^34^,^35^. Brm overexpression by *ap-Gal4* (*ap>brm*) in wing discs produced ectopic veins in both L2 and the posterior crossvein regions in about 60% of flies examined (Fig. 3a). This phenotype was considerably suppressed by co-expression of Tctp, but not by Tctp^E12V^ (Fig. 3a). Interestingly, adult *Tctp*^*EY/h59*^ females also showed extra posterior crossvein phenotype (58% flies; Fig. 3b, black arrowheads), which was seen in only about 5% of adult females when *brm*^2^ null heterozygote (*brm*^*2*^*/+*) was introduced (Fig. 3b, white arrowheads). This strong suppression implies that Brm activity increases in *Tctp* mutants, causing ectopic veins in the wing. These genetic interactions suggest that the physical interaction between Tctp and Brm (Figs 1 and 2) is physiologically relevant.

In addition, we confirmed the antagonistic relation between *Tctp* and *brm* using the phenotype of *Tctp*-RNAi induced by *ptc-Gal4*. Adult wings of *ptc>Tctp-i* showed a weak curled-up phenotype (mildly curled or curling of only one wing; Fig. 3c, black arrow). The curled wing of *ptc>Tctp-i* was significantly suppressed or enhanced by Brm reduction or overexpression, respectively (Fig. 3c, asterisk and white arrow), providing additional evidence for an enhanced Brm activity in the *Tctp* mutant background.

The curled wing phenotype of *ptc>Tctp-i* flies was also modified by altered levels of positive modulators of Brm such as ARP4 (encoded by *bap55* gene) and Act. Penetration of the weak curled-up wing phenotype in *ptc>Tctp-i* was considerably reduced to about 50% by heterozygous *bap55*^*EY15967*^ or *act*^*cg0010*^ mutation. In contrast, overexpression of ARP4 or Act enhanced the curled-up wing phenotype marginally or considerably, respectively (Supplementary Fig. 4a). Hence, Tctp is antagonistically related to Brm and its associated regulators in development.

To confirm the increased activation of the Brm function in *Tctp* mutants, we tested genetic interaction by coexpressing *Tctp-i* and Brm^DN^, a dominant-negative form deficient in ATPase activity due to K804R substitution in the ATP-binding site^28^. We used a *vg-Gal4* driver to induce Brm^DN^ expression in the dorsoventral boundary of wing disc (Fig. 3d). The downward-kinked adult wing phenotype of *vg>Tctp-i* flies was suppressed by coexpressing Brm^DN^, indicating that *Tctp* depletion causes activation of Brm function. This genetic interaction between *Tctp* silencing and Brm^DN^ is specific because a dominant negative form of another class of chromatin remodeler ATPase, ISWI (ISWI^DN^, K159R substitution in the ATP-binding site)^36^ showed no genetic interaction with *vg>Tctp-i* flies. Remarkably, high lethality in *ptc>brm*^*DN*^ during mid-and late pupal stages (Supplementary Fig. 4b, black arrows) was considerably suppressed or enhanced by Tctp-knockdown or overexpression, respectively (Supplementary Fig. 4b, blue and red arrows). Collectively, these results indicate that *Tctp* is antagonistic to *brm* function *in vivo*, in accordance with the biochemical evidence for an inhibitory modulation of Brm ATPase activity by Tctp (Fig. 2).

### Aberrant regulation of transcription in *Tctp* mutants

In eukaryotes, Brm remodeler complexes are involved in transcriptional regulation of various target genes during development^11^,^14^,^16^,^18^,^35^,^37^,^38^,^39^,^40^. Especially, in *Drosophila*, PBAP complex physically interacts with the transcription initiation factor IID complex to make a single supercomplex in the promoter region for transcription start process^15^,^41^. In addition, Brm complex with a mutant form of inhibitory SNR1 subunit increases RNA polymerase II (Pol-II) elongation activity in *Eig71E* gene locus 37,40.

**Figure 4:**
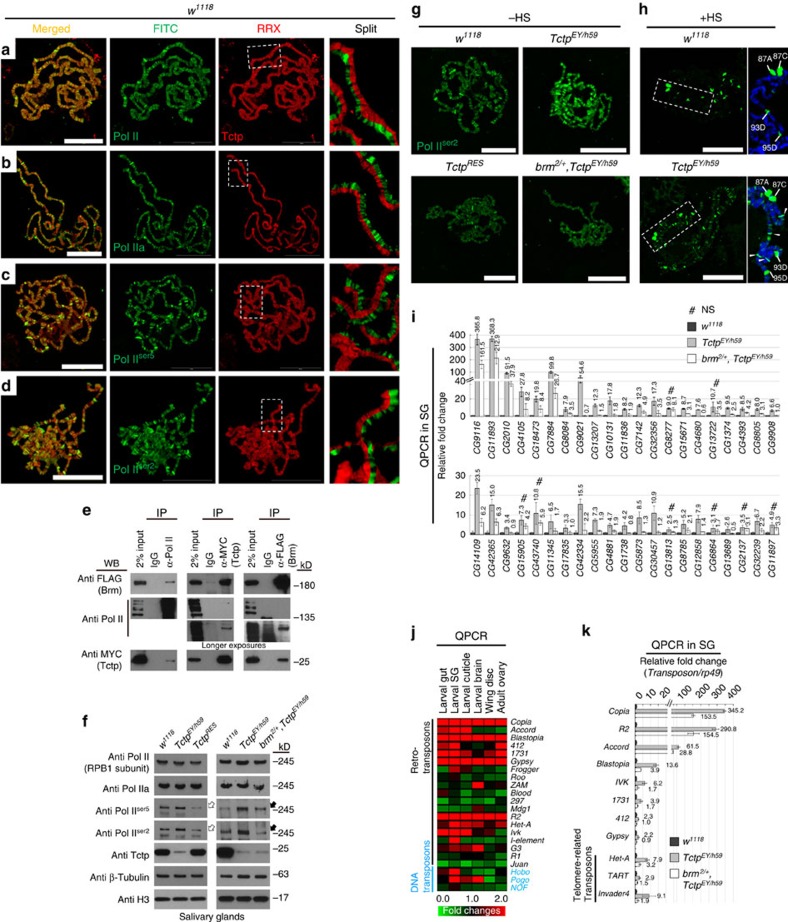
Enhanced expression of a large set of genes and transposons in *Tctp* mutants. (**a**–**d**) Tctp colocalizes with Pol-II in the interbands of squashed SG chromosome stained for Tctp and different CTD forms of the largest Pol-II subunit. (**a**) Staining with anti-Pol-II for non-phospho and phosphorylated CTD in all transcription steps. (**b**) anti-Pol-IIa for non-phosphorylated CTD in the promoter-recruited step. (**c**) anti-Pol-II^Ser5^ for Ser-5-phosphorylated CTD in transcription initiation. (**d**) anti-Pol-II^Ser2^ for Ser-2-phosphorylated CTD in transcription elongation. Boxed areas are enlarged as split images. Scale bars, 50 μm. (**e**) Co-IP using nuclear S2 cell extract transfected with both 2xFLAG-Brm and 2xMYC-Tctp. Tctp forms a large protein complex with Brm and Pol-II. These interactions are resistant to benzonase treatment suggesting direct interaction. The interaction between Tctp and Pol-II or Brm and Pol-II is relatively weak (longer exposures). (**f**) Effects of Tctp on transcription initiation and elongation in SG. Increased Pol-II^Ser5^ and Pol-II^Ser2^ levels in *Tctp*^*EY/h59*^ mutants (white arrows), which are restored by genomic *Tctp*^*Ge30-2*^ in mutants (*Tctp*^*RES*^) or by heterozygous *brm*^2^ mutation (black arrows). Note that the amount of Tctp in *brm*^*2/+*^, *Tctp*^*EY/h59*^ is not restored. β-tubulin and Histone 3 (H3) are loading controls. (**g**,**h**) Chromosome localization of elongating Pol-II. (**g**) Pol-II^Ser2^ localization before heat-shock (-HS). (Top) Increased transcription elongation levels in *Tctp*^*EY/h59*^, compared with *w*^*1118*^ control. (Bottom) Genomic *Tctp*^*Ge30-2*^or *brm*^*2/+*^ rescues the high level of Pol-II^Ser2^ in *Tctp*^*EY/h59*^. (**h**) Elongating Pol-II localization after heat-shock (+HS). (Top) Pol-II^Ser2^ in wild-type exclusively localizes to heat-chock loci after HS. Boxed heat-shock loci between 87 A and 95D are enlarged in the right. (Bottom) Substantial levels of Pol-II^Ser2^ in *Tctp* mutants still remain at many euchromatic sites (white arrowheads). Scale bars, 50 μm. (**i**) QPCR. 34 of 42 genes with high expression levels in *Tctp* mutants were suppressed by *brm*^*2*^/+ mutation. (**j**,**k**) QPCR of transposon expression in *Tctp* mutants. (**j**) Retrotransposons like *Copia*, *Blastopia*, *Gypsy*, and *R2* are highly expressed in various tissues from *Tctp*^*EY/h59*^. (**k**) Additional *brm* mutation in *Tctp*^*EY/h59*^ (*brm*^*2/+*^, *Tctp*^*EY/h59*^) suppresses the high level expression of transposons in SG (mean±s.d.). *Het-A*, *TART*, and *Invader4* are located in the telomere region.

Direct interaction and antagonistic relation between Tctp and Brm led us to ask whether Tctp functions together with Brm to regulate transcription processes by changing the chromatin structure. First, we found that Tctp colocalizes with Pol-II in interband regions of polytene chromosomes (Fig. 4a). Interestingly, different Pol-II antibody markers (specific for the phosphorylation patterns of Pol-II C-terminal domain (CTD) during transcription) showed that most Tctp was colocalized with Pol-IIa of the pre-initiation complex at the promoter regions (Fig. 4b). Tctp was also localized with Pol-II^Ser5^ in the early transcription stage of promoter clearance (Fig. 4c) and Pol-II^Ser2^ in the late transcription stage (Fig. 4d). Consistent with these data, Pol-II antibody detecting both phosphorylated and non-phosphorylated CTD immunoprecipitated Pol-II with Brm and Tctp in S2 cell nuclear extract transfected with 2xFLAG-Brm^WT^ and 2xMYC-Tctp (Fig. 4e, left panels). Reciprocally, 2xMYC-Tctp or 2xFLAG-Brm was also immunoprecipitated with Pol-II, although the binding affinity was weak (longer exposures in Fig. 4e). These data suggest that Tctp and the Brm complex may form a large protein complex with a transcription machinery to regulate the early transcription process.

Next, we examined phosphorylation patterns of Pol-II CTD in SG from *Tctp*^*EY/h59*^ to check the effects of Tctp on Pol-II activity during transcription. Pol-II stability and Pol-II binding to promoter regions in *Tctp*^*EY/h59*^ seemed to be intact because the total amounts of Pol-II (RPB1 subunit) and promoter-recruited Pol-II (Pol-IIa) were similar to wild-type control (Fig. 4f), respectively. On the contrary, Pol-II activities in transcription initiation (Pol-II^Ser5^) and elongation (Pol-II^Ser2^) steps were considerably increased in *Tctp*^*EY/h59*^, and the increased Pol-II activities were restored by introducing a wild-type *Tctp* genomic fragment (*Tctp*^*G30e-2*^) (Fig. 4f, white arrows). Notably, *brm*^*2/+*^ mutation markedly suppressed the increased Pol-II activities in *Tctp*^*EY/h59*^ (Fig. 4f, black arrows). These data suggest that Tctp is required to suppress a step of early transcription by negatively modulating Brm functions.

These results were further confirmed by immunostaining of polytene chromosomes in *Tctp*^*EY/h59*^. Pol-II activity in the transcription elongation step was higher in *Tctp*^*EY/h59*^ chromosomes than wild-type control (Fig. 4g, top; Supplementary Fig. 5a–b), while this phenotype was rescued by *Tctp*^*G30e-2*^ or a reduction of the Brm function (+/*brm*^2^) (Fig. 4g, bottom; Supplementary Fig. 5c–d). Heat-shock in larval stages is known to cause dynamic changes in Pol-II activity such as decreased elongating Pol-II in euchromatin interbands and then increase at several heat-shock loci^16^,^42^. Thus, we tested whether the Pol-II activity difference between wild-type and *Tctp*^*EY/h59*^ is still prominent after heat-shock treatment (Fig. 4h; Supplementary Fig. 5e–f). After heat shock, active transcriptional elongation was exclusively restricted to heat-shock loci such as 87A, 93D and 95D in wild-type chromosomes (Fig. 4h, top). *Tctp*^*EY/h59*^ also showed similar dynamic Pol-II activity changes, but a substantial fraction of Pol-II^Ser2^ was retained at a subset of euchromatic sites (Fig. 4h, white arrowheads). Altogether, these results suggest that Tctp acts in a supercomplex containing the Brm complex and a transcriptional complex, and that in this large complex, Tctp may affect early transcription step and subsequent elongation process by negative modulation of Brm ATPase.

Abnormally enhanced Brm and Pol-II activity in *Tctp* mutants suggested that *Tctp* may be required for proper gene transcription. This possibility was tested using mRNA expression profiles in SG cells from wild-type (*w*^*1118*^), *Tctp*^*h59/+*^ and *Tctp*^*EY/h59*^ (Supplementary Fig. 6a–c). A total of 1,765 genes with more than 1.5-fold differences compared with wild-type were used for heat-map analysis. As expected, expression of these genes was increased or decreased in proportion to the functional *Tctp* gene dosage (Supplementary Fig. 6a). In *Tctp*^*EY/h59*^, about 55% of these genes (964/1,765) displayed increased expression levels, while 34% showed decreased levels (601/1,765) compared with the wild-type level. Gene Ontology analysis revealed that diverse functions of 1,315 genes are misregulated in *Tctp*^*EY/h59*^ with over 1.5-fold changes (*P*<0.05) in their gene expression (Supplementary Fig. 6b; Supplementary Table 1). These gene functions were ranged from apoptosis, DNA repair, or neurogenesis to reproduction, although mRNA profiling was performed using SG tissue. Notably, quantitative PCR analysis (QPCR) on a subset of genes (42) with abnormal expression levels in *Tctp*^*EY*/h59^ revealed that the transcriptional levels of most of these genes (34/42) were considerably recovered by *brm*^*2*/+^ mutation (Fig. 4i), suggesting that abnormal transcription in *Tctp* mutants are likely due to an increase in the Brm function.

Interestingly, we found that of 78 transposons arrayed in a DNA chip, 17 showed markedly elevated levels over 1.5-fold in *Tctp*^*EY/h59*^, while 11 were reduced (Supplementary Fig. 6c). Uncontrolled transposon expression is known to cause various genome instability^43^,^44^,^45^,^46^. Notably, retrotransposons like *R2*, *Copia*, and *Accord* located in pericentromeric regions^43^,^47^ were highly expressed over 100-fold higher than wild-type. In addition, elevated expression of telomere-related TEs (*Invader4* and *TART*) might be related to the telomere fusion phenotype found in *Tctp* mutants^22^. These abnormal transposon expressions were not tissue-specific because QPCR in various tissues from *Tctp*^*EY/h59*^ larvae (gut, SG, brain and wing disc) and adults (ovary) (Fig. 4j and Supplementary Fig. 6d) showed similar results, though transposon expression levels were relatively higher in endocycling tissues (gut, cuticle and SG) than mitotic tissues (brain, wing discs and ovary). These ectopic expressions of retrotransposons in *Tctp*^*EY/h59*^ were considerably reduced in *brm*^*2*/+^, *Tctp*^*EY*/h59^ double mutants to about a half level of *Tctp*^*EY/h59*^ or the level comparable to wild-type control (Fig. 4k). These results suggest that Tctp may be required for proper silencing of genes and transposons by negatively modulating Brm activity.

### Defective position-effect variegation in *Tctp* mutants

Retrotransposons are transcriptionally silenced by heterochromatic marks and associated proteins^2^,^3^,^46^. Because highly expressed transposons in *Tctp*^*EY*/h59^ are located in the pericentromeric region, ectopic expression of transposons in *Tctp* mutants may derive from loss of heterochromatin silencing.

**Figure 5:**
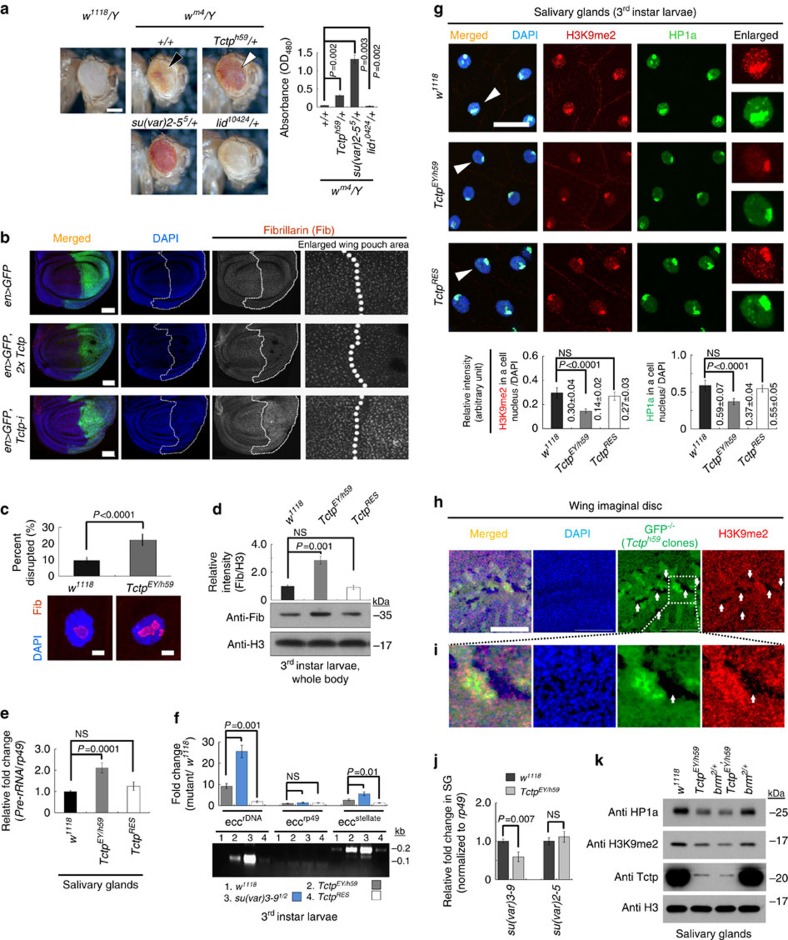
Pericentromeric heterochromatin instability in *Tctp* mutants. (**a**) Suppression of *w*^*m4*^ PEV phenotype by *Tctp*^*h59/+*^(white arrowhead). (Histogram) Quantification of eye pigment (*n*=20 heads, mean±s.d.). Scale bar, 200 μm. (**b**–**f**) Defective gene silencing and pericentromeric heterochromatin instability in *Tctp*^*EY/h59*^ mutants. (**b**) Effects of Tctp on expression of *rDNA* genes. An *en-Gal4* was used to express *UAS*-transgenes only in the posterior compartment of wing disc (dotted GFP-positive region). Fibrillarin (Fib) antibody staining for nucleolus is pseudo-colored in gray. (Top) *en>GFP* discs show similar Fib levels in both anterior and posterior compartments. (Middle) The reduced Fib level and nucleolar size in Tctp-overexpressing cells of the posterior compartment. (Bottom) The increased Fib level and nucleolar size in Tctp-knockdown cells of the posterior compartment. Scale bars, 50 μm. (**c**) Disrupted nucleolar structures in SG cells from *Tctp*^*EY/h59*^. *n*=30 SGs (mean±s.d.). Scale bars, 10 μm. (**d**) Elevated Fib levels in *Tctp*^*EY/h59*^ larvae. *Tctp*^*RES*^ (*Tctp*^*EY/h59*^ with *Tctp*^*Ge30-2*^) shows normal level of Fib. Anti-H3, a loading control for western blotting (mean±s.d.). NS (*P*=0.5). (**e**) Increased *Pre-rRNA* transcript from nucleolar *rDNA* loci in *Tctp*^*EY/h59*^ (QPCR). *Tctp*^*RES*^ rescued *Tctp*^*EY/h59*^ (mean±s.d.). n.s. (*P*=0.5). (**f**) Increased eccDNA formation in the repeated genomic sequences such as *rDNA* and *stellate* genes of *Tctp*^*EY/h59*^. *su(var)3-9*^*1/2*^, a positive control. QPCR was shown as a fold change relative to wild-type (mean±s.d.). A representative gel image is shown. NS (*P*=0.1). (**g**–**k**) Reduced level of H3K9me2 or HP1a heterochromatic silencing mark in *Tctp* mutants. (**g**) (Top) High levels of silencing marks in SG nucleus from wild-type (*w*^*1118*^). (Right-most) The enlarged image of a nucleus marked by an arrowhead. (Middle) Reduced levels of silencing mark in *Tctp*^*EY/h59*^. (Bottom) *Tctp*^*Ge30-2*^rescues *Tctp*^*EY/h59*^ from reduced levels of silencing marks. Scale bar, 50 μm. (Histograms) Quantified silencing mark levels (*n*=10–14 SGs, mean±s.d.). NS (*P*=0.1). (**h**,**i**) Reduced H3K9me2 levels in *Tctp*^*h59*^ null mitotic clones (GFP-negative, arrows) within the developing wing disc. The boxed region is enlarged in **i**. Scale bar, 50 μm. (**j**) QPCR in SG tissues. Reduced *su(var)3-9* transcription in *Tctp*^*EY/h59*^ (mean±s.d.). NS (*P*=0.3). (**k**) Western blot on SG tissues. The levels of both H3K9me2 and HP1a in *Tctp*^*EY/h59*^ are reduced compared with wild-type.

To test this assumption, we checked whether Tctp can affect position-effect variegation (PEV). PEV is a phenotypic variegation caused by random suppression of an euchromatic gene due to its abnormal juxtaposition to heterochromatin 48. We utilized the PEV phenotype of *In(1)w*^*m4*^ (Fig. 5a), in which the *white* (*w*) gene is transposed close to pericentromeric heterochromatin on the X chromosome by a large inversion (Supplementary Fig. 7a)^49^,^50^. Due to PEV, *w*^*+*^ expression is suppressed in *w*^*m4*^*/Y* males, resulting in the formation of unpigmented or weakly pigmented regions in the eye (Fig. 5a, black arrowhead). In control tests, the *w*^*m4*^ PEV phenotype was suppressed by a mutation in the *su(var)2-5* gene encoding HP1a protein, one of the key proteins maintaining the heterochromatin structure. In contrast, this PEV phenotype was enhanced by a mutation in *lid* encoding a histone demethylase (Fig. 5a). We then examined the effects of *Tctp* mutations on PEV. *Tctp* heterozygous null mutation (+/*Tctp*^*h59*^) considerably suppressed the *w*^*m4*^ phenotype, resulting in about 6-fold higher eye pigmentation than *w*^*m4*^ control eye (Fig. 5a, white arrowhead). We also confirmed that *Tctp*^*h59/+*^mutation increased the level of *white* gene transcription by about 2.5-fold in the *w*^*m4*^ line (Supplementary Fig. 7b). These data suggest that silencing of *w*^*m4*^ is compromised by *Tctp* mutation.

Besides *w*^*m4*^ allele, *Tctp* mutation also suppressed PEV phenotypes of two additional lines, *T(2;3)Stubble*^*Variegated*^ (*Sb*^*v*^) and *Tp(3:Y)BL2* (Supplementary Fig. 7c–d). In *Sb*^*v*^, a defined chromatin region near the *Sb* gene is translocated by the centromeric heterochromatin region of the 2nd chromosome, which causes shortening of 1 or 2 scutellar macrochaete bristles by PEV. *Tctp*^*h59*/+^ significantly increased the number of shortened scutellum bristles in *Sb*^*V*^ line (about 1.8-fold compared with +/+ control; Supplementary Fig. 7c). In *Tp(3;Y)BL2/+*, a *HS-lacZ* transgene inserted in the euchromatic region of the third chromosome (65E)^48^,^49^ is transposed to the heterochromatic Y chromosome^9^, leading to a strong suppression of LacZ expression by PEV in the SG. Both +/*Tctp*^*h59*^and +/*Tctp*^*EY09182*^ increased the number of SG cells expressing LacZ in *BL2* line (Supplementary Fig. 7d, about 7-fold increase compared with +/+ control). Both cases of PEV suppression by *Tctp* mutants were fully restored by introducing *Tctp*^*Ge30*-2^ (Supplementary Fig. 7c–d). Altogether, these results strongly suggested that Tctp is required to maintain pericentromeric heterochromatin silencing or boundaries.

### Instability of the repeated sequences in *Tctp* mutants

In eukaryotic genome, silencing histone marks protects repetitive sequences (such as ribosomal RNA genes (*rDNA*)) and pericentromeric repeats by inhibiting recombination between DNAs with similar sequences. In addition, transcriptional silencing of specific genes such as *rDNA* and transposons prevents cell lethality caused by excessive gene/transposon expression^8^,^51^.

Since tandemly-repeated *rRNA* genes are proximal to pericentromeric heterochromatin region and their expression is partially suppressed by silencing histone marks^9^, we addressed whether *rRNA* gene silencing and *rDNA* structure are impaired in *Tctp* mutants. First, we checked Fibrillarin protein level (Fib; an RNA-binding protein for efficient processing of *Pre-rRNA* in nucleolus) in Tctp-knockdowned wing discs from *en>Tctp-i* larvae. These discs showed considerably increased Fib protein levels and nucleolar size in the posterior region (Fig. 5b, bottom) compared with the anterior compartment of the same disc or *en>GFP* control. *Tctp* overexpression in the posterior compartment showed a marginal reduction in the Fib level (Fig. 5b, middle). In SGs, the nucleolar morphology was also disrupted with about 2-fold increase in the Fib-positive areas in *Tctp*^*EY/h59*^ (Fig. 5c), suggesting abnormal repair of double-strand breaks (DSBs) in these sequences^6^. Furthermore, the Fib protein level in the whole body from *Tctp*^*EY/h59*^ was increased about 2.9-fold (Fig. 5d). Consistent with these results, QPCR of *rRNA* transcription in SG showed about 2-fold increase in *Tctp*^*EY/h59*^ (Fig. 5e).

During normal development, tandemly repeated sequences often make extrachromosomal circular DNA (eccDNA) through intramolecular homologous recombination between tandem repeats, causing genome instability. This eccDNA formation is markedly increased when genome is exposed to mutagens causing DSBs or when heterochromatin formation is defective by *su(var)3-9*^*1/2*^ mutation (Fig. 5f)^6^,^51^. Interestingly, *Tctp*^*EY/h59*^ showed an increased eccDNA formation from the tandem-repeats such as *rDNA* (9.5-fold) and *stellate* (encoding casein kinase beta subunit; 2.9-fold) genes (Fig. 5f). The *stellate* locus is located in the pericentromeric heterochromatin of chromosome X^9^. Reduced gene silencing and increased eccDNA in *Tctp*^*EY/h59*^ were restored to the wild-type level by introducing *Tctp*^*Ge30-2*^ (*Tctp*^*RES*^ in Fig. 5d–f). These results suggest that Tctp is required to maintain the stability of *rDNA* loci and pericentromeric repeated sequences.

### Reduced heterochromatic silencing components in *Tctp* mutants

H3K9me2/3 histone modification catalysed by SU(VAR)3-9 HMT is an essential step for the recruitment of HP1a, leading to heterochromatin formation or gene silencing in *Drosophila*. Thus, H3K9 methylation and HP1a are convenient markers for silencing associated with heterochromatin structure^3^,^6^,^8^,^46^.

Since mutations in *Tctp* and *su(var)3-9* result in similar induction of eccDNAs in *rDNA* and heterochromatic sequences^6^, we assessed whether *su(var)3-9* and other silencing components are affected in *Tctp* mutants. Indeed, larval SG tissues from *Tctp*^*EY/h59*^ or *Sgs3>Tctp-i* showed considerably reduced levels of H3K9me2 and HP1a (Fig. 5g; Supplementary Fig. 7g). In cell nucleus from *Tctp*^*EY/h59*^, H3K9me2 level was reduced to about 47% of wild-type (Fig. 5g). Likewise, polytene chromosomes from SGs of *Tctp*^*EY/h59*^ showed about 63% reduced H3K9me2 levels in the chromocenter (Supplementary Fig. 7e–f). Consistent with these results, both the mitotic *Tctp*^*h59*^ null clonal cells of wing disc and the posterior compartment of *en>Tctp-i* wing discs showed strong reduction in the levels of H3K9me2 (Fig. 5h,i) and H3K9me3 (Supplementary Fig. 7i, arrowhead), respectively. In addition, HP1a levels were reduced in cell nuclei and polytene chromosomes from *Tctp*^*EY/h59*^ (Fig. 5g; Supplementary Fig. 7e–f). The reduction of silencing marks in *Tctp* mutants was restored by introducing *Tctp*^*Ge30-2*^ (Fig. 5g and Supplementary Fig. 7e, *Tctp*^*RES*^).

The reduction of silencing marks in *Tctp* mutants appears to result from the decreased amounts of SU(VAR)3-9 HMT protein, rather than the decreased enzyme activity for three reasons. Firstly, *su(var)3-9* transcription was reduced in *Tctp*^*EY/h59*^ to about a half of the wild-type level (Fig. 5j). Secondly, chromatin IP assays revealed that Pol-II accumulation to *su(var)3-9* locus was reduced in Tctp-knockdowned S2 cells to about 47% of *pSK(-)* RNAi control (Supplementary Fig. 8d). Finally, direct interaction among Tctp, SU(VAR)3-9, and HP1a was not detected in our assay (data not shown). Notably, transcription of *su(var)2-5* gene encoding HP1a in *Tctp*^*EY/h59*^ was intact, although its protein level was decreased (Fig. 5j,k). This suggests that the stability of HP1a protein might be reduced in *Tctp* mutants by the decreased level of SU(VAR)3-9 protein. We also checked whether the effects of *Tctp* mutations on the level of silencing marks are related to an increase in the Brm function. Interestingly, the reduced levels of H3K9me2 and HP1a in *Tctp*^*EY/h59*^ were not restored by *brm*^2^*/+* (Fig. 5k). Furthermore, the level of Brm in the *su(var)3-9* locus was very low and unchanged by Tctp-knockdown (Supplementary Fig. 8f).

Overall, these results suggest that Tctp is required for *su(var)3-9* transcription, thus affecting the levels of H3K9 methylation and HP1a. These are likely to contribute to the formation of eccDNA in *rDNA* and pericentromeric heterochromatin. In this process, *Tctp* mutants are not affected at least by partial reduction of Brm.

### Brm reduction suppresses genome instability in *Tctp* mutant

**Figure 6:**
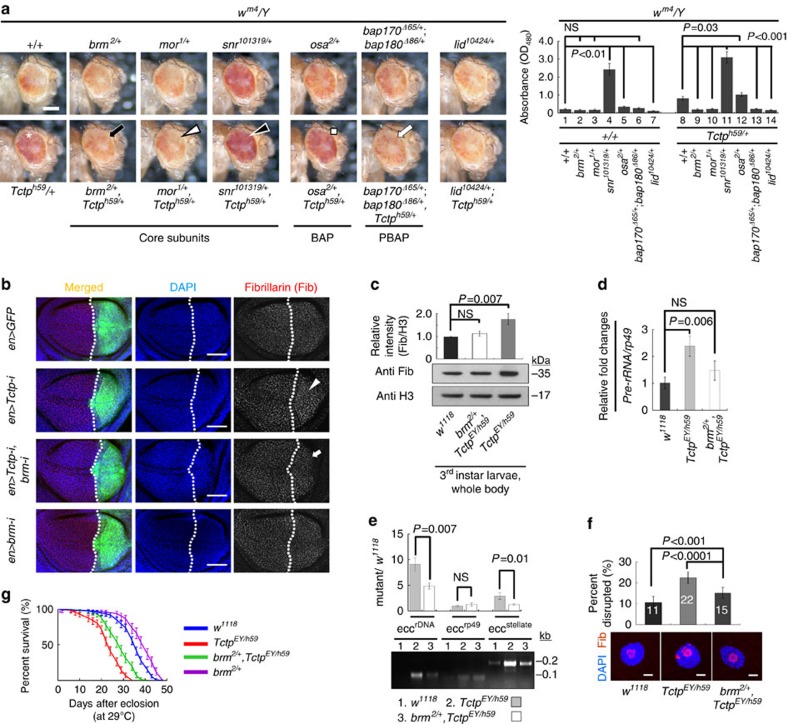
Defective gene silencing and genome stability in *Tctp* mutants are ameliorated by *brm* mutation. (**a**) Genetic interaction between Tctp and Brm complex subunit genes. (Top panels) *w*^*m4*^ eye color PEV phenotype in the wild-type background (+/+) and the effects of mutations in Brm complex genes as indicated. *lid*^*10424*^ is a PEV enhancer control. (Bottom panels) *Tctp*^*h59*^*/+* heterozygous mutation strongly suppresses the *w*^*m4*^ PEV (asterisk in the first panel). The effects of *Tctp*^*h59*^*/+* is significantly suppressed by *brm*^*2/+*^ mutation (black arrow). Likewise, *mor*^*1*^/+ (white arrowhead) and *bap170*^*Δ65*^*/+*;*bap180*^*Δ86*^*/+* double mutations (PBAP components, white arrow) suppress the *Tctp*^*h59*^*/+* effect. In contrast, *snr*^*101319*^*/+* mutation in an inhibitory subunit gene (black arrowhead) or *osa*^*2*^*/+* (BAP component) does not suppress *Tctp*^*h59*^*/+*. Scale bar, 200 μm. (Histogram) Quantification of eye pigment (Mean±s.d. *n*=20 heads). (**b**,**c**) Increased Fib levels in *Tctp* mutants. (**b**) Increased Fib level in the posterior compartment of *en>Tctp-i* flies (arrowhead) is suppressed by Brm-knockdown (arrow). Scale bars, 50 μm. (**c**) Increased Fib level in *Tctp*^*EY/h59*^ whole body. Fib (nucleolus marker) and H3 (loading control) antibodies were used for western blotting or immunostaining. (**d**,**e**) QPCR on *Pre-rRNA* transcription and eccDNA formation. Additional heterozygous *brm*^*2*^ mutation in *Tctp*^*EY*/h59^ ameliorates defective gene silencing in *rDNA* loci from SG cell nucleoli (**d**) and larval eccDNA formation in repetitive sequences (**e**). Normalized DNA levels are shown as fold changes relative to the wild-type level (mean±s.d.). A representative gel image is shown. (**f**) Abnormal nucleolar morphology in *Tctp*^*EY/h59*^ are improved by *brm*^*2*^/+ mutation. Scale bars, 10μm. (**g**) Shortened life span in adult *Tctp*^*EY/h59*^ males (red) relative to wild-type (blue; log-rank test, *P*<0.0001) is ameliorated by reducing Brm level (*brm*^*2/+*^,*Tctp*^*EY/h59*^; green; log-rank test, *P*<0.0001). Adult flies were incubated at 29 °C.

Heterozygote for *brm*^*2*^ mutation can enhance PEV or TPE (telomeric position effect) phenotype by increasing heterochromatin-mediated suppression of *w*^*+*^ expression in adult male^17^,^52^,^53^. Because Tctp and Brm directly interact each other and are biochemically and genetically antagonistic, we addressed whether the suppression of *w*^*m4*^ PEV phenotype in *Tctp*^*h59/+*^ may be restored by *brm*^*2*^ mutation. Indeed, we found that *brm* mutation (*+*/*brm*^*2*^) strongly suppressed the effects of *Tctp* mutation (*Tctp*^*h59*^*/+*) on the *w*^*m4*^ PEV phenotype (Fig. 6a, black arrow and histogram). Consistent with this, core subunit genes of the Brm complex such as *mor* and *snr1* also interacted genetically with *Tctp* in the *w*^*m4*^ background. Heterozygous *mor* mutation (+/*mor*^*1*^), a known *w*^*m4*^-enhancer, reduced eye pigmentation of *Tctp*^*h59/+*^ (Fig. 6a, white arrowhead), while a known *w*^*m4*^-suppressor, heterozygous *snr1* mutation (+/*snr1*^*101319*^), increased eye color (Fig. 6a, black arrowhead). These data suggest that pericentromeric heterochromatin instability in *Tctp* mutants is related to an abnormal activation of the Brm complex function, although the reduced levels of the silencing marks, H3k9me2/3 and HP1a in *Tctp*^*EY/h59*^ were not restored by *brm*^*2*^*/+* mutation.

**Figure 7:**
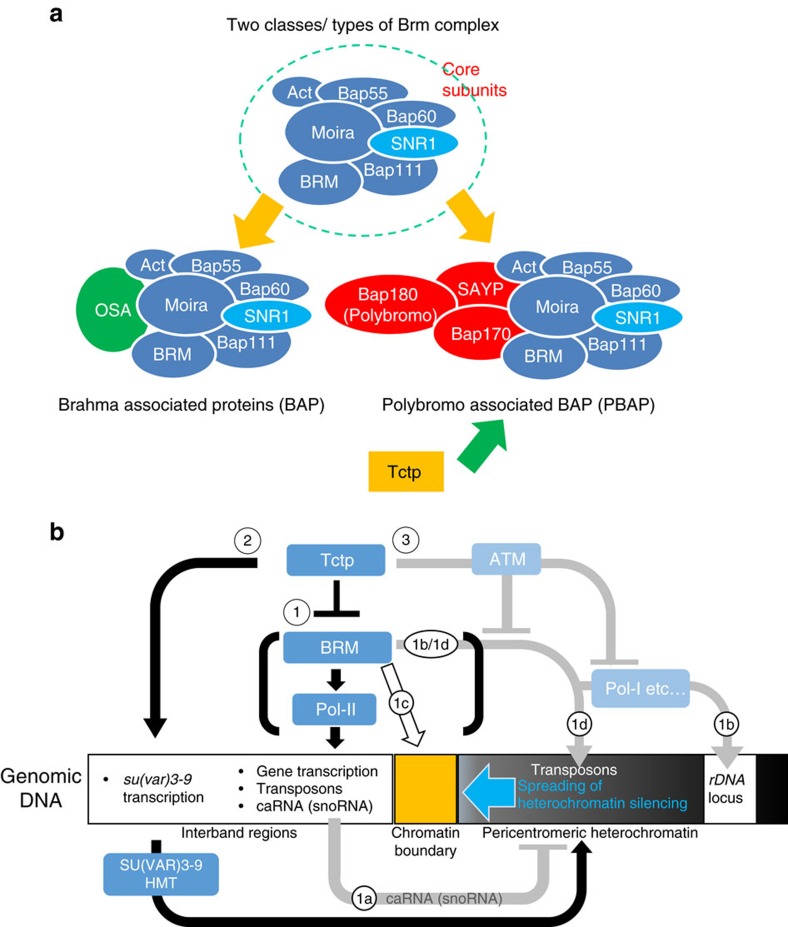
Genetic interaction between Tctp and PBAP and a model for nuclear Tctp function for genome stability. (**a**) The schematic drawing of Brm chromatin remodelling complexes. Brm-containing chromatin remodelers can be divided into two types: BAP and PBAP according to their specific subunits, OSA and Bap160/170/SAYP, respectively. Based on genetic interactions (Fig. 6), Tctp seems to preferentially interact with PBAP than BAP. (**b**) A schematic model of novel nuclear Tctp functions for the stability of repeated sequences (*rDNA* and pericentromeric heterochromatin). Note that *rDNA* locus is not a part of heterochromatin although it is proximal to or surrounded by pericentromeric heterochromatin. Tctp keeps the stability of repeated sequences through three possible mechanisms: (Step 1) Tctp inhibits excess Brm activity, thereby regulating proper transcription level of various genes including transposons and stabilizing pericentromeric heterochromatin. caRNA expression and Pol-I activity regulated by Brm may also affect stability of pericentromeric heterochromatin (Step 1a and 1b, respectively). Brm may regulate the chromatin boundary (Step 1c). It might also be possible that Tctp mutations affect PEV by elevating the Brm activity within the regions of pericentromeric heterochromatin (Step 1d). (Step 2) Tctp positively regulates *su(var)3-9* transcription by direct binding to *su(var)3-9* locus, facilitating H3K9 methylation in pericentromeric regions. (Step 3) Brm or Pol-I may also be suppressed by the interaction between Tctp and ATM. See Discussion for details.

In most eukaryotes including flies, Brm-containing chromatin remodelers are divided into two subclasses according to their specific accessary subunits, BAP (SWI/SNF in yeast and BAF in mammals) and PBAP (RSC in yeast and PBAF in mammals; Fig. 7a). BAP contains OSA, whereas PBAP has Bap170, 180 and SAYP. *Tctp*^*h59/+*^ showed strong genetic interaction with double heterozygous mutations of *bap170/180* (+/*bap170*^*Δ65*^;+/*bap180*^*Δ86*^) in the *w*^*m4*^ background, resulting in suppression of the *Tctp* mutation effects (Fig. 6a, white arrow). In contrast, the *Tctp* mutant effects were not modified by heterozygous *osa* mutation (+/*osa*^*2*^; Fig. 6a, square). Thus, Tctp seems to interact more preferentially with PBAP than BAP to facilitate pericentromeric heterochromatin silencing.

Antagonistic interaction between Tctp and Brm in *w*^*m4*^ PEV phenotype suggested that abnormally enhanced function of Brm in *Tctp* mutants may exacerbate the stability of silenced repeated sequences. We tested this possibility in *brm* and *Tctp* double mutant or RNAi conditions (Fig. 6b–f). As expected, various characteristics of genome instability in *Tctp* mutants, including elevated *rDNA* expression, abnormal nucleolar morphology, and eccDNA formation, were ameliorated by reducing Brm activity in the double mutant condition *(brm*^*2/+*^,*Tctp*^*EY/h59*^ or *en>Tctp-i,brm-i*). Increased Fib level in *en>Tctp-i* or *Tctp*^*EY/h59*^ was considerably suppressed by Brm-knockdown (*UAS-brm-i*) or *brm*^*2*/+^ mutation, respectively (Fig. 6b,c). Consistent with this, increased *Pre-rRNA* expression, eccDNA formation and disrupted nucleolar morphology in *Tctp*^*EY/h59*^ were also suppressed by *brm*^*2*/+^ mutation (Fig. 6d–f).

It has been shown that increased transposon expression causes life span reduction by *de novo* insertional mutations or mRNA toxicity^45^. Similarly, adult *Tctp*^*EY/h59*^ males showed a shortened life span (32.8±1.5-d-old) compared with wild-type (43.8±2.1 days) at 29 °C (Fig. 6g). This phenotype was improved by 5.4 days in the presence of *brm*^*2/+*^ (38.2±1.7 days), suggesting that the reduced life span in *Tctp*^*EY/h59*^ might be due to increased Brm function to some extent.

## Discussion

Heterochromatin silencing has important roles in the maintenance of genome stability. Silencing histone marks prevents transposon jumping and abnormal recombination between repetitive sequences (*rDNA* and pericentromeric heterochromatin), thereby restraining structural lesions in chromosomal DNA. Here we have shown a multitude of evidence that Tctp is required to stabilize pericentromeric heterochromatin in concert with Brm remodeler and to regulate *su(var)3-9* histone gene transcription for producing histone marks for silencing.

Tctp physically interacts with Brm, providing the basis for their genetic interaction. Tctp and Brm proteins also show extensive co-localization in the interband regions of polytene chromosomes and they work antagonistically in the regulation of gene expression. Analysis of chromosomal localizations in *Tctp* or *brm* mutant SGs indicates that Tctp is not critically required for the localization of Brm and *vice versa* at the gross level. This suggests that the physical interaction between Tctp and Brm might be involved in the regulation of their activity rather than their chromosomal localization. In fact, Tctp knockdown results in strong upregulation of the ATPase activity of Brm, suggesting that an important function of Tctp is to inhibit the ATPase activity of Brm. Precise mechanism for the Tctp-mediated inhibition of the Brm ATPase activity is currently unknown. However, we have shown that reduced Tctp facilitates binding of both ARP4 and Act to the HSA domain of Brm. Hence, Tctp seems to antagonize the Brm ATPase activity by competitively interfering with the binding of ARP4 and Act. The biochemical evidence for the Tctp function of inhibiting the Brm activity is strongly supported by antagonistic genetic interactions between *Tctp* and *brm* in multiple organs like eye, wing and SG, and even in the survival rate of adult flies. Hence, Tctp and Brm antagonistically affect gene expression and developmental events as well as adult life of flies. In addition to its antagonistic role with Brm, Tctp is required for the expression of Su(var)3-9 HMT, thus promoting H3K9 methylation and pericentromeric heterochromatin silencing and stability.

On the basis of these findings, we propose a model for the functions of Tctp in the regulation of transcription, heterochromatin silencing, and the stability of repeated sequences (Fig. 7b). In this simplified scheme, Tctp regulates gene transcription and heterochromatic stability. To stabilize repeated sequences, Tctp plays two related functions: a negative regulation of Brm activity and a positive regulation of *su(var)3-9* expression, the step (1) and (2) in Fig. 7b, respectively. In the step 1, *Drosophila* Brm participates in the processes for gene transcription^14^,^16^,^35^,^37^,^39^ and the inhibition of heterochromatin spreading^17^,^52^,^53^. Our data show that both of these Brm functions are negatively regulated by Tctp. For example, Tctp is necessary for suppressing excessive or unwanted transcription of heterochromatic genes like transposons and for preventing eccDNA formation or heterochromatin spreading by suppressing the Brm remodeler activity. The role of Tctp in regulating the transcription of repair genes such as *Ku70/80* and *tefu* might in part account for the instability of repeated sequences observed in the absence of Tctp.

Our data for the role of Tctp in stabilizing the pericentromeric heterochromatin may also be explained by the inhibitory function of Tctp to Brm (step 1, Fig. 7b). Interestingly, transcription of some snoRNA molecules including chromatin-associated RNA (caRNA; CR34558, CR34684, and CR33674) is upregulated in *Tctp* mutants, and these effects are suppressed by *brm* mutation (Supplementary Fig. 11). Hence, Tctp might be involved in inhibiting the Brm function of producing small ncRNAs (non-coding RNAs) such as snoRNA or caRNA. Small ncRNA including snoRNA and small interfering RNA have important roles in the formation of heterochromatin or gene silencing in eukaryotes^54^,^55^. Especially, since caRNA is a mediator for the establishment of open chromatin states in flies and humans^56^, increased Brm function might affect pericentromeric silencing via caRNA (step 1a, Fig. 7b).

It is also worth noting that in mammals, human Brm homologues can interact with nucleolar proteins^57^,^58^,^59^. Thus, uncontrolled Brm access to the nucleolus in *Tctp* mutant cells might affect *rDNA* instability via interacting with Pol-I or other components, as indicated by the step 1b in Fig. 7b. Brm chromatin remodeler can also affect pericentromeric heterochromatin by regulating the chromatin boundary in *Drosophila*^17^,^52^ (step 1c, Fig. 7b). In this process, Brm works together with the GAGA-FACT-HIRA complex for Histone3.3 (H3.3) replacement at the chromatin boundary, thereby inhibiting the spreading of hetrochromatin silencing^17^. However, it has been unknown how Brm function at the boundary is regulated. In this regard, it is noteworthy that *Tctp* mutants show abnormal PEV. This defect is considerably suppressed by reducing the *brm* gene dosage. Our data suggest that Tctp may negatively regulate Brm function at the chromatin boundary. However, since localization of PBAP subunits or ASF1 interacting with Brm is also detected in the regions of pericentromeric heterochromatin^60^,^61^,^62^, we do not exclude the possibility that Tctp mutations may affect PEV by elevating the Brm activity within the regions of pericentromeric heterochromatin (Step 1d).

In addition to the function of Tctp through its interaction with Brm, we also propose an additional mechanism of Tctp in controlling the stability of pericentromeric heterochromatin. In this pathway (step 2, Fig. 7b), Tctp is required for histone modification by regulating *su(var)3-9* expression. Our data show that both levels of *su(var)3-9* HMT transcription and H3K9me2/3 silencing mark are reduced in *Tctp* mutants. Further, ChIP analysis also supports that Tctp may function as a positive transcriptional modulator to mediate Pol-II accumulation on the *su(var)3-9* locus. Because Tctp-dependent regulation of *su(var)3-9* transcription is not modified by *brm* mutation, Tctp appears to regulate the expression of *su(var)3-9* in a Brm-independent manner. Although Tctp can directly bind to *su(var)3-9* locus for positive regulation, the precise role of Tctp for transcription control of *su(var)3-9* is currently unknown. However, because mammalian TCTP regulates transcription of *oct4*/*nanog* genes^21^,^24^, *Drosophila* Tctp might function as a transcription factor or a transcriptional regulator for *su(var)3-9*. It is also possible that like *Drosophila* SNR1 (ref. 40), Tctp might regulate the stability of *su(var)3-9* transcript during transcriptional elongation process.

Our findings show that Tctp contributes to the stability of heterochromatin by its physical interaction with Brm. Previous studies have shown that Tctp directly interacts with ATM-kinase to promote DNA damage repair in *Drosophila* and mammalian cells^22^,^25^. Interestingly, ATM is also known to inhibit local Pol-II and pan-nuclear Pol-I activities in response to DSB^63^,^64^. Thus, it raises a question whether impaired ATM activity resulting from reduced Tctp might be responsible for increased Pol-II and Pol-I activity in *Tctp* mutants. Our tests for this possibility indicated that, in the absence of DSB induction, loss of ATM does not increase Pol-II activity in developing tissues of *Drosophila* and only leads to a weak increase in the Pol-I activity (Supplementary Fig. 9a and Supplementary Discussion for details).

It has been reported that mammalian Brm homologs like hBrm and Brg-1 act together with ATM to repair IR-induced DSBs at γ-H2AX foci^65^. Unlike Tctp, however, *Drosophila* Brm shows little co-localization with γ-H2Av foci, and reduced Brm does not affect the level of γ-H2Av, suggesting that ATM might not work together with Brm for DNA repair in developing organs of *Drosophila* (Supplementary Fig. 10 and Supplementary Discussion). On the other hand, we noted that heterozygous *atm* mutation can suppress *w*^*m4*^ PEV phenotype (Supplementary Fig. 9b, arrow), and this suppression is significantly counteracted by *brm* heterozygous mutation (Supplementary Fig. 9b, black arrowhead). Since this genetic interaction between *atm* and *brm* is similar to that of between *Tctp* and *brm*, it appears that Tctp-ATM complex might negatively regulate Brm function to stabilize heterochromatin boundaries or epigenetic modifications (Fig. 7b, Step 3). However, genetic interaction between Tctp and ATM in *w*^*m4*+^ PEV might be additive rather than synergistic (Supplementary Fig. 9b, white arrowhead). Thus, it is possible that *Drosophila* ATM and TCTP might function in parallel pathways to promote heterochromatin silencing or the establishment of its boundaries by negatively regulating Brm activity. Further studies are necessary to understand the precise relationship between ATM and TCTP. Nonetheless, our data suggest critical roles of Tctp in antagonizing the Brm function for the regulation of gene transcription and genome stability, providing insights into possible similar functions of human TCTP.

## Methods

### Fly stocks and genetics

All crosses were carried out at 25 °C on standard cornmeal media unless otherwise specified. *w*^*1118*^ flies were used as the wild-type. Fly stocks of *Sb*^*V*^ (BL878), *In(1)w*^*m4*^; *su(var)2-5*^*5*^ (BL6234), *su(var)3-9*^*1*^ (108-675), *su(var)3-9*^*2*^ (108-676), *lid*^*10424*^ (BL12367), *Sgs3-Gal4* (BL6870; SG-specific Gal4 driver), *ptc-Gal4* (BL2017), *MS1096-Gal4* (BL8860), *UAS-brm* (BL42229), *UAS-brm-RNAi* (*brm-i*, BL31712), *mor*^*1*^ (107-129), *snr*^*101319*^ (BL11529), *osa*^*2*^ (107-130), *bap55*^*EY15967*^ (BL21174) *act5C*^*cg0010*^ (BL12083), *UAS-act5C* (BL24779), *Ay-Gal4* (107724) and *brm*^*2*^ (107-133) were from the Bloomington Drosophila stock center (Bloomington, USA), DGRC (Kyoto, Japan), or VDRC (Vienna, Austria). *w*^*m4*^ was segregated from BL6234 and outcrossed to the *w*^*1118*^. Heterozygous *su(var)2-5*^*5*^ and *lid*^*10424*^ mutants are used as controls for PEV suppressor and enhancer, respectively.

Fly stocks of *P[w*^*+*^*,HS-lacZ]65E* and *Tp(3;Y)BL2* (Joel C. Eissenberg, St Louis University), *UAS-HA-*tagged *brm*^*DN*^ and *UAS-ISWI*^*DN*^ (Fengwei Yu, National University of Singapore^23^), *bap170*^*Δ65*^*bap180*^*Δ86*^ and *brm*^*T362*^ (Jessica Treisman, New York University^14^) were generous gifts. *Tctp*^*h59*^, *Tctp*^*EY09182*^, genomic rescue transgene *Tctp*^*Ge30-2*^, *UAS-Tctp-RNAi* (*Tctp-i*), *UAS-Tctp*, and *UAS-Tctp*^*E12V*^ were as reported previously^26^. *Tctp*^*EY/h59*^ strong hypomorphs were generated by crossing *Tctp*^*EY09182*^*/TM6b* and *Tctp*^*h59*^*/TM6b* flies^22^. *ptc-Gal4* (second chromosome insertion) is used to express *UAS*-transgenes in SG tissue and AP boundary of wing discs.

Mutant clones were induced using the FLP/FRT system^66^. Larvae were heat-shocked for 1 h at 38 °C at either 24 h (*Tctp*^*EY09182*^ clone), 72 h (*brm*^*T362*^ clone), or 96 h (*Tctp*^*h59*^ clone) after egg deposit in the following genotypes:

*hs-FLP*/ (+ or *Y*); *FRT82B, Ubi-nGFP/FRT82B, Tctp^EY09182^, hs-FLP/* (+ or *Y*); *FRT82B, Ubi-nGFP/FRT82B, Tctp^h59^, hs-FLP/* (+ or *Y*); *FRT2A (=FRT79D-F), Ubi-nGFP/FRT2A, brm^T362^*.

To generate Flip-out clones in SG, larvae were heat-shocked for 5 min at 38 °C at 24-48 h after egg deposit in the following genotypes:

*hs-FLP*/(+ or *Y*); *UAS-Tctp-i/+; Ay-Gal4, UAS-GFP.S65T/+*

*hs-FLP*/(+ or *Y*); *Ay-Gal4, UAS-GFP.S65T/UAS-Brm^DN^::HA*

### Eye pigmentation assay

2–3 days-old adult male flies were used for measuring eye pigmentation. Eye pigments were extracted from 20 frozen heads in 0.5 ml of cold acidified methanol (absolute MeOH with 0.1% HCl) using Kontes pestle. After centrifugation for 10 min at 12,000 r.p.m., 4 °C, the cleared lysate was collected into a new 1.5 ml tube. Absorbance at 480 nm was measured. Quantification was performed in triplicates.

### Quantitative real-time PCR

Fly samples were frozen in liquid nitrogen and stored at –80 °C until use. Total RNA was prepared with Trizol reagent (Invitrogen, USA). Total RNA (2 μg) was reverse-transcribed using the QuantiTect reverse transcription Kit (Qiagen, Germany). Quantitative real-time PCR (QPCR) was performed using SYBR Green master mix on CFX96 Real-Time PCR System (Bio-Rad, USA) with standard cycling parameters (1 min at 95 °C and 40 cycles of 20 s at 95 °C, 20 s at 60 °C, and 45 s at 72 °C). C_T_ values for the detected mRNA levels of each gene were normalized to those of *rp49*. Mean expression levels were calculated from the values of three independent experiments, and were indicated as fold changes. Primer sets used for QPCR were listed in Supplementary Table 2.

### Immunohistochemistry and western blotting

SG and wing discs were fixed in PLP fixative (2% paraformaldehyde, 75 mM lysine, and 35 mM phosphate buffer, pH7.4) for 15–20 min at room temperature (RT). After brief washing two times in PBS for 10 min at RT, tissues were treated in blocking buffer (50 mM Tris-HCl, pH 6.8, 150 mM NaCl, 0.5% NP-40, and 5 mg per ml BSA) for 2 h at RT and incubated with primary antibody overnight at 4 °C in wash buffer (50 mM Tris-HCl, pH 6.8, 150 mM NaCl, 0.5% NP-40, and 1 mg per ml BSA). After washing four times in wash buffer for 40 min at RT, tissues were incubated with the appropriate secondary antibodies for 2 h at RT (1:300, Jackson immunoresearch). Primary antibodies were as follows: Mouse (Ms) anti-Fibrillarin (1:500, Abcam 4566), Rb (Rabbit) anti-H3K9me2 (1:200, Millipore 07-212), Rb anti-H3K9me3 (1:200, Abcam 8898), Ms anti-HP1a (1:50, DSHB C1A9-c), Rb anti-Tctp (1:1,000)^22^,^26^, Guinea Pig (GP) anti-Brm (1:400–500, y0866 from Dr. Fengwei Yu)^23^, FITC-Phalloidin (1:100, Sigma P5282), Rb anti-γ-H2Av (1:2,000, Rockland 600-401-914), Rat anti-HA (1:250, Roche 11867423). Chromosomes were visualized with DAPI. For confocal analysis, Zeiss LSM 710 was used.

For polytene chromosome staining^67^,^68^, third instar larvae were raised at 18 °C to obtain well-resolved band/interband pattern. Primary antibodies were as follows: Rb anti-Tctp (1:300), Ms anti-HP1a (1:50, DSHB C1A9-c), GP anti-Brm (1:200), Ms anti-Pol-II (1:50, Covance MMS-128P), Ms anti-Pol-IIa (1:50, Covance MMS-126R), Ms anti-Pol-II^Ser5^ (1:50, Covance MMS-134R), and Ms anti-Pol-II^Ser2^ (1:50, Abcam 24758). Gross bands intensity on each channel was measured using ROI (Region of interest) menu in ZEN program (Zeiss) and normalized to DAPI intensity value using Excel program (Microsoft).

For western blot analysis, 10 larvae or 20 pairs of third instar larval SG were lysed in SDS-gel leading buffer and analysed for each genotype. Primary antibodies were as follows: Rb anti-Tctp (1:20,000), Rb anti-Histone3 (1:10,000, Millipore 05-928), Ms anti-β-tubulin (1:5,000, DSHB E7-c), Ms anti-Fibrillarin (1:1,000, Abcam 4566), Ms anti-HP1a (1:1,000–2,000, DSHB C1A9-c), Ms anti-Pol-II (1:1,000, Abcam 5408), Ms anti-Pol-IIa (1:2,000, Abcam 817), Rb anti-Pol-II^ser5^ (1:2,000, Abcam 5131), and Rb anti-Pol-II^Ser2^ (1:2,000, Abcam 5095).

### Yeast two hybrid screen

The full length Tctp bait (171 aa) was cloned into EcoRI/SalI sites of pGBKT vector as a EcoRI/XhoI fragment. Y2H Screening for Tctp partners was performed in yeast PBN204 strain containing three selection markers (URA3, lacZ and ADE2) that are under the control of different GAL promoters (PanBioNet, Pohang, Korea). Yeast transformants of the Tctp bait and *Drosophila* embryo cDNA AD library were spread on selection medium (SD-leucine, tryptophan, uracil (SD-LWU)) that supports growth of yeasts with bait and prey plasmids yielding proteins interacting each other. After selecting yeast colonies on uracil-deficient media, these URA^+^ colonies were re-selected on adenosine-deficient media (SD-LWA).

### *In vitro* GST-pull down assay

MBP-tagged Tctp^22^ and GST-tagged Brm fragments (GST::Brm 1-311, 304-747, 748-1638, Δ304-500, Δ574-648, Δ694-747, ΔHSA, ΔBRK, and ΔHSA, ΔBRK) were cloned into pMAL-c2 (NEB) and pGEX5X-1 (GE healthcare), respectively. In-Fusion system (Clontech) was used for cloning. Primer sets used for cloning were listed in Supplementary Table 2. GST-tagged and MBP-tagged proteins were expressed in *E.coli* Rosetta2 (Novagen, Germany). IPTG (0.1 mM) induction was performed in 14 °C shaking incubator at 250 r.p.m. for 4 h. The extracted proteins were dialysed in TBS buffer (10 mM Tris-HCl (pH8.0), 150 mM NaCl) containing 20% glycerol and 1 mM DTT and stored at -20 °C before use.

Five micrograms of MBP fusion proteins were used as prey and the same amount of GST fusion proteins as baits. Protein complexes were formed in PDB (20 mM Tris-HCl (pH 7.5), 150 mM NaCl, 0.5 mM EDTA, 10% glycerol, 0.1% Triton X-100, 1 mM DTT, proteinase inhibitor cocktail (Roche)) containing 0.2% bovine serum albumin for 2–3 h at 4 °C. After washing three times in PDB at RT for 15 min, the samples were boiled in protein loading buffer at 94 °C for 5 min and loaded for western blotting. Primary antibodies were as follows: Ms anti-GST (1:4,000, SantaCruz sc-138) and Rb anti-MBP (1:10,000, NEB E8030S).

### Co-immunoprecipitation

To express 2xFLAG-tagged full length Brm derivatives (WT, ΔHSA, ΔBRK, and ΔHSAΔBRK) in *Drosophila* S2 cell, PCR products amplified from LD36356 (DGRC, IN, USA) were cloned into pAc5.1-N'-tagged 2xFlag vector (modified from pAc5.1 (Invitrogen, USA)) using In-fusion system (Clontech). For C-terminus-V5-tagged ARP4 and Act expression, these genes were PCR-amplified from LD29458 and RE02927, respectively, and cloned into pAc5.1 vector. Primer sets used for cloning were listed in Supplementary Table 2. 2xMYC-tagged Tctp is as reported previously^22^. For transfection, the amount of vector used per 1.2 × 10^6^ cells were as follows: 0.25 μg of pAc5.1-2xMYC-Tctp, 0.5 μg of pAc5.1-2xFLAG-Brm (WT or derivatives), 0.5 μg of pAc5.1-ARP4-V5, and 0.5 μg of pAc5.1-Act-V5.

S2 cells were transfected using X-tremeGENE HP DNA transfection reagent (Roche) according to the manufacturer's manual with some minor modifications (3:1 ratio for transfection reagent per target DNA and 30 min incubation for transfection complex formation). pAc5.1 empty vector was used to transfect an equal amount of plasmids. Stable cell line expressing 2xFLAG-Brm^WT^ were produced using pCoHygro (Invitrogen, 19:1=target:selection plasmids) and maintained in 10% AS (Sigma, I7267) containing M3 media with hygromycin B (Invitrogen, 10,687-010, 0.15 mg per ml).

The nuclear extract was obtained from S2 cells and treated with 100 U per ml of benzonase (EMD Millipore) for 1 h in IP buffer (20 mM HEPES (pH 7.4), 0.2 mM EDTA, 1.5 mM MgCl2, 1 mM DTT, 5% glycerol, 80 mM KCl, 0.2% NP-40, 2 × proteinase inhibitor cocktail and 1x phostop (Roche)). Protein complexes were immunoprecipitated with each 5 microgram of Rb anti-MYC (Abcam 9106), Ms anti-FLAG (Sigma F1804), or Ms anti-Pol-II (Abcam 5408) at 4 °C for 2 h. The immunocomplexes were washed three times with cold IP buffer at 4 °C. The samples were boiled in protein loading buffer at 94 °C for 5 min and loaded for western blotting. Primary antibodies were as follows: Rb anti-FLAG (1:1,000, Cell Signaling #2368), Ms anti-MYC (1:2,000, SantaCruz, sc47694), Rb anti-MYC (1:10,000, Abcam 9106), Rb anti-V5 (1:2,500, Sigma v8137), and Ms anti-Pol-II (1:1,000, Abcam 5408).

### *In vitro* ATPase assay

DNA-dependent ATPase activity was determined with a colorimetric assay measuring the formation of inorganic phosphate with some modifications^31^,^32^,^33^. Standard reaction mixtures (50 μl) contained 20 mM HEPES (pH 7.4), 0.2 mM EDTA, 1.5 mM MgCl_2_, 0.1 mM DTT, 5% glycerol, 80 mM KCl, 1 mM ATP, 20 μg ml^−1^ of SK(-) plasmid DNA, 2 × proteinase inhibitor cocktail (Roche). The reaction mixture was incubated at 30 °C for 30 min. Reaction was terminated by an addition of 0.85 ml of malachite green/acid molybdate/tween20 (0.04%) solution, followed by the addition of 0.1 ml of a 34% sodium citrate solution. After 15 min of color development, A660 was determined.

### Measuring eccDNA formation

Approximately 100 larvae were frozen in liquid nitrogen. Samples were ground with a Kontes pestle in 0.5 ml of Hirt lysis buffer (0.6% SDS, 10 mM EDTA, pH 8.0)^6^, and then incubated at RT for 10–20 min. NaCl was added to a final 1 M concentration and incubated overnight at 4 °C. After centrifugation at 14,000*g* for 40 min at 4 °C, the supernatant was collected into a new 1.5 ml tube and DNA was phenol-chloroform extracted three times and precipitated with ethanol. eccDNA content was assayed by QPCR and gel electrophoresis using appropriate primers (Supplementary Table 2).

### Heat shock

Third-instar larvae raised at 18 °C were transferred to 1.5 ml tubes containing a piece of wet 3MM paper and submerged in a 37 °C water bath for 30 min.

### Microarray analysis

Total RNA was extracted using TRIzol reagent (Life Technologies, Carlsbad, CA, USA) according to the manufacturer's protocol. Total RNA concentration and purity were determined by spectrophotometry to measure the absorbance ratio at 260:280 nm (RNA samples with ratios greater than 1.8 were used for analyses). The integrity of the RNA samples was also ascertained by analyzing 28S and 18S ribosomal RNAs with a Bioanalyzer 2100 (Agilent Technologies, Santa Clara, CA, USA). For gene expression profiles (eBiogen, Seoul, Korea), 500 ng RNA per sample was processed to labelled cRNA using the Affymetrix 3′ IVT Express kit according to the manufacturer's instructions and hybridized to Affymetrix Drosophila 2.0 GeneChips for 16 h at 45 °C. Gene chips were washed and stained with streptavidin-phycoerythrin using the Affymetrix Fluidics Station 450 and scanned on an Affymetrix GeneChip 3000 scanner (Affymetrix, Santa Clara, California, USA). The intensity values were normalized and summarized using robust multi-array analysis in R (www.R-project.org) and the Bioconductor package. A gene set representing >1.5-fold changes in at least one group (*Tctp*^*h59/+*^ or *Tctp*^*EY/h59*^) is presented by hierarchical clustering analysis (red, >1.5-fold change; green, <1.5-fold change). The hierarchical clustering was conducted using Agilent's GeneSpring GX7.3 (http://www.genomics.agilent.com/).

### Chromatin immunoprecipitation (ChIP) in S2 cells

Cells (3.7–6.3 × 10^7^) grown in a T25 flask were cross-linked with 1% formaldehyde for 10 min at room temperature. This cross-linking was stopped with 125 mM glycine for 5 min at room temperature. The DNA-protein complexes were prepared according to the manual for SimpleChIP enzymatic chromatin IP kit (Cell Signaling) without MNase treatment. The cross-linked chromatin was sheared into 200∼500 base pairs using Branson sonifier 450 (10% amplitude for 30 s with 0.5 s interval, five cycles). The DNA-protein complexes were incubated with each 2–3 μg of nonimmune IgG antibody, anti-PolII (Ab5408, 4H8), anti-H3 (Millipore 05-928), anti-H3K9me3 (Ab8898), anti-Brm (Dr Fengwei Yu) or anti-Tctp for overnight at 4 °C. Chromatin IP, bead-washing, and chromatin elution were performed according to the kit manual. Short DNA fragments corresponding to *su(var)3-9* locus were amplified using QPCR. The primer set is listed in Supplementary Table 2.

### Double-stranded RNA-mediated interference

One microgram of PCR products with T7 promoter sequence (*pSK(-)* or *Tctp* RNAi; Supplementary Table 2) was used for production of dsRNA (MEGAscript kit, Ambion). Bathing way was used for RNAi treatment. To maximize target protein reduction by *Tctp* RNAi, S2 cells were treated twice on day 0 and day 3 (or 4)^22^.

### Life span

Flies were outcrossed 5 times with *w*^*1118*^ to minimize genetic background effects. Life span was measured with 150–300 flies per genotype^69^. The life span of each genotype was measured at least in 8–15 separate vials (20 male flies a vial). Flies were cultured at 29 °C, 50% relative humidity, and 12:12 h light/dark cycle in an incubator. Food vials containing fresh standard corn-meal media were replaced every 2–3 days. Life span analyses was performed using PRISM 6 (GraphPad, USA) with the Kaplan–Meier method. Comparisons among groups were conducted using Log-rank test^70^.

### Data availability

The microarray data are available at http://www.ncbi.nlm.nih.gov/geo/ (Accession number: GSE75900). All other relevant datasets will be made available from the authors upon request.

## Additional information

**How to cite this article:** Hong, S.-T. & Choi, K.-W. Antagonistic roles of *Drosophila* Tctp and Brahma in chromatin remodelling and stabilizing repeated sequences. *Nat. Commun.* **7,** 12988 doi: 10.1038/ncomms12988 (2016).

## Supplementary Material

Supplementary Information Supplementary Figures 1-12, Supplementary Tables 1-2, Supplementary Discussion and Supplementary References
