# Supplementary material for: Antagonistic roles of Drosophila Tctp and Brahma in chromatin remodelling and stabilizing repeated sequences
Source: Nat Commun. 2016 Sep 30;7:12988. doi: 10.1038/ncomms12988 (PMC5056459; doi:10.1038/ncomms12988)
Supplement: Supplementary Information — Supplementary Figures 1-12, Supplementary Tables 1-2, Supplementary Discussion and Supplementary References [file ncomms12988-s1.pdf]

a

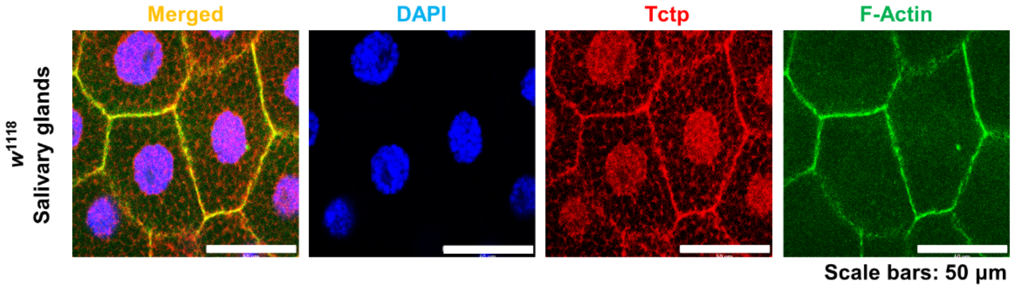

b

Tctp-interacting candidates in Y2H screen			
**CG number**	**Common gene name**	**Human ortholog**	**Known-Tctp interactant**
CG5949	*Brahma*	Yes	-
CG4303	*Bap60*	Yes	-
CG1276	*Transcription initiation factor*	Yes	-
CG34422	*Hat-trick*	Yes	-
CG8280	*Translation elongation factor, EF1A*	Yes	Yes
CG8282	*Snx6*	Yes	Yes
CG11154	*ATP synthase-β*	Yes	Yes
CG6770	*p8*	None	-
CG17127	-	None	-
CG11076	-	None	-

Supplementary Figure 1. Nuclear Tctp localization and Tctp-interacting candidates from yeast 2 hybrid (Y2H) screen.

(a) Tctp is co-localized with DAPI in the nucleus (blue) of salivary gland (SG) cells. Tctp is also distributed in a mesh-like pattern in the cytoplasm and is co-localized with filamentous actin (F-actin, green) in the cell cortex.

(b) From Y2H screen using Tctp as a bait, we obtained 14 positive clones, of which 10 candidates were listed. Three clones were out of frame and the other one is under further study. Besides well-known Tctp-interacting partners such as EF1A[1], SNX6[2] and ATP synthase[3], several novel candidates were obtained. Both Brahma (Brm) and Bap60 are subunits of the Brahma chromatin remodeling complex. Interaction with nuclear proteins such as transcription initiation factor and Hat-trick (chromodomain protein) also implies nuclear functions of Tctp.

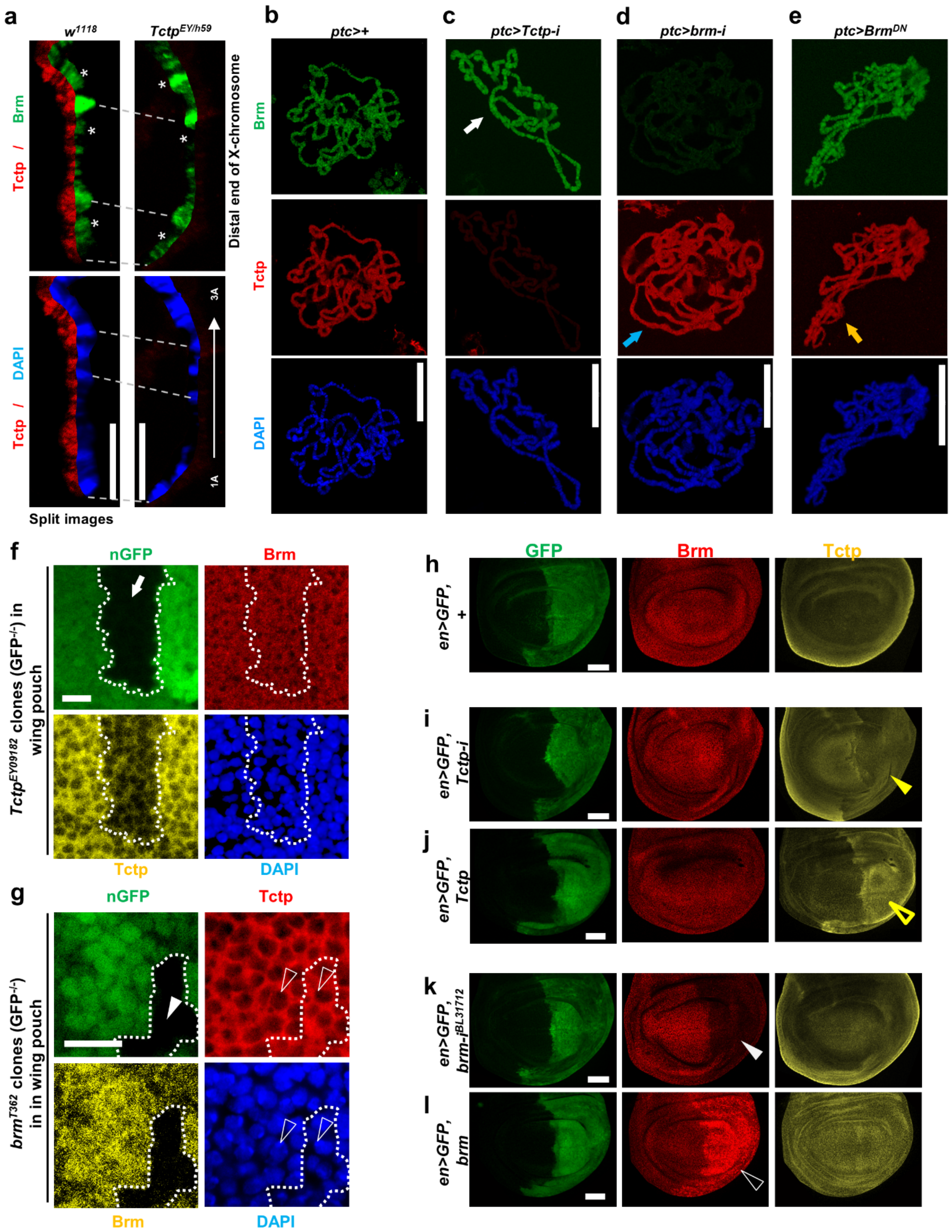

Supplementary Figure 2. Tctp does not affect the expression level or stability of Brm protein, and *vice versa*.

(a) Split images of a distal region in X-chromosome. Despite the similar gross level of Brm staining, *Tctp*$^{EY/h59}$ mutant chromosomes show some variation in the banding intensity (asterisks). Scale bars: 10 µm.

(b-e) Tctp and Brm are independently recruited to SG polytene chromosomes.

patched (*ptc*)-*GAL4* driver is used to knockdown Tctp or overexpress dominant-negative defective Brm

(Brm^{DN}) in SG cells. (a) Control chromosome from *ptc*>+ larvae. (b) Tctp-Knockdown by RNAi (*ptc>Tctp-i*) does not affect Brm recruitment (white arrow) in squashed polytene chromosomes. (c-d) Brm-knockdown (*ptc>brm-i*) or Brm^{DN} overexpression (*ptc>*Brm^{DN}) also does not affect Tctp recruitment (blue and orange arrows, respectively). Scale bars: 50 µm.

(f-g) Mitotic clones (nuclear GFP (nGFP)-negative) of strong hypomorphic $Tctp^{EY09182}$ (arrows) or brm^{T362} null allele (arrowheads) in the developing wing pouch region. (f) Brm protein levels in cell nuclei (red). Note that the black spots unstained by Brm antibody are centromere regions. This Brm pattern is not changed in *Tctp* mutant clones (dotted area). (g) Tctp staining (red) is enriched around the cortex. Weaker staining inside the cortex matches with the area of DAPI staining (blue). More intense DAPI staining within the nucleus indicates the position of the centromere where Tctp staining is the weakest (open arrowheads). Overall the pattern of Tctp is not changed in *brm* mutant clones (dotted area). Scale bars, 10 µm. Dorsal side is up.

(h) *en-GAL4* driver is used for knockdown by RNAi or overexpression in the posterior compartment of wing disc. In control wing disc carrying *en>GFP*, protein levels of Tctp (red) or Brm (yellow) are similar in both anterior and posterior compartments.

(i-j) Brm protein level (red) is not affected by Tctp-knockdown (*Tctp-i,* yellow arrowhead in B) or overexpression (open yellow arrowhead in c) in the posterior compartment.

(k-l) Tctp protein level (yellow) is not affected by *brm*-knockdown (*brm-i*, white arrowhead in D) or overexpression (open white arrowhead in E) in the posterior compartment. Anterior is left and dorsal is up in all panels. Scale bars, 50 µm.

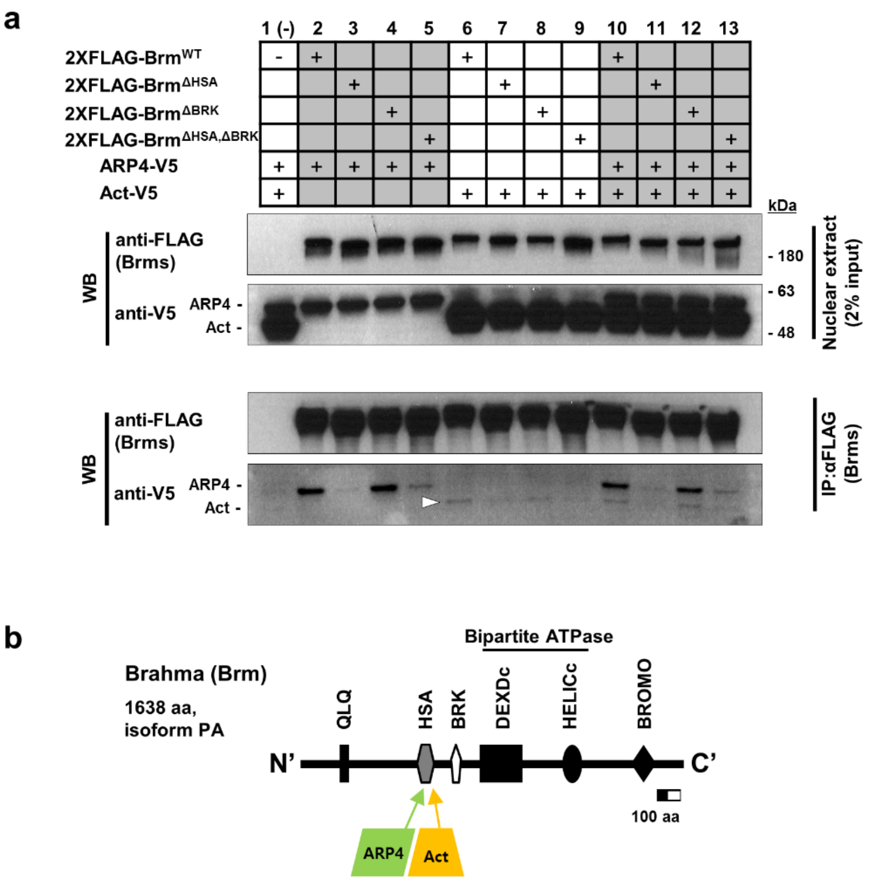

Supplementary Figure 3. HSA domain of Brm is required for binding both ARP4 and Actin.

(a) Nuclear extracts from S2 cells expressing ARP4-V5, Act-V5, or both with FLAG-tagged full-length Brm derivatives without HSA (△HSA), BRK (△BRK), or both (△HSA,△BRK) were immunoprecipitated using FLAG-antibody. Actin and ARP4 proteins are known as the core subunits of Brm chromatin remodeling complex [4, 5, 6, 7]. Arrowhead indicates the immunoprecipitated Act protein by Brm proteins.

(b) A schematic drawing of results shown in panel a. ARP4 and Act bind to the HSA domain of Brm ATPase subunit. DEXDc and HELICc domains indicate a bipartite structure of the ATPase domain.

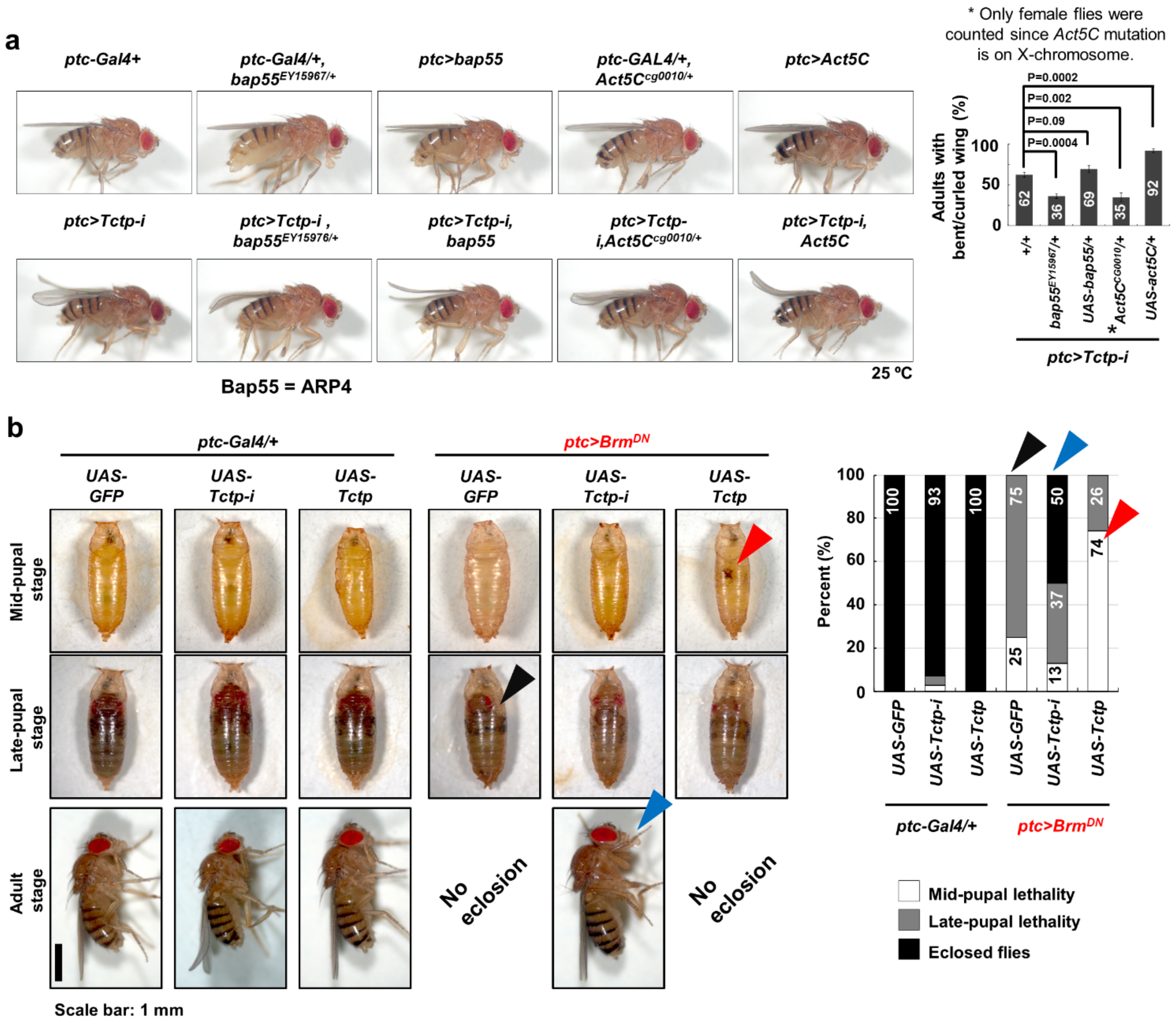

Supplementary Figure 4. Genetic interaction between *Tctp* and *Brm* complex subunit genes.

(a) Genetic interaction between *Tctp* and a subset of core subunit genes of Brm complex (*bap55* and *Act5C*). *bap55* is a synonym for *ARP4*. Results are quantified and presented as a histogram. n=220-423 flies. Mean±s.d.

(b) Genetic interaction between *Tctp*-knockdown and *brm* dominant-negative mutation with catalytically inactive ATPase (*Brm^{DN}*). Overexpression of Brm^{DN} (*ptc>Brm^{DN}*) causes of mid-pupal (25%) or late-pupal lethality (75%) (black arrowheads).This pupal lethality is considerably suppressed by *Tctp*-knockdown by RNAi (13% mid-pupal and 37% late-pupal lethality, respectively). Moreover, no-eclosion phenotype of *ptc>Brm^{DN}* is noticeably suppressed (blue arrowheads). In contrast, Tctp overexpression in *ptc>Brm^{DN}* flies exacerbates the lethality (red arrowheads), resulting in about 3 times higher mid-pupal lethality. n=100-391 flies.

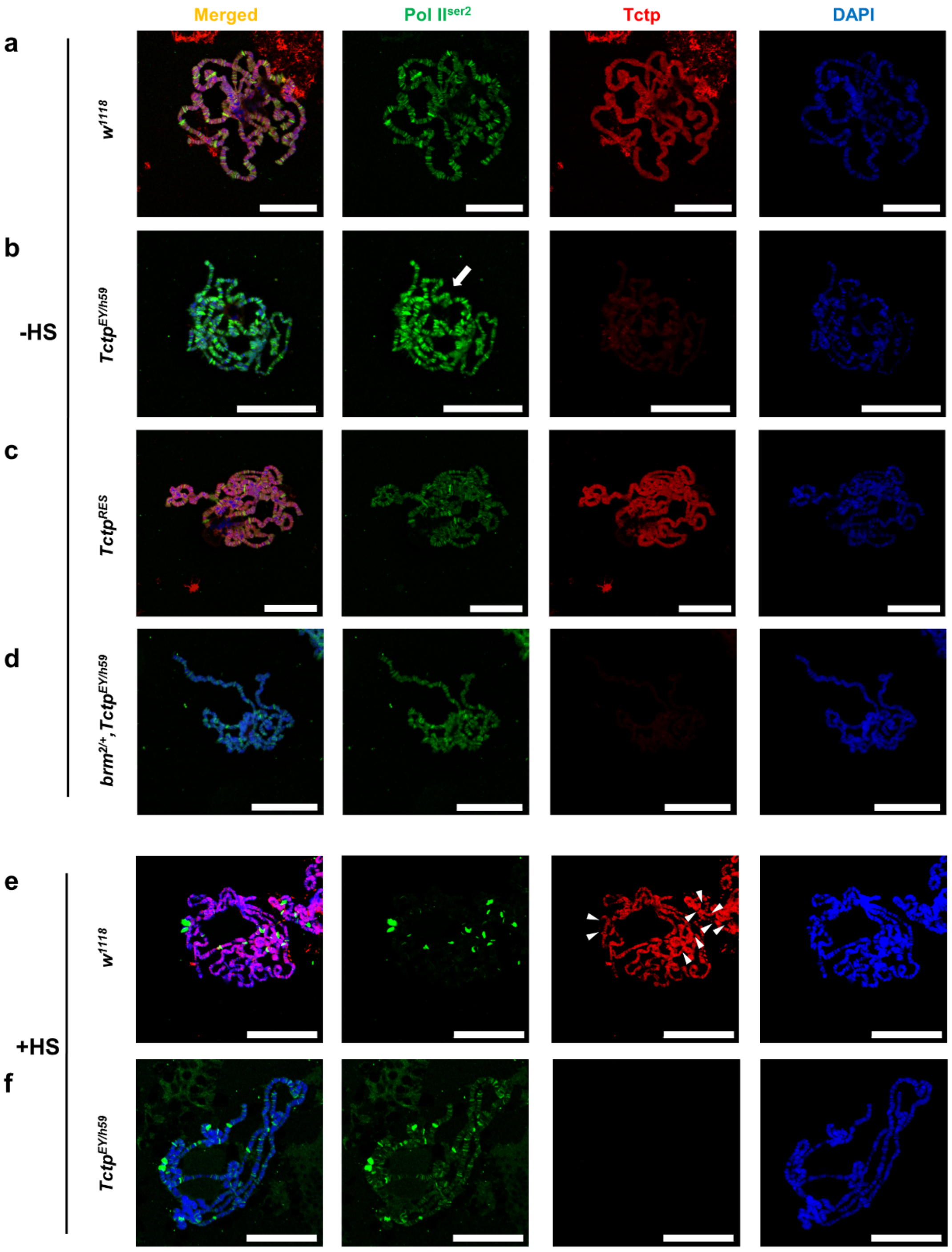

Supplementary Figure 5. Increased transcription elongation in *Tctp*$^{EY/h59}$ mutant.

(a-d) The level of phosphorylated RNA Pol-II at serine 2 residue of CTD (Pol-II^{ser2}) is strongly increased in salivary gland (SG) polytene chromosomes from *Tctp*$^{EY/h59}$ mutant (white arrow in B) compared with wild-type control or genomic rescued *Tctp*$^{EY/h59}$ mutant (*Tctp*^{RES}). Scale bars, 50 µm.

(e-f) After heat-shock (+HS), elongating Pol II dissociates from euchromatin sites and accumulates at heat-shock loci in wild-type. In *Tctp*$^{EY/h59}$ mutant, a substantial amount of elongating Pol-II persists in some interband or euchromatin sites after HS (f). Note that Tctp is not recruited to most heat-shock loci (e, white arrowheads). This pattern of Tctp is similar with that of Brm, which is not required for heat-shock loci formation [8]. Scale bars, 50 µm.

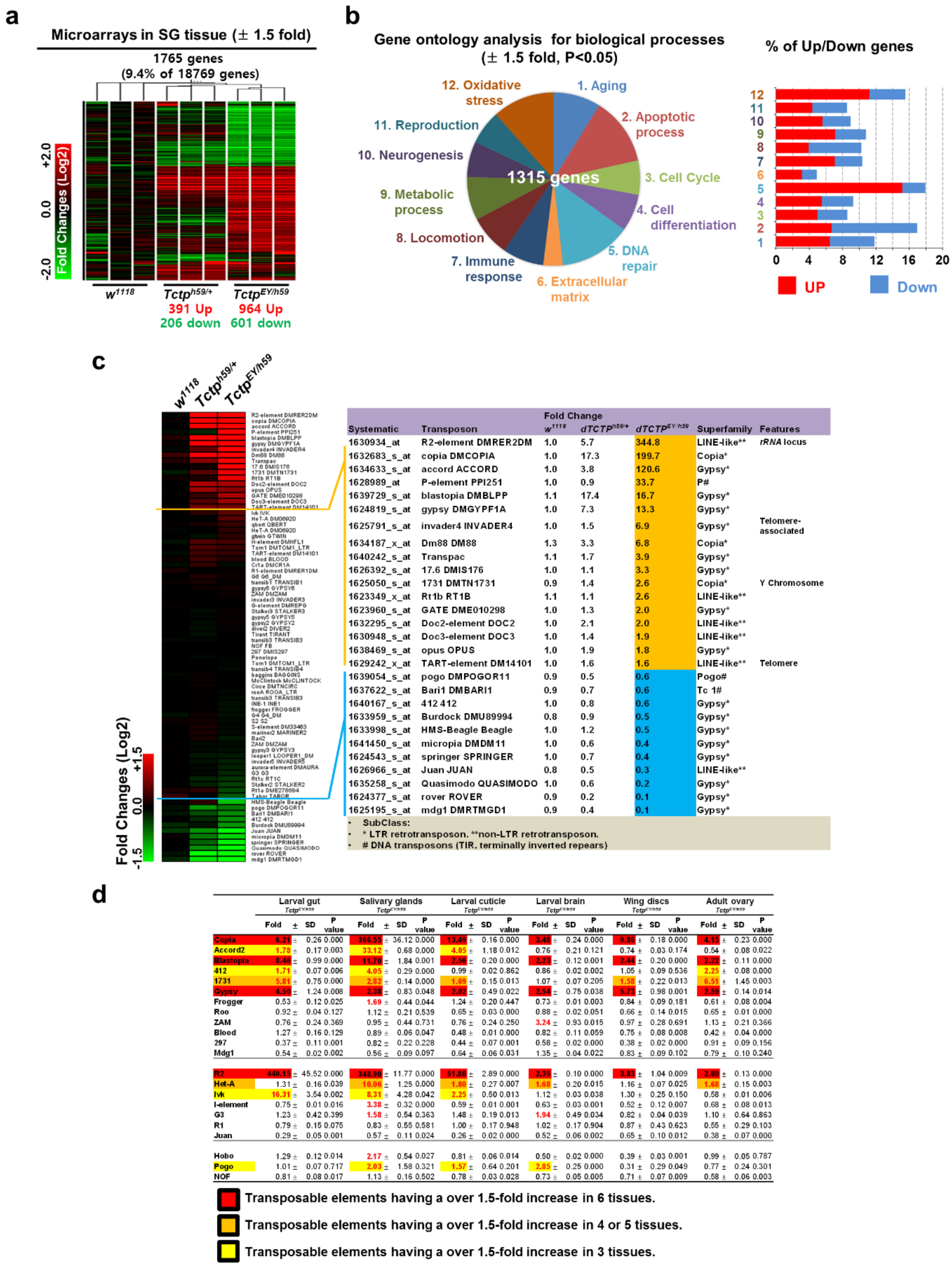

Supplementary Figure 6. Transposon expression profiling in third instar larval salivary gland cells of *Tctp* mutants.

(a) mRNA profiling in *Tctp* mutants. Total RNA was prepared using salivary gland (SG) tissues from w^{1118}, $Tctp^{h59}$/+ heterozygotes, and $Tctp^{EY/h59}$ transheterozygote. Each genotype was processed in three independent replicates. Heatmap represents fold changes relative to w^{1118} control. 1765 genes showed more than 1.5-fold changes in expression by *Tctp* mutation.

(b) Gene ontology analysis of 1315 genes showing more than 1.5-fold expression changes (P<0.05) in $Tctp^{EY/h59}$ mutants.

(c) Transposon expression profiling in *Tctp* mutants. Increased or decreased expression was indicated in the right table by yellow or blue, respectively. Heat maps show the fold change of transposon expression from microarray analysis. 17 out of 78 transposons are up-regulated over 1.5 fold in salivary gland cells of $Tctp^{EY/h59}$ mutants compared with wild-type. Note that the more Tctp is deleted, the more transposons are expressed ectopically (left panel). High levels of ectopic expression are shown in transposons residing in typical heterochromatic regions of chromosome such as some parts of *rRNA* loci, telomere, or Y chromosome (right table). Fold changes in *Tctp* mutants are estimated relative to w^{1118} control (n=3, # of tests).

(d) QPCR validation in various tissues. Transcript levels were normalized to *rp49* and shown as fold changes relative to w^{1118} control (mean ± SD, n=3 (# of tests)).

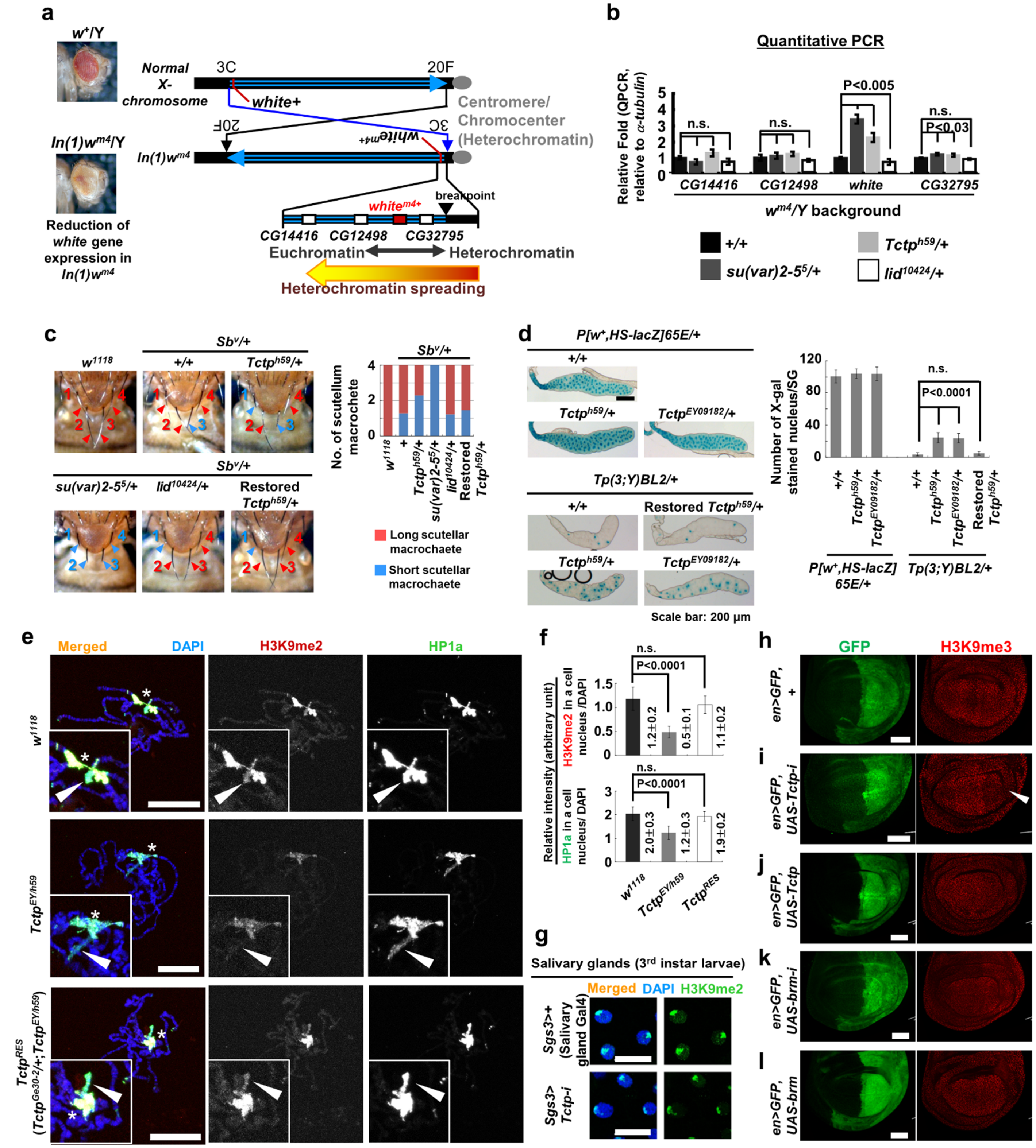

Supplementary Figure 7. Reduced pericentromeric heterochromatin silencing in *Tctp* mutants.

(a-d) PEV phenotypes are suppressed by *Tctp*^{h59}/+ mutation.

(a-b) *Tctp*^{h59}/+ mutation increases *white* gene expression suppressed by w^{m4} PEV.

(a) A schematic representation of the *white* locus at the w^{m4} inversion. A large inversion transposes the *white* gene next to the pericentromeric heterochromatin of the X chromosome (*white*^{m4}) [9]. This rearrangement results in the characteristic variegated phenotype shown in left lower panel. Counteraction of the heterochromatin spread at the boundary (around breakpoint, ▼) maintains the *white* gene expression. The genes next to the *white* gene are shown schematically.

(b) Heterozygous *Tctp*^{h59} mutation causes derepression of the *white* gene in the head tissues of *w*^{m4} flies. Total RNA was prepared from the fly heads, and mRNA expression levels were measured. Relative expression levels were normalized to *α-Tubulin* mRNA. Gene expression in *w*^{m4}-only males (+/+) was used as a control for fold change of expression (mean ± s.d., n=3 (# of tests)). n.s., not significant ($P \geq 0.1$).

(c) The effects of heterozygous *Tctp*^{h59} mutation on *Stubble* variegation of *T(2;3)Sb*^{v}. Red or blue numbers indicate normal (long) or shortened scutellar bristles, respectively. *Sb*^{v}/+ shows a bristle PEV phenotype. *Tctp*^{h59}/+ suppresses the *Sb*^{v}/+ phenotype. *su(var)2-5*^{5}/+ and *lid*^{10424}/+ suppress or enhance the *Sb*^{v}/+ PEV, respectively. The heterozygous *Tctp*^{h59} mutant having a genomic *Tctp* fragment (*Tctp*$^{Ge30-2}$) as a transgene shows a rescue of the *Sb*^{v}/+ PEV. **(Histogram)** Quantification of the number of long or short scutellar bristles (n=30-60 flies, mean±s.d.).

(d) The effects of heterozygous *Tctp*^{h59} mutations on *Tp(3;Y)BL2* variegation line. Salivary glands (SG) from third instar larvae were stained with X-gal. *P[w*$^{+}$*,HS-lacZ]65E* is a founder transgenic line of *Tp(3;Y)BL2* with a P-element inserted in the euchromatic region (65E) of the third chromosome and exhibits uniform *lacZ* expression in *Tctp*$^{+}$ wild-type or *Tctp/*+ heterozygote mutant backgrounds. The Y chromosome is heterochromatic in somatic tissues, thus showing strong suppression of *lacZ* expression by PEV. *Tctp*^{h59}/+ or *Tctp*^{EY09182}/+ suppresses the PEV effect of *Tp(3;Y)BL2. Tctp*$^{Ge30-2}$ restores the effect of *Tctp* mutations. **(Histogram)** Quantification of the number of X-gal stained cell nuclei per SG in indicated genotypes (n=20 SGs, mean±s.d.). Scale bar, 200 µm.

(e-l) Reduction of silencing marks in *Tctp* mutants.

(e, f) Levels of H3K9me2 and HP1a in polytene chromosomes from SG cell nuclei. The arrowhead indicates the 4th chromosome adjacent to the heterochromatic chromocenter (asterisk). Note that silencing mark levels are reduced in both chromocenter and the 4th chromosome from *Tctp*$^{EY/h59}$ mutant. (f) Both H3K9me2 and HP1a levels are quantified (n=14 SGs, mean ± s.d.). Scale bars, 50 µm.

(g) Salivary tissue-specific *Tctp* RNAi (*Sgs3>Tctp-i*) in third instar larvae causes a reduction of H3k9me2 level. Scale bars, 50 µm.

(h-l) *Tctp*-knockdown causes a reduction of H3K9me3 level in wing disc. *UAS*-RNAi or *UAS*-transgene driven by *en-Gal4* is used for knockdown or overexpression of target genes (Tctp or Brm) in the posterior compartment cells of wing disc, respectively. The posterior compartment marked by GFP is to the right. H3K9me3 levels (red) in both anterior and posterior compartments of *en>GFP*, *en>TCTP*, *en>brm-i*, and *en>brm* are similar (h, j, k, and l), while the posterior compartment of *en>Tctp-i* shows a reduced H3K9me3 level (i, arrowhead) compared with its anterior compartment (internal control). Scale bars, 50 µm.

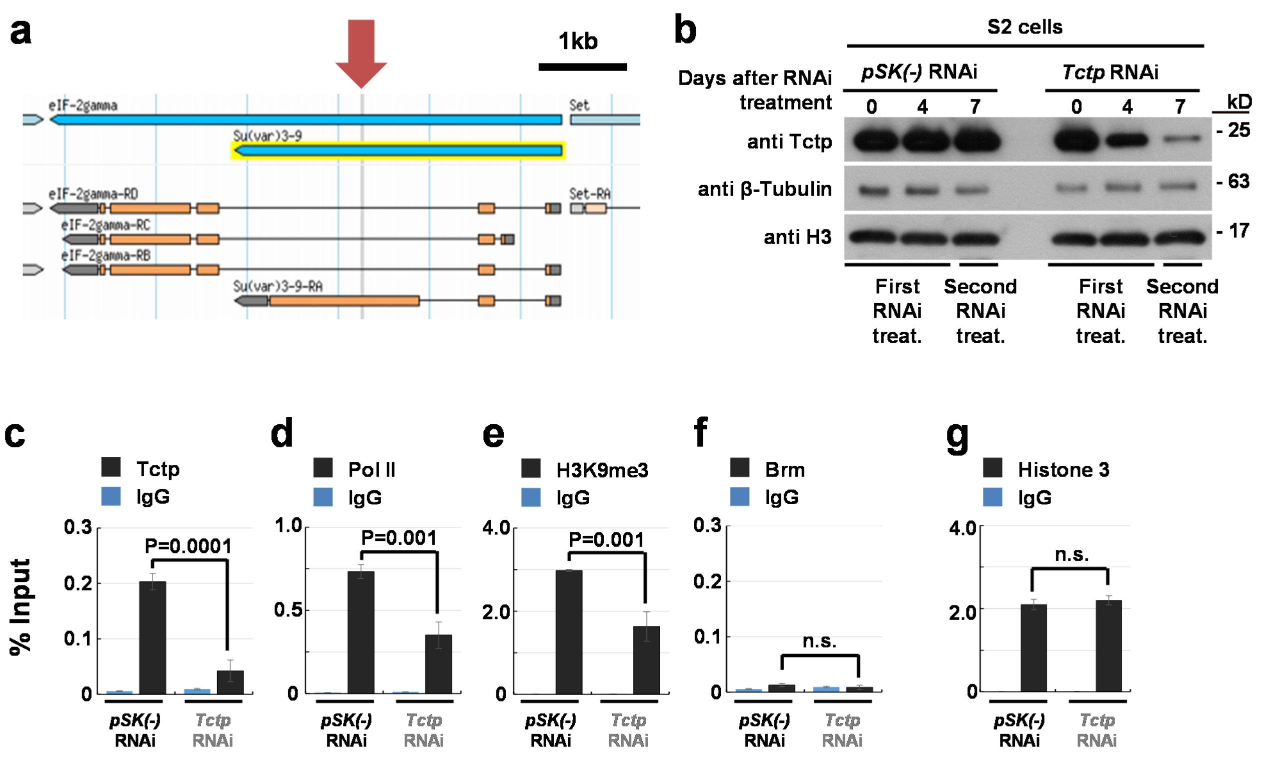

Supplementary Figure 8. Tctp is required for Pol-II association with *su(var)3-9* locus.

(a) Schematic of *su(var)3-9* locus (Flybase) and a primer set (red arrow) used for chromatin immunoprecipitation (ChIP).

(b) In S2 cells, double treatments of *Tctp* dsRNA (double-stranded RNA) efficiently reduced Tctp protein level based on western blot assay on day 7. The same amount of dsRNA for *pBluescript SK(-)* vector (*pSK(-)*) was used as a negative control. 50 µg of dsRNA was added for a T25 flask culture. Both β-Tubulin and Histone 3 (H3) were used as a loading control.

(c-g) ChIP analysis using S2 cells shows that Pol-II association with *su(var)3-9* locus is significantly reduced by Tctp-knockdown (c and d), compared with plasmid *pSK(-)* dsRNA control. The H3K9me3 level is also significantly reduced. (f) Note that the level of Brm associated with *su(var)3-9* locus is very low (comparable to IgG control) and not affected by Tctp-knockdown. (g) Positive control using Histone 3 antibody. n.s. (P=0.1 in f and P=0.2 in g).

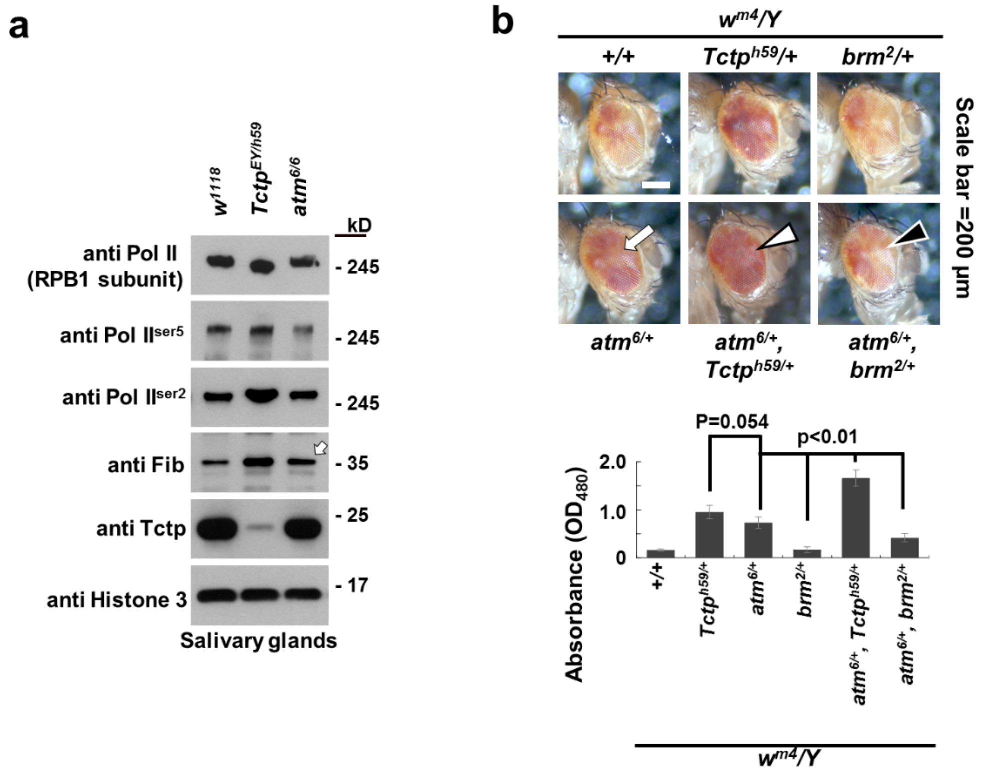

Supplementary Figure 9. Transcriptional and pericentromeric regulations in *Drosophila atm* mutants.

(a) Transcriptional regulation of Pol-I and Pol-II in *atm*^{6} mutants.

In mammalian cells, ATM inhibits both Pol-II transcription close to induced-DSB sites ("local"), and Pol-I transcription proximal and distal from DSB sites ("pan-nuclear"), responsing to dsRNA breaks (DSBs)[10, 11]. In larval SGs of homozygous *atm*^{6} mutant, Pol-II activity (Pol-II-Ser5 and Ser2) is comparable to wild-type control, while Pol-I activity (arrow in anti-Fib) is modestly increased. Interestingly, *Tctp*$^{EY/\mathrm{h59}}$ mutants show increases in both global Pol-II and Pol-I activities, compared with wild-type control. These results suggest that, in developing *Drosophila* SG tissues, *Drosophila* ATM preferentially inhibits Pol-I transcriptional activity in a pan-nuclear scale than Pol-II activity, and that Tctp has much broader effects on transcriptional regulation process than ATM does in *Drosophila*.

(b) Defects in pericentromeric heterochromatin stability (or heterochromatin spreading) in *atm* mutants.

w^{m4} PEV phenotype in *atm*^{6} heterozygous mutants was assayed. Similar to *Tctp*$^{h59/+}$ muatnts, *atm*^{6}/+ mutation suppresses the eye color phenotype in *w*^{m4} (arrow). In addtion, heterozygous *brm*^{2} mutation suppresses the *w*^{m4} PEV phenotype of *atm*^{6}/+ (black arrowhead), suggesting that defective heterochromatin spreading or pericnentromeric instability in *atm* mutants may be due to an excess Brm activity. Note that the PEV phenotype in *atm*$^{6/+}$,*Tctp*$^{h59/+}$ double mutants seems to be additive rather than synergistic (white arrowhead and histogram).

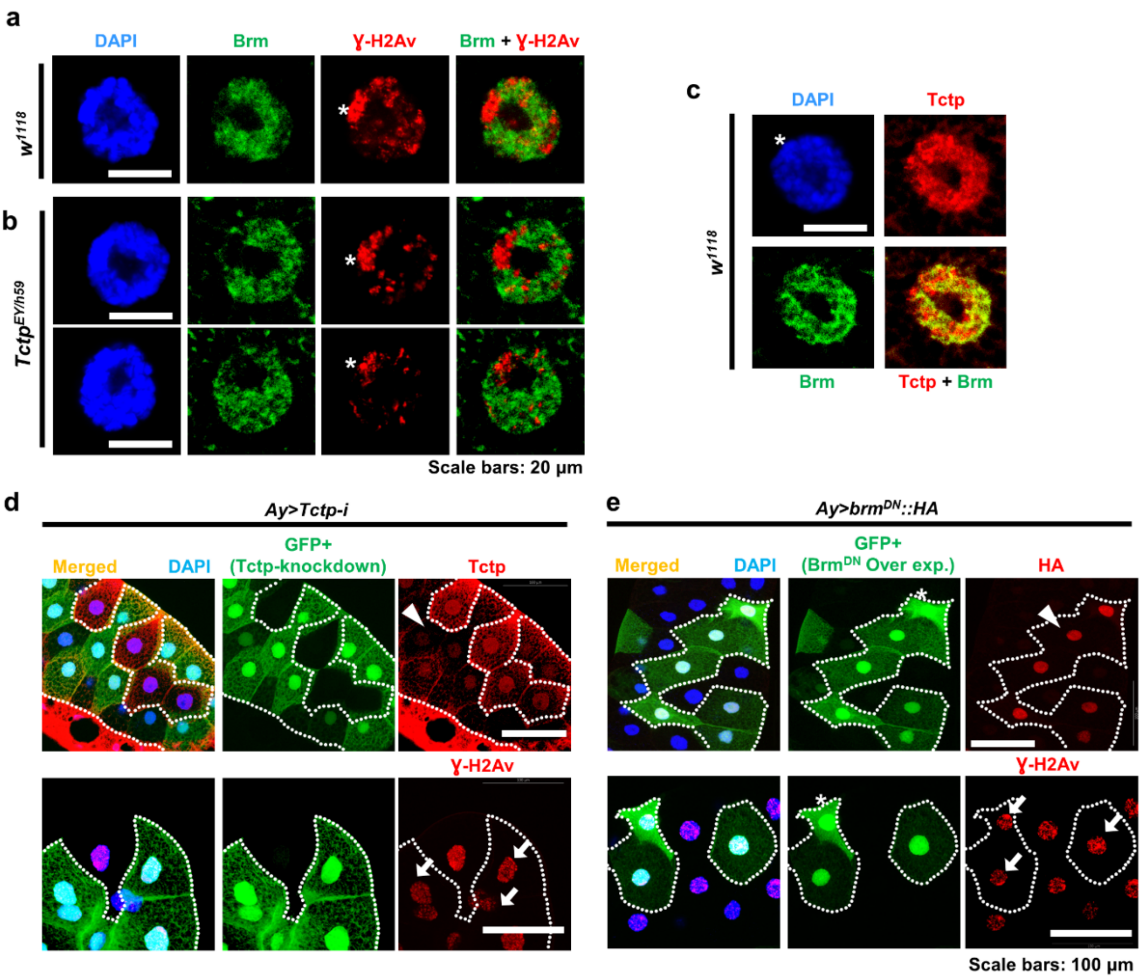

Supplementary Figure 10. DSB formation in salivary gland cells with reduced or defective Brm function.

(a-b) Most Brm proteins (green) in SG nucleus are not overlapped with γ-H2Av foci (red). γ-H2Av antibody is used to stain double stranded DNA breaks (DSBs).

(a) DSB repair foci of γ-H2Av are observed in the chromocenter region (asterisks) and multiple other sites in the nucleus[12].

(b) As previously reported[13], γ-H2Av level (red) is considerably reduced in SG nucleus from $Tctp^{EY/h59}$ mutants, compared with wild-type control. On the contrary, Brm level (green) in *Tctp* mutants is comparable to wild-type control.

(c) Tctp and Brm colocalization in larval SG nucleus. In developing SG tissues, most Tctp proteins colocalize with Brm, while some Tctp staining do not. Scale bar, 20 µm.

(d) Flip-out Tctp-knockdown clones in SG tissue (GFP^+ cells). Gal4 flp-out system (*act>y*$^+$*>Gal4, UAS-GFP*) is used to generate clones. **(Upper panels)** Tctp-reduction (arrowhead) in flp-out Tctp-knockdown clones (GFP^+ cells). **(Lower)** Reduced levels of γ-H2Av (arrows) in flp-out Tctp-knockdown clones (GFP^+ cells).

(e) Flp-out Brm^{DN} clones in SG tissue (GFP^+ cells). **(Upper)** HA-tagged dominant-negative form of Brm (Brm^{DN}) is over-expressed in flp-out clones (arrowhead). Notable cell size reduction is frequently observed in some flp-out Brm^{DN} clones (asterisks). **(Lower)** γ-H2Av levels in flp-out Brm^{DN} clones (arrows) are similar with adjacent GFP-negative control clones.

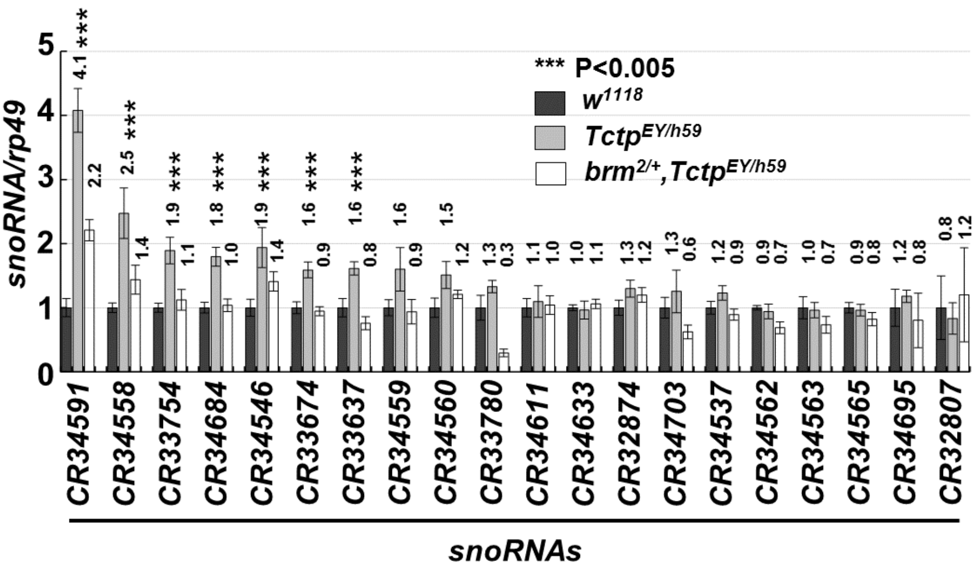

Supplementary Figure 11. QPCR analysis on snoRNA (small nucleolar RNA).

Increased expression of a subset of snoRNAs in *Tctp*$^{EY/h59}$ mutants is reduced by heterozygous *brm*^{2} mutation.

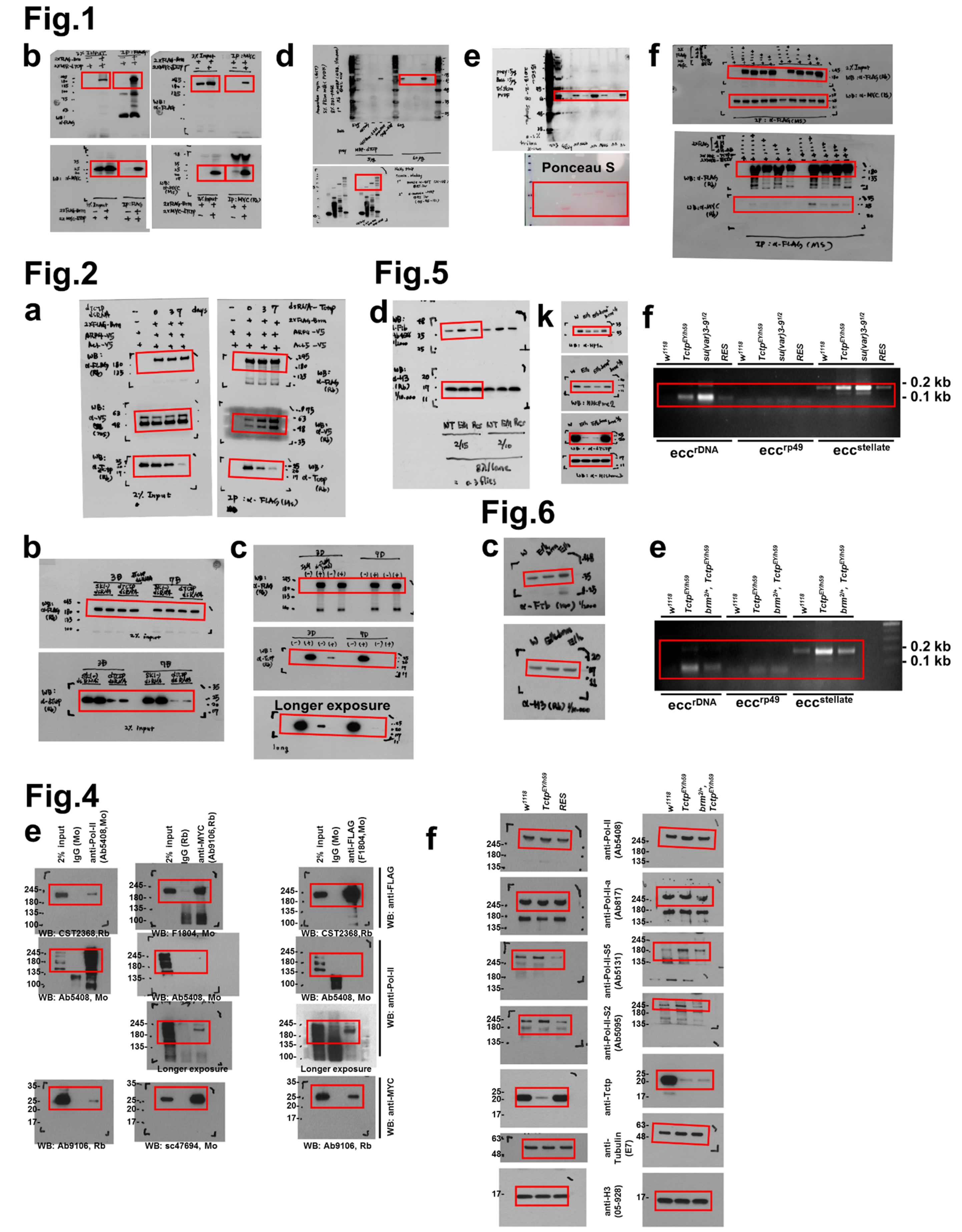

Supplementary Figure 12. Full-length pictures of the blots and gels presented in the main figures

Supplementary Table 1. Gene Ontology analyses.

Systematic ID	Transcript ID	Gene Title	Fold change	t-test p-value
**Ageing**				
1623256_at	CG5164-RA	Glutathione S transferase E1	9.331	0.001191725
1630487_s_at	CG32041-RA	Heat shock protein 22 /// Heat shock gene 67Bb	9.304	2.21E-05
1630593_at	CG33126-RA	Neural Lazarillo	3.415	0.019701043
1633471_at	CG11765-RA	Peroxiredoxin 2540-2	2.934	0.017057572
1639480_at	CG6871-RA	Catalase	2.592	0.009853024
1633391_at	CG8913-RA	Immune-regulated catalase	2.184	0.04989941
1639526_a_at	CG7717-RA	CG7717 gene product from transcript CG7717-RB	2.109	0.003677665
1638844_s_at	CG3714-RC	CG3714 gene product from transcript CG3714-RG	2.107	4.67E-04
1634219_a_at	CG1633-RB	thioredoxin peroxidase 1	1.935	0.005328337
1628352_a_at	CG8798-RB	Lon protease	1.669	0.002491571
1628257_s_at	CG12013-RB	CG12013 gene product from transcript CG12013-RC	1.636	0.016961023
1630941_s_at	CG16944-RA	stress-sensitive B	1.613	0.001486399
1639507_at	CG6666-RA	Succinate dehydrogenase C	1.538	9.02E-04
1636974_at	CG32146-RA	dally-like	0.634	0.036913905
1625048_at	CG8318-RA	Neurofibromin 1	0.578	0.030962145
1633290_a_at	CG4482-RA	moladietz	0.456	0.00841207
1640097_at	CG12002-RA	Peroxidasin	0.437	0.03795444
1625276_a_at	CG7266-RC	Ecdysone-induced protein 28/29kD	0.312	0.00480965
**Apoptosis process**				
1640235_at	CG5123-RA	Wrinkled	2.214	6.14E-04
1631493_at	CG1548-RA	CG1548 gene product from transcript CG1548-RA	2.152	0.030961603
1639526_a_at	CG7717-RA	CG7717 gene product from transcript CG7717-RB	2.109	0.003677665
1634344_at	CG4180-RA	lethal (2) 35Bg	1.903	7.26E-04
1622960_at	CG14902-RA	death executioner caspase related to Apopain/Yama	1.901	0.04233018
1625038_s_at	CG10895-RA	loki	1.776	2.84E-04
1636274_at	CG6531-RA	wengen	1.745	0.009502802
1624450_at	CG6331-RA	Organic cation transporter	1.722	0.016746026
1631007_at	CG5371-RA	Ribonucleoside diphosphate reductase large subunit	1.616	0.003517713
1627249_a_at	CG4180-RB	lethal (2) 35Bg	1.565	3.03E-04
1631137_at	CG18593-RA	viral IAP-associated factor	1.513	0.001276583
1633089_a_at	CG12052-RJ	longitudinals lacking	0.636	9.38E-04
1628921_s_at	SD11801	modifier of mdg4 /// CG42769 gene product from transc	0.635	0.004457435
1628562_s_at	CG32491-RT	modifier of mdg4 /// CG42769 gene product from transc	0.632	2.57E-04
1623411_at	CG12052-RN	longitudinals lacking	0.628	0.003327923
1631595_at	CG13393-RA	lethal (2) k12914	0.621	0.009879119
1625283_at	CG32491-RM	modifier of mdg4	0.607	0.010529318
1639308_at	CG32491-RF	modifier of mdg4	0.565	0.001798739
1631742_s_at	CG32491-RQ	modifier of mdg4	0.537	0.00332743
1633152_at	CG32491-RA	modifier of mdg4	0.523	0.005434811
1638041_at	CG32491-RH	modifier of mdg4	0.516	0.012785054
1636765_at	CG32491-RJ	modifier of mdg4	0.516	0.005565568
1627953_at	CG32491-RI	modifier of mdg4	0.440	0.004287487
1631903_at	CG14062-RA	CG14062 gene product from transcript CG14062-RB	0.436	0.010198534
1634052_s_at	CG32491-RA	modifier of mdg4	0.408	0.00108066
1622976_at	CG32491-RB	modifier of mdg4	0.389	0.00638984
1627483_at	CT35609	modifier of mdg4	0.375	0.003102537
1631500_at	CG32491-RR	modifier of mdg4	0.308	1.91E-04
**Cell cycle**				
1624897_at	CG1558-RA	kinetochore Mis12-Ndc80 network component 1	4.842	0.002342818
1636189_at	CG12306-RA	CG12306 gene product from transcript CG12306-RA	3.172	0.002657874
1639036_at	CG8857-RC	Ribosomal protein S11	2.797	0.003441904
1630729_at	CG6535-RA	telomere fusion	2.788	1.16E-04
1641600_at	CG9282-RA	Ribosomal protein L24	2.680	1.49E-06
1638804_a_at	CG40293-RB	Ste20-like kinase	2.622	0.00200459
1627380_at	CG9633-RA	Replication Protein A 70	2.356	9.89E-05
1626425_at	CG4173-RA	Septin 2	2.331	3.96E-04
1637144_a_at	CG1483-RA	Microtubule-associated protein 205	2.185	2.40E-04
1639162_at	CG6072-RA	sarah	2.174	0.010623534
1638175_at	CG1424-RA	misato	2.010	4.82E-04
1631388_at	CG8432-RA	Rab escort protein	1.972	3.67E-04
1629752_at	CG5525-RA	CG5525 gene product from transcript CG5525-RA	1.961	9.10E-05
1639335_at	GM06790	pumilio	1.941	0.001499321
1633951_at	CG7769-RA	piccolo	1.923	3.51E-04
1630390_at	CG5602-RA	DNA ligase I	1.880	0.00708321
1639469_a_at	CG9441-RB	Punch	1.855	0.034885757
1639509_at	CG12396-RA	CG12396 gene product from transcript CG12396-RA	1.832	0.015157592
1630408_s_at	CG7033-RC	CG7033 gene product from transcript CG7033-RA	1.811	0.002246746
1625038_s_at	CG10895-RA	loki	1.776	2.84E-04
1624419_a_at	CG8439-RA	T-complex Chaperonin 5	1.775	0.002378092
1632291_at	CG6625-RA	Soluble NSF attachment protein	1.762	0.005031917
1624193_s_at	CG3983-RB	nucleostemin 1	1.748	0.002982253
1634700_at	CG8258-RA	CG8258 gene product from transcript CG8258-RA	1.686	0.002697775
1639118_a_at	CG12749-RA	Heterogeneous nuclear ribonucleoprotein at 87F	1.678	0.01084942
1632418_s_at	CG9755-RA	pumilio	1.670	0.007546981
1626845_at	CG8351-RA	CG8351 gene product from transcript CG8351-RA	1.656	3.33E-04
1633353_s_at	CG18408-RI	CG18408 gene product from transcript CG18408-RI	1.649	0.022984264
1625856_at	CG10798-RA	diminutive	1.642	0.014672076
1632043_a_at	CG9198-RA	shattered	1.640	0.012816613
1632946_at	CG40293-RA	Ste20-like kinase	1.638	0.014160253
1637091_a_at	CG9772-RB	CG9772 gene product from transcript CG9772-RA	1.621	0.013776829
1634695_at	CG2684-RA	lodestar	1.609	0.004681077
1631993_s_at	CG6058-RB	Aldolase	1.599	0.006915187
1626125_at	CG3157-RA	gamma-Tubulin at 23C	1.581	0.048103657

1623383_at	CG7507-RA	Dynein heavy chain 64C	1.573	0.003554492
1636530_s_at	CG9277-RB	beta-Tubulin at 56D	1.560	0.001269578
1627638_s_at	CG5374-RB	Tcp1-like	1.532	0.002429322
1634028_s_at	CG6203-RA	CG6203 gene product from transcript CG6203-RC	1.529	0.04000817
1636809_at	CG6944-RA	Lamin	1.515	0.027962605
1638887_a_at	CG6339-RA	CG6339 gene product from transcript CG6339-RE	1.503	0.004764688
1637349_at	CG7762-RA	Regulatory particle non-ATPase 1	1.502	0.003432238
1626385_s_at	CG5643-RB	widerborst	0.663	0.001304152
1637667_at	CG17295-RA	Reduction in Cnn dots 4	0.659	0.03170289
1632648_at	CG32922-RA	CG43161 gene product from transcript CG43161-RD	0.657	0.03288023
1629227_at	CG5055-RA	bazooka	0.654	0.035730846
1629700_at	S.CX001531	maternal gene required for meiosis	0.640	0.001959656
1634962_s_at	CG9325-RB	hu li tai shao	0.638	6.58E-05
1628921_s_at	SD11801	modifier of mdg4 /// CG42769 gene product from transc	0.635	0.004457435
1628562_s_at	CG32491-RT	modifier of mdg4 /// CG42769 gene product from transc	0.632	2.57E-04
1625283_at	CG32491-RM	modifier of mdg4	0.607	0.010529318
1627605_at	CG1071-RA	E2F transcription factor 2	0.605	2.71E-04
1639308_at	CG32491-RF	modifier of mdg4	0.565	0.001798739
1632711_at	CG7864-RA	Peroxin 10	0.562	0.012550153
1632447_at	CG10851-RD	CG10851 gene product from transcript CG10851-RN	0.561	0.001826772
1629715_at	CG7891-RA	GTPase indispensable for equal segregation of chromos	0.548	4.31E-04
1631831_at	CG31040-RA	CG31040 gene product from transcript CG31040-RA	0.537	4.07E-04
1631742_s_at	CG32491-RQ	modifier of mdg4	0.537	0.00332743
1633152_at	CG32491-RA	modifier of mdg4	0.523	0.005434811
1626492_s_at	CG8978-RB	Actin-related protein 2/3 complex, subunit 1	0.517	0.002901172
1634906_at	CG4898-RC	alpha-Tubulin at 84D /// Tropomyosin 1	0.516	0.003381481
1625589_at	CG4840-RA	centrosomin's beautiful sister	0.516	4.03E-04
1638041_at	CG32491-RH	modifier of mdg4	0.516	0.012785054
1636765_at	CG32491-RJ	modifier of mdg4	0.516	0.005565568
1631082_at	CG10851-RF	CG10851 gene product from transcript CG10851-RN	0.445	0.003556395
1641021_a_at	CG6297-RB	CG6297 gene product from transcript CG6297-RC	0.442	4.49E-06
1627953_at	CG32491-RI	modifier of mdg4	0.440	0.004287487
1634052_s_at	CG32491-RA	modifier of mdg4	0.408	0.00108066
1622976_at	CG32491-RB	modifier of mdg4	0.389	0.00638984
1627483_at	CT35609	modifier of mdg4	0.375	0.003102537
1631500_at	CG32491-RR	modifier of mdg4	0.308	1.91E-04
1637164_at	CG4800-RA	Translationally controlled tumor protein	0.025	1.80E-05
**Cell differentiation**				
1632683_s_at	X02599	CG5953 gene product from transcript CG5953-RD /// ga	192.293	3.08E-07
1626324_at	CG9964-RA	CG9964 gene product from transcript CG9964-RC	35.145	0.020276947
1640377_s_at	CG2849-RB	Ras-related protein	27.693	9.37E-05
1623950_s_at	CG2198-RB	Amalgam	5.647	0.011793503
1624897_at	CG1558-RA	kinetochore Mis12-Ndc80 network component 1	4.842	0.002342818

1623109_at	CG4593-RA	CG4593 gene product from transcript CG4593-RA	4.386	1.33E-04
1628830_at	CG14121-RA	verrocchio	4.145	0.001687216
1628683_at	CG6272-RA	CG6272 gene product from transcript CG6272-RB	3.991	7.80E-05
1624815_at	CG4817-RA	Structure specific recognition protein	3.510	1.29E-04
1627246_at	CG4107-RA	Pcaf /// CG4107 gene product from transcript CG4107-R	3.486	0.001702898
1630150_s_at	CG4145-RC	Collagen type IV	3.461	0.00444349
1626392_s_at	X01472	Myocyte enhancer factor 2	3.292	2.38E-04
1631419_at	CG11129-RA	Yolk protein 3	3.262	0.004988883
1636189_at	CG12306-RA	CG12306 gene product from transcript CG12306-RA	3.172	0.002657874
1637548_at	CG18104-RA	arginase	3.113	0.018552795
1635138_at	CG10772-RA	Furin 1	2.807	0.002189608
1639036_at	CG8857-RC	Ribosomal protein S11	2.797	0.003441904
1630729_at	CG6535-RA	telomere fusion	2.788	1.16E-04
1634899_a_at	CG6512-RA	CG6512 gene product from transcript CG6512-RB	2.769	0.001523509
1639553_at	CG9987-RA	CG9987 gene product from transcript CG9987-RA	2.742	0.004720662
1628952_s_at	CG10772-RB	Furin 1	2.675	0.026843322
1640501_at	CG3829-RA	CG3829 gene product from transcript CG3829-RA	2.599	0.040440917
1627380_at	CG9633-RA	Replication Protein A 70	2.356	9.89E-05
1626425_at	CG4173-RA	Septin 2	2.331	3.96E-04
1624747_at	CG3181-RA	CG3181 gene product from transcript CG3181-RA	2.321	0.008944592
1634546_at	CG11527-RA	Tiggrin	2.285	0.011669559
1640235_at	CG5123-RA	Wrinkled	2.214	6.14E-04
1628188_at	S.C2R002789	Nucleolar protein at 60B	2.207	0.011176612
1630772_at	CG5557-RA	squeeze	2.198	0.00629058
1626774_s_at	CG13521-RA	roundabout	2.181	0.004077457
1639162_at	CG6072-RA	sarah	2.174	0.010623534
1627977_at	CG1671-RA	CG1671 gene product from transcript CG1671-RA	2.165	0.007950474
1631493_at	CG1548-RA	CG1548 gene product from transcript CG1548-RA	2.152	0.030961603
1634650_at	CG11372-RA	CG11372 gene product from transcript CG11372-RD	2.137	0.001281534
1624517_at	CG3132-RA	Ectoderm-expressed 3	2.125	0.040716585
1624686_a_at	CG32498-RO	dunce	2.086	0.004152716
1639040_s_at	CG8291-RA	bedraggled	2.085	0.031007012
1641442_a_at	CG7981-RA	terribly reduced optic lobes	2.077	0.00197086
1639414_at	CG7093-RA	Sno oncogene	2.068	0.003395452
1634351_at	CG7860-RA	CG7860 gene product from transcript CG7860-RA	2.058	0.015246837
1641267_at	CG18572-RA	rudimentary	2.048	9.03E-05
1640365_s_at	CG14021-RB	fuseless	2.047	0.010840835
1623019_a_at	CG30171-RB	CG33519 gene product from transcript CG33519-RC	2.036	0.002827574
1635692_s_at	CG9364-RE	Trehalase	2.014	2.87E-04
1632743_a_at	CG1828-RB	CG1828 gene product from transcript CG1828-RB	2.002	1.63E-04
1624138_at	CG5800-RA	CG5800 gene product from transcript CG5800-RA	1.990	4.80E-04
1634722_s_at	CG4143-RA	multiprotein bridging factor 1	1.986	5.57E-05
1627073_a_at	CG10126-RB	CG10126 gene product from transcript CG10126-RB	1.982	1.05E-04

1624668_s_at	CG9422-RA	CG9422 gene product from transcript CG9422-RC	1.978	0.006714827
1638158_at	CG4845-RA	phagocyte signaling impaired	1.971	2.08E-05
1629752_at	CG5525-RA	CG5525 gene product from transcript CG5525-RA	1.961	9.10E-05
1639335_at	GM06790	pumilio	1.941	0.001499321
1634219_a_at	CG1633-RB	thioredoxin peroxidase 1	1.935	0.005328337
1633951_at	CG7769-RA	piccolo	1.923	3.51E-04
1629229_a_at	CG3992-RA	serpent	1.922	0.024780674
1636948_a_at	CG6778-RA	Glycyl-tRNA synthetase	1.861	5.56E-04
1624807_at	CG5629-RB	Phosphopantothenoylcysteine synthetase	1.859	0.010597768
1634739_a_at	CG4001-RA	Phosphofructokinase	1.839	0.008305934
1638956_at	CG3665-RC	Fasciclin 2	1.831	0.004900134
1630324_at	CG7989-RA	wicked	1.829	5.79E-04
1636071_a_at	CG7035-RA	cap binding protein 80	1.828	1.24E-05
1637398_a_at	CG3399-RC	cappuccino	1.826	0.019995738
1632854_s_at	CG5939-RB	Paramyosin	1.825	0.048882037
1623193_at	CG33123-RA	CG33123 gene product from transcript CG33123-RA	1.819	0.001099777
1639768_at	CG2813-RA	coiled	1.816	1.81E-04
1625107_at	CG4934-RA	brainiac	1.813	0.003205006
1630408_s_at	CG7033-RC	CG7033 gene product from transcript CG7033-RA	1.811	0.002246746
1634081_at	CG6258-RA	Replication factor C 38kD subunit	1.809	0.001230874
1626150_at	CG1374-RA	teashirt	1.808	0.013230551
1624180_at	CG2222-RA	CG2222 gene product from transcript CG2222-RB	1.799	0.005006902
1636612_a_at	CG11084-RA	prickle	1.796	0.001915067
1627822_at	CG2938-RB	CG2938 gene product from transcript CG2938-RC	1.779	4.21E-04
1625038_s_at	CG10895-RA	loki	1.776	2.84E-04
1624419_a_at	CG8439-RA	T-complex Chaperonin 5	1.775	0.002378092
1632291_at	CG6625-RA	Soluble NSF attachment protein	1.762	0.005031917
1629026_at	CG3821-RA	Aspartyl-tRNA synthetase	1.760	8.69E-04
1625594_s_at	CG15862-RA	cAMP-dependent protein kinase R2	1.755	7.06E-04
1627492_at	CG5018-RA	lethal (3) 72Dn	1.751	0.005077979
1624193_s_at	CG3983-RB	nucleostemin 1	1.748	0.002982253
1636274_at	CG6531-RA	wengen	1.745	0.009502802
1626170_at	CG18445-RA	oysgedart	1.743	0.004417544
1630638_at	CG1828-RA	CG1828 gene product from transcript CG1828-RB	1.740	0.00493557
1634586_at	CG9614-RJ	pipe	1.735	0.004968485
1637847_at	CG5362-RA	Malate dehydrogenase 1	1.721	0.001571708
1630141_at	CG2158-RA	Nucleoporin 50	1.714	0.001043711
1635047_s_at	CG18214-RA	CG18214 gene product from transcript CG18214-RA	1.711	6.08E-04
1639908_a_at	CG9705-RB	CG9705 gene product from transcript CG9705-RA	1.696	1.56E-04
1635252_a_at	CG4376-RB	alpha actinin	1.692	4.58E-04
1624247_at	CG5543-RA	CG5543 gene product from transcript CG5543-RA	1.681	5.66E-04
1639118_a_at	CG12749-RA	Heterogeneous nuclear ribonucleoprotein at 87F	1.678	0.01084942
1638011_a_at	CG6148-RA	Putative Achaete Scute Target 1	1.671	4.41E-04

1632418_s_at	CG9755-RA	pumilio	1.670	0.007546981
1624779_at	CG32253-RA	CG11583 gene product from transcript CG11583-RA	1.667	0.002915495
1624774_a_at	CG3665-RB	Fasciclin 2	1.663	0.009899339
1636468_a_at	CG5033-RA	CG5033 gene product from transcript CG5033-RA	1.658	0.026317228
1626845_at	CG8351-RA	CG8351 gene product from transcript CG8351-RA	1.656	3.33E-04
1637169_at	CG12202-RA	CG12202 gene product from transcript CG12202-RA	1.649	4.39E-04
1625856_at	CG10798-RA	diminutive	1.642	0.014672076
1628665_at	CG6841-RA	CG6841 gene product from transcript CG6841-RA	1.642	5.49E-04
1637217_at	CG5714-RA	ecdysoneless	1.641	2.17E-04
1632043_a_at	CG9198-RA	shattered	1.640	0.012816613
1639766_at	CG3127-RA	Phosphoglycerate kinase	1.633	0.016749794
1640496_at	CG5722-RA	Niemann-Pick type C-1a	1.631	0.001503809
1633798_at	CG5654-RA	ypsilon schachtel	1.622	6.15E-04
1633711_a_at	CG31012-RA	CIN85 and CD2AP orthologue	1.612	0.00773198
1627916_s_at	CG1799-RC	raspberry	1.609	0.006575399
1630178_at	CG12082-RA	CG12082 gene product from transcript CG12082-RB	1.607	0.005029804
1623164_a_at	CG8127-RB	Ecdysone-induced protein 75B	1.591	0.044770837
1631973_at	CG5581-RA	Otefin	1.591	0.024740806
1640595_at	CG12301-RA	CG12301 gene product from transcript CG12301-RA	1.583	0.029998414
1623383_at	CG7507-RA	Dynein heavy chain 64C	1.573	0.003554492
1635393_s_at	GM02640	Ecdysone-induced protein 75B	1.572	0.001215019
1638070_at	CG10418-RA	CG10418 gene product from transcript CG10418-RA	1.572	0.03060154
1639736_at	CG8326-RA	CG8326 gene product from transcript CG8326-RA /// --	1.571	8.31E-04
1639554_at	CG3333-RA	Nucleolar protein at 60B	1.562	0.0418354
1625887_at	CG10732-RA	CG10732 gene product from transcript CG10732-RA	1.562	0.035024453
1627349_at	CG4539-RA	Bekka	1.558	0.022578368
1632251_s_at	CG6181-RA	CG6181 gene product from transcript CG6181-RA	1.556	1.06E-04
1624321_at	CG8556-RA	CG8556 gene product from transcript CG8556-RB	1.556	0.002188316
1623271_a_at	CG7933-RA	janus A	1.553	0.003747138
1631142_a_at	CG18026-RA	Calcium activated protein for secretion	1.544	0.041220438
1626266_at	CG10059-RA	CG10059 gene product from transcript CG10059-RA	1.539	6.59E-04
1634364_s_at	CG32130-RA	starvin	1.539	0.012907028
1625458_a_at	CG7935-RA	moleskin	1.534	0.0258634
1630344_at	CG6724-RA	CG6724 gene product from transcript CG6724-RB	1.533	0.006711513
1640145_at	CG1430-RA	by S6	1.531	0.005144843
1631223_at	CG2100-RA	CG2100 gene product from transcript CG2100-RB	1.531	0.009050993
1634028_s_at	CG6203-RA	CG6203 gene product from transcript CG6203-RC	1.529	0.04000817
1628435_at	CG16901-RA	squid	1.529	0.001872392
1634243_a_at	CG4673-RB	Npl4 ortholog	1.528	4.61E-04
1633112_at	CG4322-RA	CG4322 gene product from transcript CG4322-RD	1.524	0.024643764
1636346_at	CG7839-RA	CG7839 gene product from transcript CG7839-RA	1.523	0.028056152
1639205_s_at	CG10128-RC	transformer 2	1.523	0.001660005
1639224_at	CG9273-RA	Replication protein A2	1.522	0.001744668

1636809_at	CG6944-RA	Lamin	1.515	0.027962605
1638464_a_at	CG14039-RA	quick-to-court	1.512	0.006902332
1623402_at	CG10954-RA	Actin-related protein 2/3 complex, subunit 2	1.511	5.75E-04
1636919_at	CG9401-RA	mago nashi	1.510	0.017198913
1640907_at	CG12055-RA	Glyceraldehyde 3 phosphate dehydrogenase 1	1.509	0.002703619
1629950_at	CG1785-RA	CG1785 gene product from transcript CG1785-RA	1.508	0.035447083
1627337_at	CG11555-RA	CG11555 gene product from transcript CG11555-RA	1.507	0.00842296
1631183_at	CG9366-RA	rho-like	1.504	9.78E-04
1627796_s_at	SD05230	grappa	0.665	0.028170347
1636673_s_at	CG1856-RF	tramtrack	0.663	0.020230813
1640239_at	CG30149-RB	rigor mortis	0.660	1.46E-04
1628032_a_at	CG31092-RA	Lipophorin receptor 2	0.659	0.009983126
1629227_at	CG5055-RA	bazooka	0.654	0.035730846
1637926_s_at	CG14084-RA	CG14084 gene product from transcript CG14084-RB	0.654	9.85E-04
1629029_at	CG5160-RA	CG5160 gene product from transcript CG5160-RA	0.651	0.038203683
1630378_at	CG17657-RA	fritz	0.651	0.004134304
1630531_at	CG3421-RA	CG3421 gene product from transcript CG3421-RA	0.650	0.005021415
1631498_a_at	CG14307-RC	fruitless	0.649	0.011007547
1632207_a_at	CG1212-RA	CG1212 gene product from transcript CG1212-RD	0.640	0.017192865
1634562_s_at	CG13503-RB	Verprolin 1	0.640	0.00728029
1634962_s_at	CG9325-RB	hu li tai shao	0.638	6.58E-05
1641336_at	CG6443-RA	--- /// CG6443 gene product from transcript CG6443-RA	0.638	0.014773252
1633089_a_at	CG12052-RJ	longitudinals lacking	0.636	9.38E-04
1628921_s_at	SD11801	modifier of mdg4 /// CG42769 gene product from transc	0.635	0.004457435
1627384_at	CG6225-RA	CG6225 gene product from transcript CG6225-RB	0.635	0.001568175
1636974_at	CG32146-RA	dally-like	0.634	0.036913905
1635427_a_at	CG31043-RA	GUK-holder	0.634	7.51E-04
1630942_at	CG5742-RA	CG5742 gene product from transcript CG5742-RB	0.632	0.001485239
1628562_s_at	CG32491-RT	modifier of mdg4 /// CG42769 gene product from transc	0.632	2.57E-04
1637917_s_at	CG1856-RE	tramtrack	0.631	0.01071164
1638763_at	CG32030-RA	CG42610 gene product from transcript CG42610-RI	0.630	0.048105653
1623411_at	CG12052-RN	longitudinals lacking	0.628	0.003327923
1629270_at	CG17958-RA	Serendipity delta	0.628	0.010914986
1628778_at	CG13586-RA	ion transport peptide	0.625	0.006170257
1639083_at	CG1945-RC	fat facets	0.625	0.009429477
1640298_at	CG6308-RA	CG6308 gene product from transcript CG6308-RA	0.625	1.11E-04
1639410_at	CG7013-RA	Mesencephalic astrocyte-derived neurotrophic factor	0.621	0.004924618
1628849_at	HDC07332	CG8201 gene product from transcript CG8201-RV	0.621	0.00188239
1635754_at	CG11987-RA	tango	0.615	1.46E-04
1627041_s_at	CG15444-RB	inebriated	0.614	0.037149344
1625279_a_at	CG10619-RA	tailup	0.612	0.03589902
1631242_at	CG4029-RA	jumeau	0.610	0.016876327
1625283_at	CG32491-RM	modifier of mdg4	0.607	0.010529318

1638466_a_at	CG4370-RA	Inwardly rectifying potassium channel 2	0.606	0.01349143
1627605_at	CG1071-RA	E2F transcription factor 2	0.605	2.71E-04
1631340_at	CG8261-RA	G protein gamma 1	0.604	0.01811019
1631408_at	CG18024-RA	SoxNeuro	0.594	6.84E-04
1628336_a_at	CG12369-RB	Lachesin	0.594	0.008119137
1639139_a_at	CG8874-RB	Fps oncogene analog	0.593	0.002128981
1631207_at	CG4220-RA	elbow B	0.590	0.00514124
1630456_at	CG32156-RE	Myosin binding subunit	0.590	0.001187458
1626352_at	GM04742	scribbler	0.587	0.004717244
1626158_s_at	CG9655-RA	nessy	0.578	0.001264002
1625048_at	CG8318-RA	Neurofibromin 1	0.578	0.030962145
1631674_at	CG12366-RA	O-fucosyltransferase 1	0.573	0.014372858
1631527_at	CG5642-RA	CG5642 gene product from transcript CG5642-RA	0.573	7.52E-04
1640313_s_at	CG6202-RA	Surfeit 4	0.569	0.002513118
1639308_at	CG32491-RF	modifier of mdg4	0.565	0.001798739
1641448_a_at	CG18250-RA	Dystroglycan	0.562	0.004142304
1632711_at	CG7864-RA	Peroxin 10	0.562	0.012550153
1634063_a_at	CG31317-RA	CG31317 gene product from transcript CG31317-RC	0.559	0.04519155
1635314_at	CG9954-RA	CG9954 gene product from transcript CG9954-RA	0.547	6.33E-04
1641068_a_at	CG6134-RA	spatzle	0.545	0.023638569
1637703_a_at	CG15154-RA	Suppressor of cytokine signaling at 36E	0.542	1.35E-05
1631742_s_at	CG32491-RQ	modifier of mdg4	0.537	0.00332743
1625542_a_at	CG4898-RE	Tropomyosin 1	0.536	0.001913851
1635737_at	CG6701-RA	CG6701 gene product from transcript CG6701-RD	0.527	0.001728137
1638875_at	CG6542-RA	Egg-derived tyrosine phosphatase	0.525	0.008692758
1632508_s_at	CG11282-RA	capricious	0.525	0.008637066
1626165_at	CG3754-RA	Phosphodiesterase 9	0.524	0.002821438
1637161_s_at	CG1838-RA	myoglianin	0.523	0.006115961
1633152_at	CG32491-RA	modifier of mdg4	0.523	0.005434811
1629129_at	CG7525-RA	Tie-like receptor tyrosine kinase	0.519	3.18E-04
1626492_s_at	CG8978-RB	Actin-related protein 2/3 complex, subunit 1	0.517	0.002901172
1638041_at	CG32491-RH	modifier of mdg4	0.516	0.012785054
1636765_at	CG32491-RJ	modifier of mdg4	0.516	0.005565568
1630841_at	CG8668-RA	CG8668 gene product from transcript CG8668-RA	0.509	0.002753527
1633384_at	CG9250-RA	M-phase phosphoprotein 6	0.509	1.93E-04
1632381_at	CG14226-RA	domeless	0.506	0.026396735
1623877_a_at	CG4220-RA	elbow B	0.498	0.023710683
1638580_at	S.C3L001717	capricious	0.479	0.020279841
1626461_at	CG4357-RA	Heat shock protein 60 related /// sodium chloride cotran	0.477	0.001494621
1636606_at	CG7623-RA	slalom	0.470	0.007295398
1641365_s_at	SD27140	CG11352 gene product from transcript CG11352-RC	0.464	1.94E-05
1628604_at	CG5893-RA	Dichaete	0.458	0.005151275
1637478_s_at	CG31000-RB	hephaestus	0.448	0.015236953

1641021_a_at	CG6297-RB	CG6297 gene product from transcript CG6297-RC	0.442	4.49E-06
1627953_at	CG32491-RI	modifier of mdg4	0.440	0.004287487
1626624_s_at	CG31794-RC	Paxillin	0.437	0.001420985
1624533_s_at	CG8651-RA	trithorax	0.427	2.59E-04
1635910_at	CG7331-RA	Autophagy-specific gene 13	0.423	0.010410408
1638821_a_at	CG31732-RA	yuri gagarin	0.419	0.001551989
1634052_s_at	CG32491-RA	modifier of mdg4	0.408	0.00108066
1639568_at	CG31285-RA	liquid facets-Related	0.407	7.79E-05
1640867_s_at	CG7223-RB	heartless	0.395	0.004595591
1622976_at	CG32491-RB	modifier of mdg4	0.389	0.00638984
1634920_at	LD07388	capricious	0.385	0.008747041
1627483_at	CT35609	modifier of mdg4	0.375	0.003102537
1638807_s_at	CG4829-RB	CG4829 gene product from transcript CG4829-RB	0.358	1.22E-04
1628458_at	CG13777-RC	milton	0.324	0.004074056
1625276_a_at	CG7266-RC	Ecdysone-induced protein 28/29kD	0.312	0.00480965
1631500_at	CG32491-RR	modifier of mdg4	0.308	1.91E-04
1623807_a_at	CG13777-RA	milton	0.294	7.27E-04
1632404_at	CG5760-RA	tetracycline resistance	0.246	9.47E-05
1632715_at	RH40482	Tie-like receptor tyrosine kinase	0.220	2.80E-04
1635725_a_at	CG31332-RC	CG31352 gene product from transcript CG31352-RA ///	0.216	9.19E-04
1629713_at	CG9187-RA	CG9187 gene product from transcript CG9187-RA	0.211	6.37E-06
**DNA Repair**				
1640431_at	CG5247-RA	Inverted repeat-binding protein (ku70)	5.422	1.18E-06
1625109_at	CG3178-RA	Recombination repair protein 1	3.720	1.61E-04
1625759_at	CG18801-RA	CG18801 gene product from transcript CG18801-RA (k	3.663	7.89E-04
1624815_at	CG4817-RA	Structure specific recognition protein	3.510	1.29E-04
1635299_at	CG12189-RA	CG12189 gene product from transcript CG12189-RA	3.179	1.84E-05
1630729_at	CG6535-RA	telomere fusion	2.788	1.16E-04
1624269_at	CG8825-RA	glaikit	2.473	8.23E-04
1627380_at	CG9633-RA	Replication Protein A 70	2.356	9.89E-05
1636131_at	CG17227-RA	DNA ligase III	2.156	6.20E-04
1639904_at	CG1303-RA	O-6-alkylguanine-DNA alkyltransferase	2.148	0.019304246
1632743_a_at	CG1828-RB	CG1828 gene product from transcript CG1828-RB	2.002	1.63E-04
1633951_at	CG7769-RA	piccolo	1.923	3.51E-04
1625500_at	CG11482-RA	CG11482 gene product from transcript CG11482-RB	1.884	0.004672722
1630390_at	CG5602-RA	DNA ligase I	1.880	0.00708321
1623485_at	CG6358-RA	Xeroderma pigmentosum group A-like	1.828	0.002477636
1630638_at	CG1828-RA	CG1828 gene product from transcript CG1828-RB	1.740	0.00493557
1633353_s_at	CG18408-RI	CG18408 gene product from transcript CG18408-RI	1.649	0.022984264
1632827_a_at	CG8153-RB	mutagen-sensitive 210	1.561	0.003923746
1626266_at	CG10059-RA	CG10059 gene product from transcript CG10059-RA	1.539	6.59E-04
1626850_s_at	CG31953-RA	glaikit	1.527	0.015734637
1639224_at	CG9273-RA	Replication protein A2	1.522	0.001744668

1638887_a_at	CG6339-RA	CG6339 gene product from transcript CG6339-RE	1.503	0.004764688
1638161_at	CG11115-RA	CG11115 gene product from transcript CG11115-RA	0.653	0.018895539
1633957_s_at	CG5316-RC	CG5316 gene product from transcript CG5316-RC	0.435	3.64E-04
1636758_s_at	CG11205-RA	CG18853 gene product from transcript CG18853-RA ///	0.047	6.25E-05
1637164_at	CG4800-RA	Translationally controlled tumor protein	0.025	1.80E-05
**Extracellular matrix**				
1638869_at	CG10112-RA	Cuticular protein 51A	8.771	5.60E-04
1630150_s_at	CG4145-RC	Collagen type IV	3.461	0.00444349
1625825_at	CG6378-RA	CG6378 gene product from transcript CG6378-RA	2.962	0.008401127
1634546_at	CG11527-RA	Tiggrin	2.285	0.011669559
1631701_a_at	CG8502-RC	Cuticular protein 49Ac	2.220	0.03761458
1641442_a_at	CG7981-RA	terribly reduced optic lobes	2.077	0.00197086
1639229_at	CG16858-RA	viking	1.911	0.007589053
1636974_at	CG32146-RA	dally-like	0.634	0.036913905
1624283_at	CG13224-RA	Cuticular protein 47Eb	0.628	0.014264812
1640097_at	CG12002-RA	Peroxidasin	0.437	0.03795444
1637562_at	CG5812-RA	TweedleT	0.282	0.009158006
**Immune response**				
1640377_s_at	CG2849-RB	Ras-related protein	27.693	9.37E-05
1626392_s_at	X01472	Myocyte enhancer factor 2	3.292	2.38E-04
1635989_at	CG10658-RA	Helical Factor	3.255	0.022472996
1641623_at	CG15917-RA	Growth-blocking peptide	3.036	0.020328043
1626272_s_at	CG3066-RA	Serine protease 7	2.438	0.015278731
1631701_a_at	CG8502-RC	Cuticular protein 49Ac	2.220	0.03761458
1639526_a_at	CG7717-RA	CG7717 gene product from transcript CG7717-RB	2.109	0.003677665
1638158_at	CG4845-RA	phagocyte signaling impaired	1.971	2.08E-05
1636266_at	CG6367-RA	persephone	1.936	0.018853232
1626473_a_at	CG15825-RB	fondue	1.896	0.048782084
1634848_at	CG16705-RA	Spatzle-Processing Enzyme	1.888	0.04697614
1641419_at	CG4740-RA	Attacin-C	1.885	0.017400064
1623164_a_at	CG8127-RB	Ecdysone-induced protein 75B	1.591	0.044770837
1623383_at	CG7507-RA	Dynein heavy chain 64C	1.573	0.003554492
1635393_s_at	GM02640	Ecdysone-induced protein 75B	1.572	0.001215019
1624321_at	CG8556-RA	CG8556 gene product from transcript CG8556-RB	1.556	0.002188316
1625923_s_at	CG32593-RA	Flotillin-2	1.538	0.001167682
1637327_at	CG11376-RA	Zizimin-related	1.526	0.006760646
1631183_at	CG9366-RA	rho-like	1.504	9.78E-04
1637332_at	CG5576-RA	immune deficiency	0.664	3.38E-04
1633089_a_at	CG12052-RJ	longitudinals lacking	0.636	9.38E-04
1623411_at	CG12052-RN	longitudinals lacking	0.628	0.003327923
1628849_at	HDC07332	CG8201 gene product from transcript CG8201-RV	0.621	0.00188239
1641458_s_at	CG1511-RD	Eph receptor tyrosine kinase	0.601	3.16E-04
1639139_a_at	CG8874-RB	Fps oncogene analog	0.593	0.002128981

1641068_a_at	CG6134-RA	spatzle	0.545	0.023638569
1632381_at	CG14226-RA	domeless	0.506	0.026396735
1640654_at	CG31770-RB	Hemese	0.396	0.027511295

Locomotion

1640377_s_at	CG2849-RB	Ras-related protein	27.693	9.37E-05
1634546_at	CG11527-RA	Tiggrin	2.285	0.011669559
1640235_at	CG5123-RA	Wrinkled	2.214	6.14E-04
1630772_at	CG5557-RA	squeeze	2.198	0.00629058
1626774_s_at	CG13521-RA	roundabout	2.181	0.004077457
1641442_a_at	CG7981-RA	terribly reduced optic lobes	2.077	0.00197086
1641267_at	CG18572-RA	rudimentary	2.048	9.03E-05
1638158_at	CG4845-RA	phagocyte signaling impaired	1.971	2.08E-05
1639335_at	GM06790	pumilio	1.941	0.001499321
1634219_a_at	CG1633-RB	thioredoxin peroxidase 1	1.935	0.005328337
1629229_a_at	CG3992-RA	serpent	1.922	0.024780674
1624807_at	CG5629-RB	Phosphopantothenoylcysteine synthetase	1.859	0.010597768
1638956_at	CG3665-RC	Fasciclin 2	1.831	0.004900134
1625107_at	CG4934-RA	brainiac	1.813	0.003205006
1635021_at	CG2019-RA	dispatched	1.779	0.02253891
1638469_s_at	AY180918	CG3857 gene product from transcript CG3857-RA	1.778	6.47E-04
1625594_s_at	CG15862-RA	cAMP-dependent protein kinase R2	1.755	7.06E-04
1636274_at	CG6531-RA	wengen	1.745	0.009502802
1626170_at	CG18445-RA	oysgedart	1.743	0.004417544
1635047_s_at	CG18214-RA	CG18214 gene product from transcript CG18214-RA	1.711	6.08E-04
1632418_s_at	CG9755-RA	pumilio	1.670	0.007546981
1624774_a_at	CG3665-RB	Fasciclin 2	1.663	0.009899339
1630941_s_at	CG16944-RA	stress-sensitive B	1.613	0.001486399
1633711_a_at	CG31012-RA	CIN85 and CD2AP orthologue	1.612	0.00773198
1627916_s_at	CG1799-RC	raspberry	1.609	0.006575399
1623383_at	CG7507-RA	Dynein heavy chain 64C	1.573	0.003554492
1624321_at	CG8556-RA	CG8556 gene product from transcript CG8556-RB	1.556	0.002188316
1631142_a_at	CG18026-RA	Calcium activated protein for secretion	1.544	0.041220438
1634028_s_at	CG6203-RA	CG6203 gene product from transcript CG6203-RC	1.529	0.04000817
1628435_at	CG16901-RA	squid	1.529	0.001872392
1631183_at	CG9366-RA	rho-like	1.504	9.78E-04
1636673_s_at	CG1856-RF	tramtrack	0.663	0.020230813
1629227_at	CG5055-RA	bazooka	0.654	0.035730846
1630531_at	CG3421-RA	CG3421 gene product from transcript CG3421-RA	0.650	0.005021415
1631498_a_at	CG14307-RC	fruitless	0.649	0.011007547
1634962_s_at	CG9325-RB	hu li tai shao	0.638	6.58E-05
1633089_a_at	CG12052-RJ	longitudinals lacking	0.636	9.38E-04
1628921_s_at	SD11801	modifier of mdg4 /// CG42769 gene product from transc	0.635	0.004457435
1636974_at	CG32146-RA	dally-like	0.634	0.036913905
1635427_a_at	CG31043-RA	GUK-holder	0.634	7.51E-04
1628562_s_at	CG32491-RT	modifier of mdg4 /// CG42769 gene product from transc	0.632	2.57E-04
1637917_s_at	CG1856-RE	tramtrack	0.631	0.01071164
1638763_at	CG32030-RA	CG42610 gene product from transcript CG42610-RI	0.630	0.048105653
1623411_at	CG12052-RN	longitudinals lacking	0.628	0.003327923
1639083_at	CG1945-RC	fat facets	0.625	0.009429477
1628849_at	HDC07332	CG8201 gene product from transcript CG8201-RV	0.621	0.00188239
1635754_at	CG11987-RA	tango	0.615	1.46E-04
1625279_a_at	CG10619-RA	tailup	0.612	0.03589902
1625283_at	CG32491-RM	modifier of mdg4	0.607	0.010529318
1631408_at	CG18024-RA	SoxNeuro	0.594	6.84E-04
1639139_a_at	CG8874-RB	Fps oncogene analog	0.593	0.002128981
1626352_at	GM04742	scribbler	0.587	0.004717244
1626158_s_at	CG9655-RA	nessy	0.578	0.001264002
1639308_at	CG32491-RF	modifier of mdg4	0.565	0.001798739
1641448_a_at	CG18250-RA	Dystroglycan	0.562	0.004142304
1632770_at	CR_tc_GM107	like-AP180	0.562	0.006977987
1634063_a_at	CG31317-RA	CG31317 gene product from transcript CG31317-RC	0.559	0.04519155
1641068_a_at	CG6134-RA	spatzle	0.545	0.023638569
1637703_a_at	CG15154-RA	Suppressor of cytokine signaling at 36E	0.542	1.35E-05
1631742_s_at	CG32491-RQ	modifier of mdg4	0.537	0.00332743
1632508_s_at	CG11282-RA	capricious	0.525	0.008637066
1633152_at	CG32491-RA	modifier of mdg4	0.523	0.005434811
1629129_at	CG7525-RA	Tie-like receptor tyrosine kinase	0.519	3.18E-04
1638041_at	CG32491-RH	modifier of mdg4	0.516	0.012785054
1636765_at	CG32491-RJ	modifier of mdg4	0.516	0.005565568
1632381_at	CG14226-RA	domeless	0.506	0.026396735
1638580_at	S.C3L001717	capricious	0.479	0.020279841
1628604_at	CG5893-RA	Dichaete	0.458	0.005151275
1627953_at	CG32491-RI	modifier of mdg4	0.440	0.004287487
1624533_s_at	CG8651-RA	trithorax	0.427	2.59E-04
1638821_a_at	CG31732-RA	yuri gagarin	0.419	0.001551989
1624577_at	CG17907-RA	Acetylcholine esterase	0.414	6.04E-04
1634052_s_at	CG32491-RA	modifier of mdg4	0.408	0.00108066
1640867_s_at	CG7223-RB	heartless	0.395	0.004595591
1622976_at	CG32491-RB	modifier of mdg4	0.389	0.00638984
1634920_at	LD07388	capricious	0.385	0.008747041
1627483_at	CT35609	modifier of mdg4	0.375	0.003102537
1631500_at	CG32491-RR	modifier of mdg4	0.308	1.91E-04
1632715_at	RH40482	Tie-like receptor tyrosine kinase	0.220	2.80E-04
1635725_a_at	CG31332-RC	CG31352 gene product from transcript CG31352-RA ///	0.216	9.19E-04
1626797_a_at	CG7075-RA	Neurotransmitter transporter-like	0.028	0.00108647

Metabolic process

1636767_at	CG9116-RA	Lysozyme P	230.523	2.30E-06
1635512_at	CG11893-RA	CG11893 gene product from transcript CG11893-RA	228.237	6.46E-04
1640590_at	CG32195-RA	CG32195 gene product from transcript CG32195-RA	43.210	0.008713688
1626324_at	CG9964-RA	CG9964 gene product from transcript CG9964-RC	35.145	0.020276947
1623486_at	CG7900-RA	CG7900 gene product from transcript CG7900-RB	31.138	5.06E-06
1640377_s_at	CG2849-RB	Ras-related protein	27.693	9.37E-05
1638182_at	CG5999-RA	CG5999 gene product from transcript CG5999-RB	16.425	0.004394127
1625141_at	CG14715-RA	CG14715 gene product from transcript CG14715-RA	15.822	1.40E-04
1626984_at	CG1152-RA	Glucose dehydrogenase	15.261	1.17E-05
1628963_at	CG4716-RA	CG4716 gene product from transcript CG4716-RA /// C	11.931	0.015780449
1641412_at	CG31809-RA	CG31809 gene product from transcript CG31809-RB	10.237	1.75E-05
1624658_at	CG33158-RB	CG33158 gene product from transcript CG33158-RB	9.585	0.006631239
1623256_at	CG5164-RA	Glutathione S transferase E1	9.331	0.001191725
1625744_at	CG17530-RA	Glutathione S transferase E6	9.218	3.55E-05
1641428_at	CG3616-RA	Cytochrome P450-9c1	9.108	1.04E-04
1639495_at	CG4485-RA	Cytochrome P450-9b1	8.273	0.00499798
1637872_at	CG3926-RA	Serine pyruvate aminotransferase	8.153	0.00394151
1629046_a_at	CG4716-RB	CG4716 gene product from transcript CG4716-RA /// C	7.462	0.020129774
1622946_at	CG6908-RA	CG6908 gene product from transcript CG6908-RA	6.812	0.006671085
1627430_at	CG8888-RA	CG8888 gene product from transcript CG8888-RA	6.635	2.36E-04
1635318_at	CG8721-RA	Ornithine decarboxylase 1	6.405	0.027647763
1638013_at	CG30289-RA	CG30289 gene product from transcript CG30289-RA	5.750	0.003036632
1633622_at	CG3008-RA	CG3008 gene product from transcript CG3008-RA	5.678	3.72E-04
1640431_at	CG5247-RA	Inverted repeat-binding protein	5.422	1.18E-06
1640065_at	CG17531-RA	Glutathione S transferase E7	5.226	0.00853174
1632676_s_at	CG11897-RA	CG11897 gene product from transcript CG11897-RB	5.166	3.38E-04
1633284_at	CG17611-RA	CG17611 gene product from transcript CG17611-RA	4.789	0.003144433
1626446_a_at	CG5596-RA	Myosin alkali light chain 1	4.772	2.76E-04
1641174_at	CG7361-RA	Rieske iron-sulfur protein	4.671	0.00357398
1633503_at	CG12171-RA	CG12171 gene product from transcript CG12171-RA	4.530	0.001939612
1635593_at	CG4373-RA	CG4373 gene product from transcript CG4373-RA	4.527	6.36E-05
1634794_at	CG10924-RA	CG10924 gene product from transcript CG10924-RA ///	4.499	0.019180775
1626248_at	CG18547-RA	CG18547 gene product from transcript CG18547-RA	4.495	0.017862516
1627516_at	CG6834-RA	CG6834 gene product from transcript CG6834-RA	4.471	0.010012346
1625139_at	CG11086-RA	CG11086 gene product from transcript CG11086-RA	4.360	0.012115425
1640488_a_at	CG33085-RA	CG9510 gene product from transcript CG9510-RC /// C	4.245	0.013808396
1623782_at	CG33131-RA	SREBP cleavage activating protein	4.237	9.33E-05
1628830_at	CG14121-RA	verrocchio	4.145	0.001687216
1626801_at	CG10638-RA	CG10638 gene product from transcript CG10638-RA	4.041	1.33E-04
1630430_a_at	CG30437-RA	laccase 2	3.975	0.002402744
1623569_at	CG14907-RA	CG14906 gene product from transcript CG14906-RC ///	3.791	9.95E-06
1625109_at	CG3178-RA	Recombination repair protein 1	3.720	1.61E-04
1640379_s_at	CG11079-RA	CG34424 gene product from transcript CG34424-RA	3.715	0.009987739

1625759_at	CG18801-RA	CG18801 gene product from transcript CG18801-RA	3.663	7.89E-04
1634699_a_at	CG30019-RB	CG18003 gene product from transcript CG18003-RA ///	3.662	0.007402957
1632432_at	CG9850-RA	CG9850 gene product from transcript CG9850-RA	3.651	0.001734573
1631628_s_at	CG11765-RA	CG12896 gene product from transcript CG12896-RA //	3.615	0.004637056
1635610_at	CG8310-RA	Vacuolar H[+] ATPase subunit 36-3	3.595	1.61E-04
1629182_at	CG10140-RA	CG10140 gene product from transcript CG10140-RA	3.527	0.00243882
1624815_at	CG4817-RA	Structure specific recognition protein	3.510	1.29E-04
1628942_at	CG15385-RA	CG15385 gene product from transcript CG15385-RA	3.501	0.026401859
1639415_at	CG4104-RA	Trehalose-6-phosphate synthase 1	3.499	0.029702358
1627246_at	CG4107-RA	Pcaf /// CG4107 gene product from transcript CG4107-R	3.486	0.001702898
1637444_s_at	CG4067-RB	pugilist	3.473	0.012210707
1633599_a_at	CG17725-RB	Phosphoenolpyruvate carboxykinase	3.463	0.009546949
1637037_at	CG31628-RA	adenosine 3	3.435	0.02835378
1630802_at	CG12800-RA	CG12800 gene product from transcript CG12800-RA	3.394	0.004700292
1636689_at	CG4259-RA	CG4259 gene product from transcript CG4259-RB	3.364	0.048401404
1635110_at	CG2397-RA	CG2397 gene product from transcript CG2397-RA	3.362	0.012078666
1637461_at	CG9527-RA	CG9527 gene product from transcript CG9527-RC	3.310	0.016161317
1636258_at	CG8550-RA	CG8550 gene product from transcript CG8550-RA	3.298	0.01604587
1626392_s_at	X01472	Myocyte enhancer factor 2	3.292	2.38E-04
1631419_at	CG11129-RA	Yolk protein 3	3.262	0.004988883
1625042_at	CG31288-RA	CG31288 gene product from transcript CG31288-RA	3.231	0.006933038
1639482_a_at	CG32549-RD	CG32549 gene product from transcript CG32549-RD	3.195	3.02E-04
1635299_at	CG12189-RA	CG12189 gene product from transcript CG12189-RA	3.179	1.84E-05
1636189_at	CG12306-RA	CG12306 gene product from transcript CG12306-RA	3.172	0.002657874
1626503_at	CG2254-RA	CG2254 gene product from transcript CG2254-RA /// --	3.150	0.03775991
1637548_at	CG18104-RA	arginase	3.113	0.018552795
1641623_at	CG15917-RA	Growth-blocking peptide	3.036	0.020328043
1634583_s_at	CG4822-RA	CG4822 gene product from transcript CG4822-RE	3.032	0.006308328
1626086_at	CG10621-RA	CG10621 gene product from transcript CG10621-RA	2.987	0.03463095
1633471_at	CG11765-RA	Peroxiredoxin 2540-2	2.934	0.017057572
1629716_a_at	CG13284-RA	CG13284 gene product from transcript CG13284-RD	2.897	6.75E-04
1625362_at	CG2259-RA	Glutamate-cysteine ligase catalytic subunit	2.896	0.00306305
1639535_at	CG3254-RA	polypeptide GalNAc transferase 2	2.883	6.92E-04
1623151_a_at	CG2010-RB	CG2010 gene product from transcript CG2010-RB	2.863	1.55E-04
1627719_at	CG2679-RA	goliath	2.809	6.30E-04
1635138_at	CG10772-RA	Furin 1	2.807	0.002189608
1639036_at	CG8857-RC	Ribosomal protein S11	2.797	0.003441904
1630729_at	CG6535-RA	telomere fusion	2.788	1.16E-04
1625697_at	CG8453-RA	CG8453 gene product from transcript CG8453-RA	2.786	0.046594556
1634899_a_at	CG6512-RA	CG6512 gene product from transcript CG6512-RB	2.769	0.001523509
1633654_s_at	CG1630-RA	Inositol 1,4,5-triphosphate kinase 2	2.768	4.82E-04
1629705_at	CG6084-RB	CG6084 gene product from transcript CG6084-RC	2.750	0.002387339
1639553_at	CG9987-RA	CG9987 gene product from transcript CG9987-RA	2.742	0.004720662

1641455_at	CG6762-RA	CG6762 gene product from transcript CG6762-RD	2.742	0.001599772
1628935_at	CG10962-RB	CG10962 gene product from transcript CG10962-RB	2.721	7.91E-05
1637806_at	CG11994-RA	Adenosine deaminase	2.700	1.80E-05
1628952_s_at	CG10772-RB	Furin 1	2.675	0.026843322
1636762_a_at	CG9148-RA	supercoiling factor	2.670	1.57E-04
1638804_a_at	CG40293-RB	Ste20-like kinase	2.622	0.00200459
1623900_a_at	CG14935-RA	Maltase B2	2.610	0.01989313
1627013_at	CG1597-RA	CG1597 gene product from transcript CG1597-RB	2.592	3.03E-05
1639480_at	CG6871-RA	Catalase	2.592	0.009853024
1631977_at	CG31014-RA	prolyl-4-hydroxylase-alpha SG1	2.545	1.25E-04
1629194_s_at	CG9127-RA	adenosine 2	2.542	0.009325657
1637000_at	CG12403-RA	Vacuolar H[+] ATPase subunit 68-1	2.540	0.003899206
1640918_at	CG32145-RA	omega	2.535	9.28E-05
1632852_s_at	CG2718-RB	Glutamine synthetase 1	2.527	0.001908985
1640035_at	CG3763-RA	Fat body protein 2	2.519	0.046472386
1626136_at	CG4423-RA	Glutathione S transferase D6	2.517	0.042772077
1637489_at	CG6133-RA	NOP2-Sun domain family, member 2 ortholog	2.509	8.11E-04
1631258_at	CG30005-RA	Glutathione S transferase T2	2.477	3.42E-05
1624269_at	CG8825-RA	glaikit	2.473	8.23E-04
1637109_s_at	CG3961-RA	CG3961 gene product from transcript CG3961-RC	2.455	0.032773342
1625665_at	CG17166-RA	mitochondrial ribosomal protein L39	2.445	1.07E-05
1627890_at	CG18548-RA	Glutathione S transferase D10	2.445	0.002680001
1626272_s_at	CG3066-RA	Serine protease 7	2.438	0.015278731
1633059_at	CG6357-RA	CG6357 gene product from transcript CG6357-RA	2.427	0.019551273
1629309_at	CG7627-RA	CG7627 gene product from transcript CG7627-RB	2.420	5.56E-04
1625652_s_at	CG12428-RB	CG12428 gene product from transcript CG12428-RB	2.418	0.02330595
1632812_at	CG9504-RA	Ecdysone oxidase	2.417	4.54E-04
1630258_at	CG4181-RA	Glutathione S transferase D2	2.400	0.010337692
1631439_at	CG8719-RA	Ornithine decarboxylase 2	2.398	0.02337745
1632114_at	CG6042-RA	CG6042 gene product from transcript CG6042-RA	2.378	0.010436881
1640521_at	CG6361-RA	CG6361 gene product from transcript CG6361-RB /// H	2.372	0.042985193
1627380_at	CG9633-RA	Replication Protein A 70	2.356	9.89E-05
1634838_at	CG32101-RA	CG10638 gene product from transcript CG10638-RA	2.341	0.01753851
1639677_at	CG9952-RA	partner of paired	2.332	7.21E-04
1626425_at	CG4173-RA	Septin 2	2.331	3.96E-04
1633401_s_at	CG30489-RA	CG33503 gene product from transcript CG33503-RA //	2.331	0.019439487
1639971_at	CG12224-RA	CG12224 gene product from transcript CG12224-RC	2.323	0.004564534
1624747_at	CG3181-RA	CG3181 gene product from transcript CG3181-RA	2.321	0.008944592
1639643_at	CG18557-RA	CG18557 gene product from transcript CG18557-RA	2.315	0.00116812
1631392_at	CG11089-RA	CG11089 gene product from transcript CG11089-RD ///	2.298	0.042752024
1631333_s_at	CG32954-RB	Alcohol dehydrogenase /// Adh-related	2.297	0.041134134
1630619_at	CG9456-RA	Serpin 42Dd	2.287	0.009781473
1623508_at	CG30466-RA	CG30466 gene product from transcript CG30466-RA	2.287	0.006566299

1634995_at	CG3788-RA	CG3788 gene product from transcript CG3788-RB	2.283	9.62E-04
1633292_a_at	CG4300-RA	CG4300 gene product from transcript CG4300-RB	2.276	6.84E-04
1630212_at	CG2065-RA	CG2065 gene product from transcript CG2065-RB	2.272	0.004447056
1629413_at	CG12703-RA	CG12703 gene product from transcript CG12703-RD	2.267	3.62E-06
1640599_at	CG3989-RA	CG3989 gene product from transcript CG3989-RB	2.235	0.03976985
1633881_at	CG1776-RA	spaghetti-squash activator	2.221	0.004615664
1640202_at	CG4475-RA	Imaginal disc growth factor 2	2.219	0.03824646
1628896_a_at	CG4210-RB	CG4210 gene product from transcript CG4210-RA	2.216	0.00371318
1640235_at	CG5123-RA	Wrinkled	2.214	6.14E-04
1636219_at	CG9210-RA	Adenylyl cyclase 35C	2.212	0.00149562
1628188_at	S.C2R002789	Nucleolar protein at 60B	2.207	0.011176612
1636374_at	CG7820-RA	Carbonic anhydrase 1	2.203	0.001617437
1630772_at	CG5557-RA	squeeze	2.198	0.00629058
1640977_at	CG12264-RA	CG12264 gene product from transcript CG12264-RA	2.191	6.17E-04
1641576_a_at	CG5205-RB	CG5205 gene product from transcript CG5205-RA	2.185	2.02E-04
1634412_at	CG2135-RA	CG2135 gene product from transcript CG2135-RA	2.184	0.002250753
1633391_at	CG8913-RA	Immune-regulated catalase	2.184	0.04989941
1627977_at	CG1671-RA	CG1671 gene product from transcript CG1671-RA	2.165	0.007950474
1628807_at	CG5205-RA	CG5205 gene product from transcript CG5205-RA	2.157	4.47E-05
1636131_at	CG17227-RA	DNA ligase III	2.156	6.20E-04
1631493_at	CG1548-RA	CG1548 gene product from transcript CG1548-RA	2.152	0.030961603
1639904_at	CG1303-RA	O-6-alkylguanine-DNA alkyltransferase	2.148	0.019304246
1636353_s_at	CG8112-RA	CG8112 gene product from transcript CG8112-RA	2.147	0.015056766
1624517_at	CG3132-RA	Ectoderm-expressed 3	2.125	0.040716585
1639526_a_at	CG7717-RA	CG7717 gene product from transcript CG7717-RB	2.109	0.003677665
1638844_s_at	CG3714-RC	CG3714 gene product from transcript CG3714-RG	2.107	4.67E-04
1631513_at	CG30000-RA	Glutathione S transferase T1	2.090	0.002291358
1622908_a_at	CG10962-RA	CG10962 gene product from transcript CG10962-RB ///	2.089	2.87E-07
1624686_a_at	CG32498-RO	dunce	2.086	0.004152716
1635768_at	CG4670-RA	CG4670 gene product from transcript CG4670-RA	2.076	0.044413283
1623459_at	CG9453-RC	Serpin 42Da	2.076	1.13E-04
1627314_s_at	CG5202-RA	CG5202 gene product from transcript CG5202-RA /// Q	2.074	9.88E-04
1628705_at	CG31022-RA	prolyl-4-hydroxylase-alpha EFB	2.063	0.005935237
1624663_a_at	CG8821-RA	vismay	2.063	0.004561812
1634351_at	CG7860-RA	CG7860 gene product from transcript CG7860-RA	2.058	0.015246837
1627912_at	CG5493-RA	CG5493 gene product from transcript CG5493-RB	2.055	0.04634876
1636410_at	CG3505-RA	CG3505 gene product from transcript CG3505-RA	2.051	0.03560616
1641267_at	CG18572-RA	rudimentary	2.048	9.03E-05
1624846_at	CG10764-RA	CG10764 gene product from transcript CG10764-RA	2.047	0.013922697
1623019_a_at	CG30171-RB	CG33519 gene product from transcript CG33519-RC	2.036	0.002827574
1636943_s_at	CG18525-RA	Serpin 88Ea	2.036	0.001654406
1627946_at	CG12068-RA	shroud	2.026	7.05E-04
1635692_s_at	CG9364-RE	Trehalase	2.014	2.87E-04

1631277_at	CG1082-RA	alpha-Esterase-4	2.011	0.02581075
1632743_a_at	CG1828-RB	CG1828 gene product from transcript CG1828-RB	2.002	1.63E-04
1627572_at	CG31219-RA	CG31219 gene product from transcript CG31219-RA	1.998	0.005547678
1632667_s_at	CG8893-RB	Glyceraldehyde 3 phosphate dehydrogenase 2	1.990	0.001078783
1624138_at	CG5800-RA	CG5800 gene product from transcript CG5800-RA	1.990	4.80E-04
1629569_at	CG1839-RA	F box and leucine-rich-repeat gene 4	1.984	1.06E-04
1640552_at	CG4919-RA	Glutamate-cysteine ligase modifier subunit	1.980	0.03054508
1635008_at	CG4486-RA	Cytochrome P450-9b2	1.976	0.023370435
1631388_at	CG8432-RA	Rab escort protein	1.972	3.67E-04
1639621_at	CG10916-RA	CG10916 gene product from transcript CG10916-RB	1.967	2.18E-04
1629752_at	CG5525-RA	CG5525 gene product from transcript CG5525-RA	1.961	9.10E-05
1627253_at	CG33122-RA	CG33122 gene product from transcript CG33122-RA	1.959	0.00836865
1623931_s_at	CG9819-RA	Calcineurin A at 14F	1.957	0.016841503
1626326_at	CG31436-RA	CG31436 gene product from transcript CG31436-RA	1.946	1.97E-05
1630092_at	CG13088-RA	CG13088 gene product from transcript CG13088-RA	1.941	3.36E-04
1639335_at	GM06790	pumilio	1.941	0.001499321
1636266_at	CG6367-RA	persephone	1.936	0.018853232
1634219_a_at	CG1633-RB	thioredoxin peroxidase 1	1.935	0.005328337
1633566_at	CG7694-RA	CG7694 gene product from transcript CG7694-RA	1.928	0.004809069
1633237_at	CG4472-RA	Imaginal disc growth factor 1	1.927	0.037383687
1628602_a_at	CG9248-RB	CG9248 gene product from transcript CG9248-RB	1.925	2.21E-04
1638882_at	CG7642-RA	rosy	1.925	0.022073697
1633951_at	CG7769-RA	piccolo	1.923	3.51E-04
1629229_a_at	CG3992-RA	serpent	1.922	0.024780674
1637190_at	CG9149-RA	CG9149 gene product from transcript CG9149-RA	1.920	0.010493888
1631149_s_at	CG10922-RB	La autoantigen-like	1.907	2.54E-04
1622896_at	CG13848-RA	prolonged depolarization afterpotential (PDA) is not ap	1.906	0.007581246
1637971_a_at	CG8129-RB	CG8129 gene product from transcript CG8129-RB	1.904	0.015772756
1634344_at	CG4180-RA	lethal (2) 35Bg	1.903	7.26E-04
1623641_at	CG32856-RA	CG32856 gene product from transcript CG32856-RB	1.902	7.48E-04
1622960_at	CG14902-RA	death executioner caspase related to Apopain/Yama	1.901	0.04233018
1624986_at	CG9836-RA	CG9836 gene product from transcript CG9836-RA	1.894	5.77E-04
1632593_at	CG32147-RA	CG32147 gene product from transcript CG32147-RA	1.891	2.02E-04
1634848_at	CG16705-RA	Spatzle-Processing Enzyme	1.888	0.04697614
1634222_at	CG18596-RA	CG18596 gene product from transcript CG18596-RA	1.887	2.35E-04
1627512_at	CG31915-RA	CG31915 gene product from transcript CG31915-RA	1.885	0.029711666
1626240_s_at	CG6236-RB	CG6236 gene product from transcript CG6236-RB	1.885	0.001099022
1625500_at	CG11482-RA	CG11482 gene product from transcript CG11482-RB	1.884	0.004672722
1630390_at	CG5602-RA	DNA ligase I	1.880	0.00708321
1624280_at	CG2263-RA	CG2263 gene product from transcript CG2263-RA	1.873	0.005670883
1631519_at	CG9143-RA	CG9143 gene product from transcript CG9143-RA	1.863	0.006190959
1640835_a_at	CG5320-RA	Glutamate dehydrogenase	1.863	0.003665939
1631205_a_at	CG6084-RA	CG6084 gene product from transcript CG6084-RC	1.863	0.04733402
1636948_a_at	CG6778-RA	Glycyl-tRNA synthetase	1.861	5.56E-04
1624807_at	CG5629-RB	Phosphopantothenoylcysteine synthetase	1.859	0.010597768
1639469_a_at	CG9441-RB	Punch	1.855	0.034885757
1628950_at	CG10178-RA	CG10178 gene product from transcript CG10178-RB	1.849	0.002810941
1634739_a_at	CG4001-RA	Phosphofructokinase	1.839	0.008305934
1634152_at	CG12242-RA	Glutathione S transferase D5	1.834	0.025787404
1639509_at	CG12396-RA	CG12396 gene product from transcript CG12396-RA	1.832	0.015157592
1630324_at	CG7989-RA	wicked	1.829	5.79E-04
1623722_at	CG10328-RA	nonA-like	1.829	0.009512022
1623485_at	CG6358-RA	Xeroderma pigmentosum group A-like	1.828	0.002477636
1636071_a_at	CG7035-RA	cap binding protein 80	1.828	1.24E-05
1632854_s_at	CG5939-RB	Paramyosin	1.825	0.048882037
1639584_at	CG2110-RA	CG2110 gene product from transcript CG2110-RA	1.821	0.034403764
1623193_at	CG33123-RA	CG33123 gene product from transcript CG33123-RA	1.819	0.001099777
1625724_at	CG14940-RA	CG44007 gene product from transcript CG44007-RG	1.814	0.003336879
1625107_at	CG4934-RA	brainiac	1.813	0.003205006
1630408_s_at	CG7033-RC	CG7033 gene product from transcript CG7033-RA	1.811	0.002246746
1624617_at	CG1665-RA	CG1665 gene product from transcript CG1665-RA	1.809	0.020541435
1634081_at	CG6258-RA	Replication factor C 38kD subunit	1.809	0.001230874
1626150_at	CG1374-RA	teashirt	1.808	0.013230551
1635713_at	CG13185-RA	CG13185 gene product from transcript CG13185-RD	1.805	0.045423012
1632565_at	CG6046-RA	Bicoid interacting protein 1	1.801	0.033758756
1624180_at	CG2222-RA	CG2222 gene product from transcript CG2222-RB	1.799	0.005006902
1641035_a_at	CG5854-RA	CG5854 gene product from transcript CG5854-RA /// --	1.795	0.013105717
1632402_at	CG6744-RA	CG6744 gene product from transcript CG6744-RA	1.793	0.008188061
1631072_at	CG9512-RA	CG9512 gene product from transcript CG9512-RA	1.785	0.010260337
1635645_at	CG9319-RA	CG9319 gene product from transcript CG9319-RB /// --	1.784	0.005794048
1627915_a_at	CG8893-RA	Glyceraldehyde 3 phosphate dehydrogenase 2	1.782	6.40E-05
1627564_s_at	CG8954-RA	CG8954 gene product from transcript CG8954-RA	1.782	0.012666535
1626968_at	CG7207-RA	ceramide transfer protein	1.781	0.00256976
1638469_s_at	AY180918	CG3857 gene product from transcript CG3857-RA	1.778	6.47E-04
1625038_s_at	CG10895-RA	loki	1.776	2.84E-04
1624419_a_at	CG8439-RA	T-complex Chaperonin 5	1.775	0.002378092
1627131_at	CG12372-RA	CG12372 gene product from transcript CG12372-RA	1.773	0.001240936
1632245_a_at	CG9453-RB	Serpin 42Da	1.768	0.001554659
1622926_at	CG5776-RA	CG5776 gene product from transcript CG5776-RB	1.766	0.003339733
1628432_at	CG5590-RA	CG5590 gene product from transcript CG5590-RA	1.766	0.007606529
1625354_at	CG1257-RA	alpha-Esterase-3	1.765	1.43E-05
1629026_at	CG3821-RA	Aspartyl-tRNA synthetase	1.760	8.69E-04
1625594_s_at	CG15862-RA	cAMP-dependent protein kinase R2	1.755	7.06E-04
1640199_at	CG14670-RA	Holocarboxylase synthetase	1.754	1.46E-04
1634113_at	CG13695-RB	geko	1.754	0.00757914
1624193_s_at	CG3983-RB	nucleostemin 1	1.748	0.002982253

1631877_a_at	CG6335-RA	Histidyl-tRNA synthetase	1.746	0.001741324
1632277_a_at	CG6170-RA	CG6170 gene product from transcript CG6170-RA	1.745	0.013434975
1626170_at	CG18445-RA	oysgedart	1.743	0.004417544
1630638_at	CG1828-RA	CG1828 gene product from transcript CG1828-RB	1.740	0.00493557
1624756_at	CG5009-RA	CG5009 gene product from transcript CG5009-RA	1.738	0.017155673
1634586_at	CG9614-RJ	pipe	1.735	0.004968485
1626368_at	CG4162-RA	CG4162 gene product from transcript CG4162-RA	1.730	0.003129177
1626638_at	CG5202-RA	CG5202 gene product from transcript CG5202-RA /// Q	1.729	0.006883962
1630865_s_at	CG11337-RB	CG11337 gene product from transcript CG11337-RD	1.728	0.003041473
1635089_at	CG8253-RA	tungus	1.724	0.001490218
1637847_at	CG5362-RA	Malate dehydrogenase 1	1.721	0.001571708
1629150_at	CG17337-RA	CG17337 gene product from transcript CG17337-RA	1.718	0.003422973
1629342_s_at	CG1746-RA	CG1746 gene product from transcript CG1746-RA	1.712	0.015014326
1635047_s_at	CG18214-RA	CG18214 gene product from transcript CG18214-RA	1.711	6.08E-04
1624135_at	CG1898-RA	CG1898 gene product from transcript CG1898-RA	1.708	0.005268653
1626412_at	CG8549-RA	CG8549 gene product from transcript CG8549-RA	1.706	2.54E-04
1637542_s_at	CG32956-RA	CG13399 gene product from transcript CG13399-RA //	1.705	0.006433905
1633215_at	CG12006-RA	CG12006 gene product from transcript CG12006-RA	1.702	0.006489258
1631426_at	CG32412-RA	Glutaminyl cyclase	1.702	0.001396578
1631433_at	CG3335-RA	CG3335 gene product from transcript CG3335-RA	1.698	8.63E-04
1633234_at	CG2807-RA	CG2807 gene product from transcript CG2807-RB	1.692	5.79E-04
1632980_at	CG3902-RA	CG3902 gene product from transcript CG3902-RA	1.689	3.30E-04
1641231_at	CG1621-RA	Corepressor of Pangolin	1.687	0.002457242
1634700_at	CG8258-RA	CG8258 gene product from transcript CG8258-RA	1.686	0.002697775
1634065_s_at	CG17654-RB	Enolase	1.685	0.003533698
1626460_at	CG2658-RA	CG2658 gene product from transcript CG2658-RB	1.683	0.006588065
1638852_at	CG10675-RA	CG10675 gene product from transcript CG10675-RA	1.683	1.57E-04
1626942_at	CG9996-RA	CG9996 gene product from transcript CG9996-RB	1.682	0.008704587
1630347_at	CG5589-RA	CG5589 gene product from transcript CG5589-RA	1.679	0.029174471
1624953_at	CG4120-RA	CG4120 gene product from transcript CG4120-RA	1.675	1.13E-04
1635449_s_at	CG9285-RA	Dipeptidase B	1.675	0.010169532
1625758_s_at	CG6375-RB	pitchoune	1.674	0.047751367
1623122_at	CG17294-RA	CG17294 gene product from transcript CG17294-RA	1.674	0.0071255
1638011_a_at	CG6148-RA	Putative Achaete Scute Target 1	1.671	4.41E-04
1632418_s_at	CG9755-RA	pumilio	1.670	0.007546981
1628352_a_at	CG8798-RB	Lon protease	1.669	0.002491571
1636468_a_at	CG5033-RA	CG5033 gene product from transcript CG5033-RA	1.658	0.026317228
1626845_at	CG8351-RA	CG8351 gene product from transcript CG8351-RA	1.656	3.33E-04
1633353_s_at	CG18408-RI	CG18408 gene product from transcript CG18408-RI	1.649	0.022984264
1637169_at	CG12202-RA	CG12202 gene product from transcript CG12202-RA	1.649	4.39E-04
1627109_at	CG8542-RA	Heat shock protein cognate 5	1.647	0.004832729
1634636_at	CG6426-RA	CG6426 gene product from transcript CG6426-RA	1.644	0.010333432
1625856_at	CG10798-RA	diminutive	1.642	0.014672076

1628665_at	CG6841-RA	CG6841 gene product from transcript CG6841-RA	1.642	5.49E-04
1637217_at	CG5714-RA	ecdysoneless	1.641	2.17E-04
1632043_a_at	CG9198-RA	shattered	1.640	0.012816613
1638410_at	CG5131-RA	CG5131 gene product from transcript CG5131-RA	1.640	0.005567978
1634316_at	CG1582-RA	CG1582 gene product from transcript CG1582-RA	1.640	0.002664963
1632946_at	CG40293-RA	Ste20-like kinase	1.638	0.014160253
1636932_at	CG9886-RA	CG9886 gene product from transcript CG9886-RB	1.638	0.010733922
1634940_at	CG3943-RA	CG3943 gene product from transcript CG3943-RA	1.636	0.04150644
1628257_s_at	CG12013-RB	CG12013 gene product from transcript CG12013-RC	1.636	0.016961023
1638415_at	CG11001-RA	FK506-binding protein 2	1.635	0.003743824
1639766_at	CG3127-RA	Phosphoglycerate kinase	1.633	0.016749794
1630623_a_at	CG8058-RB	alpha/beta hydrolase 1	1.632	0.010296342
1640496_at	CG5722-RA	Niemann-Pick type C-1a	1.631	0.001503809
1639665_at	CG7878-RA	CG7878 gene product from transcript CG7878-RA	1.630	8.49E-05
1639489_at	CG3700-RA	CG3700 gene product from transcript CG3700-RB /// --	1.629	9.16E-05
1626818_at	CG13097-RA	CG13097 gene product from transcript CG13097-RA	1.624	0.001893121
1633798_at	CG5654-RA	ypsilon schachtel	1.622	6.15E-04
1637091_a_at	CG9772-RB	CG9772 gene product from transcript CG9772-RA	1.621	0.013776829
1628537_s_at	CG8368-RB	CG8368 gene product from transcript CG8368-RB	1.618	0.014013688
1628813_at	CG31381-RA	CG11089 gene product from transcript CG11089-RD ///	1.616	0.030386522
1631007_at	CG5371-RA	Ribonucleoside diphosphate reductase large subunit	1.616	0.003517713
1634695_at	CG2684-RA	lodestar	1.609	0.004681077
1627916_s_at	CG1799-RC	raspberry	1.609	0.006575399
1630178_at	CG12082-RA	CG12082 gene product from transcript CG12082-RB	1.607	0.005029804
1639259_at	CG9006-RA	Enigma	1.606	0.0021889
1632818_at	CG32281-RA	CG32281 gene product from transcript CG32281-RA	1.606	0.005650025
1631993_s_at	CG6058-RB	Aldolase	1.599	0.006915187
1640594_at	CG3011-RA	CG3011 gene product from transcript CG3011-RA /// --	1.597	0.010178853
1637758_at	CG7737-RA	CG7737 gene product from transcript CG7737-RA	1.595	0.003675458
1638581_at	CG14224-RA	Ubiquilin	1.594	0.002618028
1623312_s_at	CG11836-RB	CG11836 gene product from transcript CG11836-RB	1.594	0.003973177
1637576_s_at	CG8839-RA	CG8839 gene product from transcript CG8839-RA	1.594	0.015467422
1623164_a_at	CG8127-RB	Ecdysone-induced protein 75B	1.591	0.044770837
1638080_at	CG13658-RA	CG13658 gene product from transcript CG13658-RB	1.590	0.01889885
1637353_at	CG3318-RA	Dopamine N acetyltransferase	1.588	0.01346089
1623393_a_at	CG3277-RA	CG3277 gene product from transcript CG3277-RC	1.586	0.013565322
1640595_at	CG12301-RA	CG12301 gene product from transcript CG12301-RA	1.583	0.029998414
1638007_at	CG1542-RA	CG1542 gene product from transcript CG1542-RA	1.583	0.012921278
1626536_at	CG6776-RA	Glutathione S transferase O3	1.583	0.001551238
1631198_at	CG2173-RA	CG2173 gene product from transcript CG2173-RA	1.582	0.001189266
1641199_a_at	CG1544-RA	CG1544 gene product from transcript CG1544-RA	1.582	0.014861994
1626125_at	CG3157-RA	gamma-Tubulin at 23C	1.581	0.048103657
1636636_at	CG11771-RA	CG11771 gene product from transcript CG11771-RA	1.581	5.76E-04

1627134_at	CG6291-RA	Aminopeptidase P	1.579	0.001082065
1632119_s_at	CG8024-RC	lightoid	1.577	0.025768278
1623383_at	CG7507-RA	Dynein heavy chain 64C	1.573	0.003554492
1628099_at	CG6815-RA	belphegor	1.573	9.65E-04
1635393_s_at	GM02640	Ecdysone-induced protein 75B	1.572	0.001215019
1638070_at	CG10418-RA	CG10418 gene product from transcript CG10418-RA	1.572	0.03060154
1640608_at	CG3329-RA	Proteasome beta2 subunit	1.571	6.46E-04
1629881_at	CG12752-RA	NTF2-related export protein 1	1.568	0.003577825
1627183_at	CG14906-RA	CG14906 gene product from transcript CG14906-RC ///	1.567	0.001360359
1641004_a_at	CG3164-RA	CG3164 gene product from transcript CG3164-RA	1.567	0.008779556
1636180_at	CG4600-RA	yippee interacting protein 2	1.567	0.037398618
1627249_a_at	CG4180-RB	lethal (2) 35Bg	1.565	3.03E-04
1641134_at	CG7277-RA	CG7277 gene product from transcript CG7277-RA	1.564	0.00835605
1626729_at	CG3803-RA	CG3803 gene product from transcript CG3803-RA	1.563	0.004741661
1634096_at	CG3752-RA	Aldehyde dehydrogenase	1.563	0.034565724
1624272_at	CG10618-RA	CG10618 gene product from transcript CG10618-RB	1.563	0.002964375
1634314_s_at	CG7176-RA	Isocitrate dehydrogenase	1.562	6.87E-04
1639554_at	CG3333-RA	Nucleolar protein at 60B	1.562	0.0418354
1641298_at	CG10535-RA	Elongator complex protein 1	1.561	0.010026704
1632827_a_at	CG8153-RB	mutagen-sensitive 210	1.561	0.003923746
1636530_s_at	CG9277-RB	beta-Tubulin at 56D	1.560	0.001269578
1635832_at	CG12413-RA	CG12413 gene product from transcript CG12413-RA	1.560	7.67E-05
1627349_at	CG4539-RA	Bekka	1.558	0.022578368
1623864_at	CG6321-RA	CG6321 gene product from transcript CG6321-RA	1.558	0.044981387
1637887_at	CG1703-RA	CG1703 gene product from transcript CG1703-RA	1.558	0.006761643
1632251_s_at	CG6181-RA	CG6181 gene product from transcript CG6181-RA	1.556	1.06E-04
1624321_at	CG8556-RA	CG8556 gene product from transcript CG8556-RB	1.556	0.002188316
1635984_at	CG31385-RA	CG7649 gene product from transcript CG7649-RC	1.553	0.002449332
1623271_a_at	CG7933-RA	janus A	1.553	0.003747138
1623715_at	CG4567-RA	iconoclast	1.552	0.006543724
1635000_at	CG9480-RB	CG9480 gene product from transcript CG9480-RA	1.542	0.001220864
1625297_at	CG8128-RA	CG8128 gene product from transcript CG8128-RA	1.541	7.46E-05
1628481_at	CG7494-RA	mitochondrial ribosomal protein L1	1.541	0.004957836
1626266_at	CG10059-RA	CG10059 gene product from transcript CG10059-RA	1.539	6.59E-04
1634364_s_at	CG32130-RA	starvin	1.539	0.012907028
1625923_s_at	CG32593-RA	Flotillin-2	1.538	0.001167682
1632689_at	CG1307-RB	CG1307 gene product from transcript CG1307-RC	1.538	1.94E-04
1639507_at	CG6666-RA	Succinate dehydrogenase C	1.538	9.02E-04
1624150_at	CG4535-RA	FK506-binding protein FKBP59	1.536	0.008942708
1637715_a_at	CG13929-RA	methyltransferase-like	1.536	0.002486272
1633106_at	CG6662-RA	Glutathione S transferase O1	1.535	0.011285581
1625458_a_at	CG7935-RA	moleskin	1.534	0.0258634
1628492_at	CG18315-RA	Adenine phosphoribosyltransferase	1.533	0.019214202
1628213_s_at	CG5887-RA	CG5887 gene product from transcript CG5887-RA	1.533	0.032448683
1630344_at	CG6724-RA	CG6724 gene product from transcript CG6724-RB	1.533	0.006711513
1627638_s_at	CG5374-RB	Tcp1-like	1.532	0.002429322
1631223_at	CG2100-RA	CG2100 gene product from transcript CG2100-RB	1.531	0.009050993
1634028_s_at	CG6203-RA	CG6203 gene product from transcript CG6203-RC	1.529	0.04000817
1628435_at	CG16901-RA	squid	1.529	0.001872392
1634243_a_at	CG4673-RB	Npl4 ortholog	1.528	4.61E-04
1626850_s_at	CG31953-RA	glaikit	1.527	0.015734637
1633857_at	CG13659-RA	CG13659 gene product from transcript CG13659-RA	1.527	0.012814581
1637327_at	CG11376-RA	Zizimin-related	1.526	0.006760646
1625405_at	CG4182-RA	CG4182 gene product from transcript CG4182-RA	1.526	0.004691752
1639205_s_at	CG10128-RC	transformer 2	1.523	0.001660005
1639224_at	CG9273-RA	Replication protein A2	1.522	0.001744668
1639536_at	CG6379-RA	CG6379 gene product from transcript CG6379-RA	1.522	0.010166198
1626223_at	CG2543-RA	CG2543 gene product from transcript CG2543-RA	1.518	0.010668828
1640470_s_at	CG18734-RA	Furin 2	1.515	0.027158719
1641123_at	HDC06513	CG34186 gene product from transcript CG34186-RA	1.513	8.25E-04
1635025_a_at	CG4094-RA	lethal (1) G0255	1.512	0.004971356
1632877_a_at	CG2906-RB	CG2906 gene product from transcript CG2906-RE	1.512	0.003225418
1641469_at	CG9320-RA	nucleostemin 4	1.511	0.002458517
1636919_at	CG9401-RA	mago nashi	1.510	0.017198913
1639349_at	CG8173-RA	CG8173 gene product from transcript CG8173-RA	1.510	0.006952565
1640907_at	CG12055-RA	Glyceraldehyde 3 phosphate dehydrogenase 1	1.509	0.002703619
1631853_at	CG6610-RA	CG6610 gene product from transcript CG6610-RA	1.508	0.009311939
1631183_at	CG9366-RA	rho-like	1.504	9.78E-04
1629790_at	CG11267-RA	CG11267 gene product from transcript CG11267-RA	1.503	0.022569781
1638887_a_at	CG6339-RA	CG6339 gene product from transcript CG6339-RE	1.503	0.004764688
1637349_at	CG7762-RA	Regulatory particle non-ATPase 1	1.502	0.003432238
1635664_at	CG13761-RB	Buzidau	1.502	0.013978673
1630632_s_at	CG2982-RB	CG2982 gene product from transcript CG2982-RB	1.501	0.006141287
1637265_at	CG7550-RA	CG7550 gene product from transcript CG7550-RA	0.666	0.009856358
1627796_s_at	SD05230	grappa	0.665	0.028170347
1636673_s_at	CG1856-RF	tramtrack	0.663	0.020230813
1626385_s_at	CG5643-RB	widerborst	0.663	0.001304152
1629493_at	CG13401-RA	CG13401 gene product from transcript CG13401-RA	0.663	0.020496717
1637643_at	CG4435-RA	CG4435 gene product from transcript CG4435-RA	0.662	3.82E-07
1636311_at	CG9042-RA	Glycerol 3 phosphate dehydrogenase	0.662	0.02971372
1639783_s_at	CG5422-RD	CG5422 gene product from transcript CG5422-RD	0.661	0.005107276
1624816_at	CG30283-RA	CG30283 gene product from transcript CG30283-RB	0.661	0.034650736
1640239_at	CG30149-RB	rigor mortis	0.660	1.46E-04
1641130_at	CG31731-RA	CG31731 gene product from transcript CG31731-RB	0.660	0.004630713
1627844_at	CG2060-RA	Cytochrome P450-4e2	0.659	0.00112564
1632680_at	CG7480-RA	Polypeptide N-acetylgalactosaminyltransferase 35A	0.658	5.39E-04

1632648_at	CG32922-RA	CG43161 gene product from transcript CG43161-RD	0.657	0.03288023
1637839_at	CG1751-RA	Spase 25-subunit	0.655	1.32E-04
1624337_at	CG1527-RA	Ribosomal protein S14a /// Ribosomal protein S14b	0.654	2.84E-04
1630930_a_at	CG8205-RC	fusilli	0.653	9.76E-04
1638161_at	CG11115-RA	CG11115 gene product from transcript CG11115-RA	0.653	0.018895539
1629029_at	CG5160-RA	CG5160 gene product from transcript CG5160-RA	0.651	0.038203683
1631498_a_at	CG14307-RC	fruitless	0.649	0.011007547
1636403_at	CG5276-RA	CG5276 gene product from transcript CG5276-RA	0.648	0.005345615
1623607_at	CG13431-RA	UDP-GlcNAc:a-3-D-mannoside-beta-1,2-N-acetylgluco	0.647	0.004016214
1640339_at	CG8198-RA	lethal (1) G0136	0.647	0.001060369
1622933_at	CG10344-RB	CG10344 gene product from transcript CG10344-RA	0.647	8.38E-04
1641542_at	CG14616-RE	lethal (1) G0196	0.647	0.006775522
1632835_at	CG9376-RA	CG9376 gene product from transcript CG9376-RA	0.646	0.003196791
1623080_at	CG18410-RA	Uracil-DNA degrading factor	0.646	0.003047038
1634623_a_at	CG8687-RA	CG8687 gene product from transcript CG8687-RD	0.644	0.002438719
1640948_at	CG7580-RA	CG7580 gene product from transcript CG7580-RA	0.644	0.006318075
1625125_at	CG5599-RA	CG5599 gene product from transcript CG5599-RA	0.644	0.03510699
1641714_at	CG12338-RA	CG12338 gene product from transcript CG12338-RA	0.642	0.005218165
1627845_at	CG6673-RB	Glutathione S transferase O2	0.642	0.001165239
1633483_a_at	CG14207-RA	CG14207 gene product from transcript CG14207-RB	0.641	2.69E-04
1636340_at	CG3910-RA	mitochondrial transcription factor B2	0.641	0.043083847
1629725_at	CG3641-RB	CG33331 gene product from transcript CG33331-RA	0.639	0.019418357
1633089_a_at	CG12052-RJ	longitudinals lacking	0.636	9.38E-04
1628126_at	CG31202-RA	CG31202 gene product from transcript CG31202-RA	0.635	0.001664992
1641191_s_at	CG17932-RA	CG17932 gene product from transcript CG17932-RB	0.635	0.04223956
1628921_s_at	SD11801	modifier of mdg4 /// CG42769 gene product from transc	0.635	0.004457435
1627384_at	CG6225-RA	CG6225 gene product from transcript CG6225-RB	0.635	0.001568175
1637036_s_at	CG16784-RC	purple	0.633	0.013537128
1633099_at	CG3876-RA	CG3876 gene product from transcript CG3876-RA	0.633	0.008437743
1628562_s_at	CG32491-RT	modifier of mdg4 /// CG42769 gene product from transc	0.632	2.57E-04
1629746_at	CG4041-RA	CG4041 gene product from transcript CG4041-RB	0.631	0.04261316
1637917_s_at	CG1856-RE	tramtrack	0.631	0.01071164
1633491_s_at	CG31866-RA	CG31865 gene product from transcript CG31865-RA //	0.630	3.41E-04
1640127_at	CG6650-RA	CG6650 gene product from transcript CG6650-RA	0.629	0.008700095
1641624_at	CG31957-RA	CG31957 gene product from transcript CG31957-RB	0.629	0.00184016
1631351_s_at	CG12118-RA	CG12118 gene product from transcript CG12118-RB	0.628	0.005002415
1623411_at	CG12052-RN	longitudinals lacking	0.628	0.003327923
1629270_at	CG17958-RA	Serendipity delta	0.628	0.010914986
1623792_a_at	CG9741-RB	Dihydroorotate dehydrogenase	0.628	0.007538241
1624851_at	CG32629-RA	Transport and Golgi organization 13	0.626	0.012646571
1639083_at	CG1945-RC	fat facets	0.625	0.009429477
1635678_at	CG11299-RA	Sestrin	0.623	0.008578442
1638074_at	CG6673-RA	Glutathione S transferase O2	0.622	0.003958777

1631595_at	CG13393-RA	lethal (2) k12914	0.621	0.009879119
1639410_at	CG7013-RA	Mesencephalic astrocyte-derived neurotrophic factor	0.621	0.004924618
1628849_at	HDC07332	CG8201 gene product from transcript CG8201-RV	0.621	0.00188239
1630203_at	CG5682-RA	CG5682 gene product from transcript CG5682-RA	0.619	0.003487183
1637142_at	CG9547-RA	CG9547 gene product from transcript CG9547-RA	0.617	8.53E-04
1633985_at	HDC15359	CG6364 gene product from transcript CG6364-RD	0.616	0.008028825
1635754_at	CG11987-RA	tango	0.615	1.46E-04
1625279_a_at	CG10619-RA	tailup	0.612	0.03589902
1627794_a_at	CG5063-RB	Translin associated factor X	0.612	0.001403984
1630519_at	CG8882-RA	CG8882 gene product from transcript CG8882-RA	0.611	3.28E-04
1641118_at	CG5889-RA	Malic enzyme b	0.610	0.009691994
1631242_at	CG4029-RA	jumeau	0.610	0.016876327
1630224_at	CG6410-RA	Sorting nexin 16	0.609	0.006891764
1625283_at	CG32491-RM	modifier of mdg4	0.607	0.010529318
1637771_s_at	CG7220-RA	CG7220 gene product from transcript CG7220-RD	0.606	5.65E-04
1638466_a_at	CG4370-RA	Inwardly rectifying potassium channel 2	0.606	0.01349143
1623887_at	CG12367-RA	CG12367 gene product from transcript CG12367-RA	0.606	0.019997472
1627605_at	CG1071-RA	E2F transcription factor 2	0.605	2.71E-04
1625566_a_at	CG9293-RB	CG9293 gene product from transcript CG9293-RB	0.604	7.45E-04
1631340_at	CG8261-RA	G protein gamma 1	0.604	0.01811019
1637721_at	CG3301-RB	CG3301 gene product from transcript CG3301-RB	0.602	0.010682763
1635587_s_at	CG4050-RA	CG4050 gene product from transcript CG4050-RA	0.601	0.002670777
1641458_s_at	CG1511-RD	Eph receptor tyrosine kinase	0.601	3.16E-04
1637826_at	CG10623-RA	CG10623 gene product from transcript CG10623-RA	0.598	0.014830509
1625756_at	CG6656-RA	CG6656 gene product from transcript CG6656-RA	0.597	0.023042012
1638101_at	CG15528-RA	CG15528 gene product from transcript CG15528-RB	0.595	0.014979204
1631408_at	CG18024-RA	SoxNeuro	0.594	6.84E-04
1639139_a_at	CG8874-RB	Fps oncogene analog	0.593	0.002128981
1626990_s_at	CG4123-RB	Multiple inositol polyphosphate phosphatase 1	0.592	0.003065844
1630456_at	CG32156-RE	Myosin binding subunit	0.590	0.001187458
1630957_s_at	CG11128-RA	slimfast	0.589	0.014882051
1625989_at	CG6144-RA	CG6144 gene product from transcript CG6144-RB	0.588	0.028670797
1633451_at	CG32634-RA	CG1987 gene product from transcript CG1987-RA	0.587	0.03508023
1624051_at	CG13850-RA	CG13850 gene product from transcript CG13850-RA	0.585	0.002637098
1628914_at	CG3194-RA	Heparan sulfate C5-epimerase	0.585	0.002153111
1636025_at	CG1167-RA	Ras oncogene at 64B	0.579	0.001511893
1626158_s_at	CG9655-RA	nessy	0.578	0.001264002
1637584_at	CG4445-RA	polypeptide GalNAc transferase 3	0.575	0.011208463
1632478_a_at	CG6622-RB	Protein C kinase 53E	0.574	0.007963029
1630723_a_at	CG8708-RB	CG8708 gene product from transcript CG8708-RC /// --	0.574	0.004519464
1634099_at	CG11111-RA	retinal degeneration B	0.574	0.001120711
1631674_at	CG12366-RA	O-fucosyltransferase 1	0.573	0.014372858
1626180_at	CG3495-RA	GDP-4-keto-6-deoxy-D-mannose 3,5-epimerase/4-reduc	0.573	0.010827797

1623549_at	CG1973-RA	CG1973 gene product from transcript CG1973-RB	0.573	6.67E-04
1631527_at	CG5642-RA	CG5642 gene product from transcript CG5642-RA	0.573	7.52E-04
1630857_s_at	CG3059-RD	CG3059 gene product from transcript CG3059-RD	0.573	9.38E-04
1623770_at	CG1673-RA	CG1673 gene product from transcript CG1673-RA	0.571	0.016168173
1638001_a_at	CG8889-RA	Metallophosphoesterase	0.569	0.00539462
1641629_at	CG7402-RA	CG7402 gene product from transcript CG7402-RA	0.567	0.025999296
1639308_at	CG32491-RF	modifier of mdg4	0.565	0.001798739
1624164_s_at	CG6207-RA	CG6207 gene product from transcript CG6207-RE	0.564	0.013673544
1632711_at	CG7864-RA	Peroxin 10	0.562	0.012550153
1632447_at	CG10851-RD	CG10851 gene product from transcript CG10851-RN	0.561	0.001826772
1639636_at	CG1869-RA	CG1869 gene product from transcript CG1869-RA	0.561	0.011118979
1638963_s_at	CG8448-RD	CG8448 gene product from transcript CG8448-RD	0.560	0.007635833
1623020_at	CG31937-RA	CG31937 gene product from transcript CG31937-RA	0.556	0.003558865
1629180_at	CG14789-RA	O-fucosyltransferase 2	0.554	0.004180943
1625955_a_at	CG32000-RE	CG32000 gene product from transcript CG32000-RH	0.554	0.030201737
1632306_at	CG9467-RA	CG9467 gene product from transcript CG9467-RA	0.554	1.97E-05
1625599_at	CG31152-RA	CG31152 gene product from transcript CG31152-RB	0.553	0.001924289
1635003_at	CG6697-RA	Ubiquitin-like domain-containing C-terminal domain ph	0.546	1.97E-04
1632345_at	CG8353-RA	CG8353 gene product from transcript CG8353-RB	0.541	0.004282749
1624618_at	CG3870-RA	CG3870 gene product from transcript CG3870-RA	0.540	5.51E-04
1623838_s_at	CG4629-RA	CG4629 gene product from transcript CG4629-RE	0.537	0.001610702
1634429_at	CG8360-RA	CG8360 gene product from transcript CG8360-RC	0.537	0.001027767
1631742_s_at	CG32491-RQ	modifier of mdg4	0.537	0.00332743
1630156_at	CG12859-RA	CG12859 gene product from transcript CG12859-RA	0.534	0.001371401
1638217_at	CG10166-RA	CG10166 gene product from transcript CG10166-RA	0.534	0.002217993
1625058_at	CG9889-RA	CG9889 gene product from transcript CG9889-RA	0.533	9.29E-04
1635342_at	CG10306-RA	CG10306 gene product from transcript CG10306-RA	0.528	4.92E-06
1633763_at	CG2855-RA	anterior pharynx defective 1	0.528	5.19E-04
1630244_s_at	CG31809-RA	CG31809 gene product from transcript CG31809-RB ///	0.526	0.006198904
1638875_at	CG6542-RA	Egg-derived tyrosine phosphatase	0.525	0.008692758
1629382_at	CG9670-RA	falten	0.524	0.010051955
1626165_at	CG3754-RA	Phosphodiesterase 9	0.524	0.002821438
1633152_at	CG32491-RA	modifier of mdg4	0.523	0.005434811
1625794_at	CG5830-RA	CG5830 gene product from transcript CG5830-RA	0.519	0.001193476
1629129_at	CG7525-RA	Tie-like receptor tyrosine kinase	0.519	3.18E-04
1634906_at	CG4898-RC	alpha-Tubulin at 84D /// Tropomyosin 1	0.516	0.003381481
1625589_at	CG4840-RA	centrosomin's beautiful sister	0.516	4.03E-04
1638041_at	CG32491-RH	modifier of mdg4	0.516	0.012785054
1636765_at	CG32491-RJ	modifier of mdg4	0.516	0.005565568
1640077_at	CG3692-RA	Calpain C	0.514	0.002360077
1629048_s_at	CG9674-RD	CG9674 gene product from transcript CG9674-RD	0.510	0.012636561
1627835_a_at	CG31973-RA	Chitin deacetylase-like 5	0.510	0.004650571
1630841_at	CG8668-RA	CG8668 gene product from transcript CG8668-RA	0.509	0.002753527

1638722_at	CG16807-RA	roquin	0.507	1.27E-04
1632381_at	CG14226-RA	domeless	0.506	0.026396735
1634597_a_at	CG31085-RB	CG33970 gene product from transcript CG33970-RA	0.504	1.34E-04
1638053_at	CG10842-RA	Cytochrome P450-4p1	0.504	0.00141751
1632956_at	CG3021-RA	CG3021 gene product from transcript CG3021-RA	0.504	2.56E-05
1633200_at	CG9449-RA	CG9449 gene product from transcript CG9449-RH	0.493	0.001252127
1635175_at	CG17121-RA	CG17121 gene product from transcript CG17121-RA	0.493	2.63E-04
1633010_s_at	CG30382-RA	CG30382 gene product from transcript CG30382-RB ///	0.489	1.35E-04
1635293_s_at	CG8336-RB	CG8336 gene product from transcript CG8336-RB	0.488	9.94E-04
1630013_s_at	CG5025-RB	Selenophosphate synthetase 2	0.487	0.001727202
1640082_at	CG6896-RA	CG6896 gene product from transcript CG6896-RB	0.485	0.004057791
1627773_a_at	CG15102-RA	Juvenile hormone epoxide hydrolase 2	0.478	0.00897616
1626461_at	CG4357-RA	Heat shock protein 60 related /// sodium chloride cotran	0.477	0.001494621
1634822_a_at	CG10344-RA	CG10344 gene product from transcript CG10344-RA	0.470	6.89E-04
1636606_at	CG7623-RA	slalom	0.470	0.007295398
1638060_at	CG10077-RB	CG10077 gene product from transcript CG10077-RA	0.470	0.006545921
1625613_at	CG11899-RA	CG11899 gene product from transcript CG11899-RA //	0.466	0.01194808
1640476_at	CG4709-RA	CG4709 gene product from transcript CG4709-RA	0.465	8.48E-04
1628150_a_at	CG9449-RB	CG9449 gene product from transcript CG9449-RH	0.461	0.002680312
1623238_at	CG5618-RA	CG5618 gene product from transcript CG5618-RD	0.461	0.001131506
1632952_at	CG4598-RA	CG4598 gene product from transcript CG4598-RB	0.460	0.002261482
1628604_at	CG5893-RA	Dichaete	0.458	0.005151275
1627841_at	CG11170-RB	CG11170 gene product from transcript CG11170-RB	0.456	0.001632855
1637032_at	CG14517-RA	CG14517 gene product from transcript CG14517-RA	0.449	3.05E-04
1637478_s_at	CG31000-RB	hephaestus	0.448	0.015236953
1631082_at	CG10851-RF	CG10851 gene product from transcript CG10851-RN	0.445	0.003556395
1639660_s_at	CG10550-RB	CG10550 gene product from transcript CG10550-RD	0.443	0.044518627
1641021_a_at	CG6297-RB	CG6297 gene product from transcript CG6297-RC	0.442	4.49E-06
1627953_at	CG32491-RI	modifier of mdg4	0.440	0.004287487
1630359_at	CG31810-RA	CG31810 gene product from transcript CG31810-RA	0.439	0.001603798
1640097_at	CG12002-RA	Peroxidasin	0.437	0.03795444
1631903_at	CG14062-RA	CG14062 gene product from transcript CG14062-RB	0.436	0.010198534
1633957_s_at	CG5316-RC	CG5316 gene product from transcript CG5316-RC	0.435	3.64E-04
1638905_s_at	CG4839-RB	CG4839 gene product from transcript CG4839-RA	0.433	0.002038653
1624533_s_at	CG8651-RA	trithorax	0.427	2.59E-04
1636841_at	CG12130-RA	Peptidyl-alpha-hydroxyglycine-alpha-amidating lyase 1	0.423	0.001001437
1635910_at	CG7331-RA	Autophagy-specific gene 13	0.423	0.010410408
1624577_at	CG17907-RA	Acetylcholine esterase	0.414	6.04E-04
1639095_at	CG10426-RA	CG10426 gene product from transcript CG10426-RA	0.414	5.90E-04
1629784_a_at	CG6976-RC	Myosin 28B1	0.411	0.009643578
1634052_s_at	CG32491-RA	modifier of mdg4	0.408	0.00108066
1628839_at	CG17110-RA	CG17110 gene product from transcript CG17110-RA	0.404	0.005413123
1640867_s_at	CG7223-RB	heartless	0.395	0.004595591

1634726_s_at	CG18478-RA	CG18478 gene product from transcript CG18478-RA ///	0.392	0.039174657
1622976_at	CG32491-RB	modifier of mdg4	0.389	0.00638984
1641304_s_at	CG9801-RA	CG9801 gene product from transcript CG9801-RC	0.388	0.01899363
1627483_at	CT35609	modifier of mdg4	0.375	0.003102537
1624119_at	CG32486-RD	CG32486 gene product from transcript CG32486-RD	0.373	7.98E-04
1625344_at	CG17608-RA	CG17608 gene product from transcript CG17608-RA	0.373	0.018697241
1638807_s_at	CG4829-RB	CG4829 gene product from transcript CG4829-RB	0.358	1.22E-04
1631611_at	CG7973-RA	obstructor-H	0.354	0.002425039
1640893_a_at	CG1546-RA	prolyl-4-hydroxylase-alpha SG2	0.351	3.32E-07
1637255_a_at	CG1112-RA	alpha-Esterase-7	0.348	0.003906603
1624700_a_at	CG10175-RA	CG10175 gene product from transcript CG10175-RA	0.345	0.008789139
1636526_at	CG9451-RA	CG9451 gene product from transcript CG9451-RB	0.324	0.009588062
1628458_at	CG13777-RC	milton	0.324	0.004074056
1638020_at	CG10467-RA	CG10467 gene product from transcript CG10467-RA	0.318	0.002852366
1624316_at	CG9737-RA	CG9737 gene product from transcript CG9737-RA	0.318	0.012285276
1625276_a_at	CG7266-RC	Ecdysone-induced protein 28/29kD	0.312	0.00480965
1631500_at	CG32491-RR	modifier of mdg4	0.308	1.91E-04
1634019_at	CG2064-RA	CG2064 gene product from transcript CG2064-RA	0.301	1.84E-05
1623807_a_at	CG13777-RA	milton	0.294	7.27E-04
1628402_a_at	CG3881-RA	CG3881 gene product from transcript CG3881-RA	0.250	0.00200545
1623241_s_at	CG30104-RB	Ecto-5'-nucleotidase 2	0.243	9.44E-05
1633205_at	CG8380-RA	Dopamine transporter	0.228	0.003111536
1640410_at	CG33459-RA	CG33459 gene product from transcript CG33459-RA	0.220	0.043088894
1632715_at	RH40482	Tie-like receptor tyrosine kinase	0.220	2.80E-04
1629713_at	CG9187-RA	CG9187 gene product from transcript CG9187-RA	0.211	6.37E-06
1641681_s_at	CG5288-RA	Galactokinase	0.185	1.65E-05
1638562_a_at	CG3050-RB	CG3050 gene product from transcript CG3050-RA	0.165	0.001272947
1633329_at	CG1629-RA	CG1629 gene product from transcript CG1629-RA	0.165	1.32E-04
1625195_s_at	X59545	CG1829 gene product from transcript CG1829-RA /// s	0.160	0.014580454
1630640_at	CG4594-RA	CG4594 gene product from transcript CG4594-RB	0.154	1.92E-04
1631805_at	CG3397-RA	CG3397 gene product from transcript CG3397-RA	0.135	0.008657239
1641650_at	CG1089-RA	alpha-Esterase-5	0.120	1.27E-05
1636758_s_at	CG11205-RA	CG18853 gene product from transcript CG18853-RA ///	0.047	6.25E-05
1637164_at	CG4800-RA	Translationally controlled tumor protein	0.025	1.80E-05
**Neurogenesis**				
1640377_s_at	CG2849-RB	Ras-related protein	27.693	9.37E-05
1623950_s_at	CG2198-RB	Amalgam	5.647	0.011793503
1624897_at	CG1558-RA	kinetochore Mis12-Ndc80 network component 1	4.842	0.002342818
1623109_at	CG4593-RA	CG4593 gene product from transcript CG4593-RA	4.386	1.33E-04
1628830_at	CG14121-RA	verrocchio	4.145	0.001687216
1628683_at	CG6272-RA	CG6272 gene product from transcript CG6272-RB	3.991	7.80E-05
1624815_at	CG4817-RA	Structure specific recognition protein	3.510	1.29E-04
1627246_at	CG4107-RA	Pcaf /// CG4107 gene product from transcript CG4107-	3.486	0.001702898

1631419_at	CG11129-RA	Yolk protein 3	3.262	0.004988883
1636189_at	CG12306-RA	CG12306 gene product from transcript CG12306-RA	3.172	0.002657874
1637548_at	CG18104-RA	arginase	3.113	0.018552795
1635138_at	CG10772-RA	Furin 1	2.807	0.002189608
1639036_at	CG8857-RC	Ribosomal protein S11	2.797	0.003441904
1639553_at	CG9987-RA	CG9987 gene product from transcript CG9987-RA	2.742	0.004720662
1628952_s_at	CG10772-RB	Furin 1	2.675	0.026843322
1627380_at	CG9633-RA	Replication Protein A 70	2.356	9.89E-05
1626425_at	CG4173-RA	Septin 2	2.331	3.96E-04
1624747_at	CG3181-RA	CG3181 gene product from transcript CG3181-RA	2.321	0.008944592
1634546_at	CG11527-RA	Tiggrin	2.285	0.011669559
1640235_at	CG5123-RA	Wrinkled	2.214	6.14E-04
1628188_at	S.C2R002789	Nucleolar protein at 60B	2.207	0.011176612
1630772_at	CG5557-RA	squeeze	2.198	0.00629058
1626774_s_at	CG13521-RA	roundabout	2.181	0.004077457
1627977_at	CG1671-RA	CG1671 gene product from transcript CG1671-RA	2.165	0.007950474
1634650_at	CG11372-RA	CG11372 gene product from transcript CG11372-RD	2.137	0.001281534
1624686_a_at	CG32498-RO	dunce	2.086	0.004152716
1639040_s_at	CG8291-RA	bedraggled	2.085	0.031007012
1641442_a_at	CG7981-RA	terribly reduced optic lobes	2.077	0.00197086
1639414_at	CG7093-RA	Sno oncogene	2.068	0.003395452
1640365_s_at	CG14021-RB	fuseless	2.047	0.010840835
1632743_a_at	CG1828-RB	CG1828 gene product from transcript CG1828-RB	2.002	1.63E-04
1624138_at	CG5800-RA	CG5800 gene product from transcript CG5800-RA	1.990	4.80E-04
1634722_s_at	CG4143-RA	multiprotein bridging factor 1	1.986	5.57E-05
1627073_a_at	CG10126-RB	CG10126 gene product from transcript CG10126-RB	1.982	1.05E-04
1624668_s_at	CG9422-RA	CG9422 gene product from transcript CG9422-RC	1.978	0.006714827
1638158_at	CG4845-RA	phagocyte signaling impaired	1.971	2.08E-05
1629752_at	CG5525-RA	CG5525 gene product from transcript CG5525-RA	1.961	9.10E-05
1639335_at	GM06790	pumilio	1.941	0.001499321
1636948_a_at	CG6778-RA	Glycyl-tRNA synthetase	1.861	5.56E-04
1634739_a_at	CG4001-RA	Phosphofructokinase	1.839	0.008305934
1638956_at	CG3665-RC	Fasciclin 2	1.831	0.004900134
1630324_at	CG7989-RA	wicked	1.829	5.79E-04
1636071_a_at	CG7035-RA	cap binding protein 80	1.828	1.24E-05
1623193_at	CG33123-RA	CG33123 gene product from transcript CG33123-RA	1.819	0.001099777
1630408_s_at	CG7033-RC	CG7033 gene product from transcript CG7033-RA	1.811	0.002246746
1634081_at	CG6258-RA	Replication factor C 38kD subunit	1.809	0.001230874
1624180_at	CG2222-RA	CG2222 gene product from transcript CG2222-RB	1.799	0.005006902
1636612_a_at	CG11084-RA	prickle	1.796	0.001915067
1624419_a_at	CG8439-RA	T-complex Chaperonin 5	1.775	0.002378092
1632291_at	CG6625-RA	Soluble NSF attachment protein	1.762	0.005031917
1629026_at	CG3821-RA	Aspartyl-tRNA synthetase	1.760	8.69E-04

1625594_s_at	CG15862-RA	cAMP-dependent protein kinase R2	1.755	7.06E-04
1627492_at	CG5018-RA	lethal (3) 72Dn	1.751	0.005077979
1636274_at	CG6531-RA	wengen	1.745	0.009502802
1630638_at	CG1828-RA	CG1828 gene product from transcript CG1828-RB	1.740	0.00493557
1630141_at	CG2158-RA	Nucleoporin 50	1.714	0.001043711
1635047_s_at	CG18214-RA	CG18214 gene product from transcript CG18214-RA	1.711	6.08E-04
1639908_a_at	CG9705-RB	CG9705 gene product from transcript CG9705-RA	1.696	1.56E-04
1624247_at	CG5543-RA	CG5543 gene product from transcript CG5543-RA	1.681	5.66E-04
1639118_a_at	CG12749-RA	Heterogeneous nuclear ribonucleoprotein at 87F	1.678	0.01084942
1632418_s_at	CG9755-RA	pumilio	1.670	0.007546981
1624779_at	CG32253-RA	CG11583 gene product from transcript CG11583-RA	1.667	0.002915495
1624774_a_at	CG3665-RB	Fasciclin 2	1.663	0.009899339
1636468_a_at	CG5033-RA	CG5033 gene product from transcript CG5033-RA	1.658	0.026317228
1626845_at	CG8351-RA	CG8351 gene product from transcript CG8351-RA	1.656	3.33E-04
1637169_at	CG12202-RA	CG12202 gene product from transcript CG12202-RA	1.649	4.39E-04
1625856_at	CG10798-RA	diminutive	1.642	0.014672076
1628665_at	CG6841-RA	CG6841 gene product from transcript CG6841-RA	1.642	5.49E-04
1637217_at	CG5714-RA	ecdysoneless	1.641	2.17E-04
1632043_a_at	CG9198-RA	shattered	1.640	0.012816613
1627916_s_at	CG1799-RC	raspberry	1.609	0.006575399
1630178_at	CG12082-RA	CG12082 gene product from transcript CG12082-RB	1.607	0.005029804
1640595_at	CG12301-RA	CG12301 gene product from transcript CG12301-RA	1.583	0.029998414
1623383_at	CG7507-RA	Dynein heavy chain 64C	1.573	0.003554492
1638070_at	CG10418-RA	CG10418 gene product from transcript CG10418-RA	1.572	0.03060154
1639736_at	CG8326-RA	CG8326 gene product from transcript CG8326-RA /// --	1.571	8.31E-04
1639554_at	CG3333-RA	Nucleolar protein at 60B	1.562	0.0418354
1625887_at	CG10732-RA	CG10732 gene product from transcript CG10732-RA	1.562	0.035024453
1627349_at	CG4539-RA	Bekka	1.558	0.022578368
1624321_at	CG8556-RA	CG8556 gene product from transcript CG8556-RB	1.556	0.002188316
1631142_a_at	CG18026-RA	Calcium activated protein for secretion	1.544	0.041220438
1626266_at	CG10059-RA	CG10059 gene product from transcript CG10059-RA	1.539	6.59E-04
1625458_a_at	CG7935-RA	moleskin	1.534	0.0258634
1630344_at	CG6724-RA	CG6724 gene product from transcript CG6724-RB	1.533	0.006711513
1640145_at	CG1430-RA	by S6	1.531	0.005144843
1631223_at	CG2100-RA	CG2100 gene product from transcript CG2100-RB	1.531	0.009050993
1634028_s_at	CG6203-RA	CG6203 gene product from transcript CG6203-RC	1.529	0.04000817
1634243_a_at	CG4673-RB	Npl4 ortholog	1.528	4.61E-04
1633112_at	CG4322-RA	CG4322 gene product from transcript CG4322-RD	1.524	0.024643764
1636346_at	CG7839-RA	CG7839 gene product from transcript CG7839-RA	1.523	0.028056152
1639224_at	CG9273-RA	Replication protein A2	1.522	0.001744668
1629950_at	CG1785-RA	CG1785 gene product from transcript CG1785-RA	1.508	0.035447083
1627337_at	CG11555-RA	CG11555 gene product from transcript CG11555-RA	1.507	0.00842296
1636673_s_at	CG1856-RF	tramtrack	0.663	0.020230813
1628032_a_at	CG31092-RA	Lipophorin receptor 2	0.659	0.009983126
1629227_at	CG5055-RA	bazooka	0.654	0.035730846
1637926_s_at	CG14084-RA	CG14084 gene product from transcript CG14084-RB	0.654	9.85E-04
1629029_at	CG5160-RA	CG5160 gene product from transcript CG5160-RA	0.651	0.038203683
1630378_at	CG17657-RA	fritz	0.651	0.004134304
1630531_at	CG3421-RA	CG3421 gene product from transcript CG3421-RA	0.650	0.005021415
1631498_a_at	CG14307-RC	fruitless	0.649	0.011007547
1632207_a_at	CG1212-RA	CG1212 gene product from transcript CG1212-RD	0.640	0.017192865
1634962_s_at	CG9325-RB	hu li tai shao	0.638	6.58E-05
1641336_at	CG6443-RA	--- /// CG6443 gene product from transcript CG6443-RA	0.638	0.014773252
1633089_a_at	CG12052-RJ	longitudinals lacking	0.636	9.38E-04
1627384_at	CG6225-RA	CG6225 gene product from transcript CG6225-RB	0.635	0.001568175
1636974_at	CG32146-RA	dally-like	0.634	0.036913905
1635427_a_at	CG31043-RA	GUK-holder	0.634	7.51E-04
1630942_at	CG5742-RA	CG5742 gene product from transcript CG5742-RB	0.632	0.001485239
1637917_s_at	CG1856-RE	tramtrack	0.631	0.01071164
1623411_at	CG12052-RN	longitudinals lacking	0.628	0.003327923
1639410_at	CG7013-RA	Mesencephalic astrocyte-derived neurotrophic factor	0.621	0.004924618
1628849_at	HDC07332	CG8201 gene product from transcript CG8201-RV	0.621	0.00188239
1635754_at	CG11987-RA	tango	0.615	1.46E-04
1627041_s_at	CG15444-RB	inebriated	0.614	0.037149344
1625279_a_at	CG10619-RA	tailup	0.612	0.03589902
1631242_at	CG4029-RA	jumeau	0.610	0.016876327
1638466_a_at	CG4370-RA	Inwardly rectifying potassium channel 2	0.606	0.01349143
1631340_at	CG8261-RA	G protein gamma 1	0.604	0.01811019
1631408_at	CG18024-RA	SoxNeuro	0.594	6.84E-04
1628336_a_at	CG12369-RB	Lachesin	0.594	0.008119137
1639139_a_at	CG8874-RB	Fps oncogene analog	0.593	0.002128981
1631207_at	CG4220-RA	elbow B	0.590	0.00514124
1630456_at	CG32156-RE	Myosin binding subunit	0.590	0.001187458
1626352_at	GM04742	scribbler	0.587	0.004717244
1625048_at	CG8318-RA	Neurofibromin 1	0.578	0.030962145
1631527_at	CG5642-RA	CG5642 gene product from transcript CG5642-RA	0.573	7.52E-04
1641448_a_at	CG18250-RA	Dystroglycan	0.562	0.004142304
1641068_a_at	CG6134-RA	spatzle	0.545	0.023638569
1637703_a_at	CG15154-RA	Suppressor of cytokine signaling at 36E	0.542	1.35E-05
1625542_a_at	CG4898-RE	Tropomyosin 1	0.536	0.001913851
1635737_at	CG6701-RA	CG6701 gene product from transcript CG6701-RD	0.527	0.001728137
1632508_s_at	CG11282-RA	capricious	0.525	0.008637066
1626165_at	CG3754-RA	Phosphodiesterase 9	0.524	0.002821438
1626492_s_at	CG8978-RB	Actin-related protein 2/3 complex, subunit 1	0.517	0.002901172
1630841_at	CG8668-RA	CG8668 gene product from transcript CG8668-RA	0.509	0.002753527
1633384_at	CG9250-RA	M-phase phosphoprotein 6	0.509	1.93E-04

1623877_a_at	CG4220-RA	elbow B	0.498	0.023710683
1638580_at	S.C3L001717	capricious	0.479	0.020279841
1641365_s_at	SD27140	CG11352 gene product from transcript CG11352-RC	0.464	1.94E-05
1628604_at	CG5893-RA	Dichaete	0.458	0.005151275
1624533_s_at	CG8651-RA	trithorax	0.427	2.59E-04
1640867_s_at	CG7223-RB	heartless	0.395	0.004595591
1634920_at	LD07388	capricious	0.385	0.008747041
1638807_s_at	CG4829-RB	CG4829 gene product from transcript CG4829-RB	0.358	1.22E-04
1625276_a_at	CG7266-RC	Ecdysone-induced protein 28/29kD	0.312	0.00480965
1635725_a_at	CG31332-RC	CG31352 gene product from transcript CG31352-RA ///	0.216	9.19E-04
1629713_at	CG9187-RA	CG9187 gene product from transcript CG9187-RA	0.211	6.37E-06
**Reproduction**				
1640377_s_at	CG2849-RB	Ras-related protein	27.693	9.37E-05
1626984_at	CG1152-RA	Glucose dehydrogenase	15.261	1.17E-05
1638869_at	CG10112-RA	Cuticular protein 51A	8.771	5.60E-04
1623540_at	CG31704-RA	CG31704 gene product from transcript CG31704-RB	7.751	1.24E-06
1630593_at	CG33126-RA	Neural Lazarillo	3.415	0.019701043
1626392_s_at	X01472	Myocyte enhancer factor 2	3.292	2.38E-04
1636189_at	CG12306-RA	CG12306 gene product from transcript CG12306-RA	3.172	0.002657874
1630729_at	CG6535-RA	telomere fusion	2.788	1.16E-04
1626425_at	CG4173-RA	Septin 2	2.331	3.96E-04
1630619_at	CG9456-RA	Serpin 42Dd	2.287	0.009781473
1640235_at	CG5123-RA	Wrinkled	2.214	6.14E-04
1628188_at	S.C2R002789	Nucleolar protein at 60B	2.207	0.011176612
1630772_at	CG5557-RA	squeeze	2.198	0.00629058
1639162_at	CG6072-RA	sarah	2.174	0.010623534
1631493_at	CG1548-RA	CG1548 gene product from transcript CG1548-RA	2.152	0.030961603
1624686_a_at	CG32498-RO	dunce	2.086	0.004152716
1624663_a_at	CG8821-RA	vismay	2.063	0.004561812
1641267_at	CG18572-RA	rudimentary	2.048	9.03E-05
1638158_at	CG4845-RA	phagocyte signaling impaired	1.971	2.08E-05
1639335_at	GM06790	pumilio	1.941	0.001499321
1634219_a_at	CG1633-RB	thioredoxin peroxidase 1	1.935	0.005328337
1633951_at	CG7769-RA	piccolo	1.923	3.51E-04
1629229_a_at	CG3992-RA	serpent	1.922	0.024780674
1640835_a_at	CG5320-RA	Glutamate dehydrogenase	1.863	0.003665939
1624807_at	CG5629-RB	Phosphopantothenoylcysteine synthetase	1.859	0.010597768
1630324_at	CG7989-RA	wicked	1.829	5.79E-04
1637398_a_at	CG3399-RC	cappuccino	1.826	0.019995738
1625724_at	CG14940-RA	CG44007 gene product from transcript CG44007-RG	1.814	0.003336879
1625107_at	CG4934-RA	brainiac	1.813	0.003205006
1635021_at	CG2019-RA	dispatched	1.779	0.02253891
1625038_s_at	CG10895-RA	loki	1.776	2.84E-04

1626170_at	CG18445-RA	oysgedart	1.743	0.004417544
1634586_at	CG9614-RJ	pipe	1.735	0.004968485
1638011_a_at	CG6148-RA	Putative Achaete Scute Target 1	1.671	4.41E-04
1632418_s_at	CG9755-RA	pumilio	1.670	0.007546981
1634636_at	CG6426-RA	CG6426 gene product from transcript CG6426-RA	1.644	0.010333432
1625856_at	CG10798-RA	diminutive	1.642	0.014672076
1637217_at	CG5714-RA	ecdysoneless	1.641	2.17E-04
1640496_at	CG5722-RA	Niemann-Pick type C-1a	1.631	0.001503809
1633798_at	CG5654-RA	ypsilon schachtel	1.622	6.15E-04
1633711_a_at	CG31012-RA	CIN85 and CD2AP orthologue	1.612	0.00773198
1634695_at	CG2684-RA	lodestar	1.609	0.004681077
1627916_s_at	CG1799-RC	raspberry	1.609	0.006575399
1639259_at	CG9006-RA	Enigma	1.606	0.0021889
1631993_s_at	CG6058-RB	Aldolase	1.599	0.006915187
1623164_a_at	CG8127-RB	Ecdysone-induced protein 75B	1.591	0.044770837
1631973_at	CG5581-RA	Otefin	1.591	0.024740806
1623383_at	CG7507-RA	Dynein heavy chain 64C	1.573	0.003554492
1635393_s_at	GM02640	Ecdysone-induced protein 75B	1.572	0.001215019
1639554_at	CG3333-RA	Nucleolar protein at 60B	1.562	0.0418354
1632251_s_at	CG6181-RA	CG6181 gene product from transcript CG6181-RA	1.556	1.06E-04
1624321_at	CG8556-RA	CG8556 gene product from transcript CG8556-RB	1.556	0.002188316
1628213_s_at	CG5887-RA	CG5887 gene product from transcript CG5887-RA	1.533	0.032448683
1634028_s_at	CG6203-RA	CG6203 gene product from transcript CG6203-RC	1.529	0.04000817
1628435_at	CG16901-RA	squid	1.529	0.001872392
1639205_s_at	CG10128-RC	transformer 2	1.523	0.001660005
1636809_at	CG6944-RA	Lamin	1.515	0.027962605
1638464_a_at	CG14039-RA	quick-to-court	1.512	0.006902332
1636919_at	CG9401-RA	mago nashi	1.510	0.017198913
1631183_at	CG9366-RA	rho-like	1.504	9.78E-04
1638887_a_at	CG6339-RA	CG6339 gene product from transcript CG6339-RE	1.503	0.004764688
1636673_s_at	CG1856-RF	tramtrack	0.663	0.020230813
1640239_at	CG30149-RB	rigor mortis	0.660	1.46E-04
1632648_at	CG32922-RA	CG43161 gene product from transcript CG43161-RD	0.657	0.03288023
1629227_at	CG5055-RA	bazooka	0.654	0.035730846
1631498_a_at	CG14307-RC	fruitless	0.649	0.011007547
1632185_s_at	CG5207-RA	SCP-containing protein A /// SCP-containing protein B /	0.644	0.016405914
1629700_at	S.CX001531	maternal gene required for meiosis	0.640	0.001959656
1634562_s_at	CG13503-RB	Verprolin 1	0.640	0.00728029
1634962_s_at	CG9325-RB	hu li tai shao	0.638	6.58E-05
1633089_a_at	CG12052-RJ	longitudinals lacking	0.636	9.38E-04
1628921_s_at	SD11801	modifier of mdg4 /// CG42769 gene product from transc	0.635	0.004457435
1628562_s_at	CG32491-RT	modifier of mdg4 /// CG42769 gene product from transc	0.632	2.57E-04
1637917_s_at	CG1856-RE	tramtrack	0.631	0.01071164

1623411_at	CG12052-RN	longitudinals lacking	0.628	0.003327923
1629270_at	CG17958-RA	Serendipity delta	0.628	0.010914986
1639083_at	CG1945-RC	fat facets	0.625	0.009429477
1628849_at	HDC07332	CG8201 gene product from transcript CG8201-RV	0.621	0.00188239
1635754_at	CG11987-RA	tango	0.615	1.46E-04
1625283_at	CG32491-RM	modifier of mdg4	0.607	0.010529318
1627605_at	CG1071-RA	E2F transcription factor 2	0.605	2.71E-04
1630456_at	CG32156-RE	Myosin binding subunit	0.590	0.001187458
1626158_s_at	CG9655-RA	nessy	0.578	0.001264002
1639308_at	CG32491-RF	modifier of mdg4	0.565	0.001798739
1641448_a_at	CG18250-RA	Dystroglycan	0.562	0.004142304
1632711_at	CG7864-RA	Peroxin 10	0.562	0.012550153
1634063_a_at	CG31317-RA	CG31317 gene product from transcript CG31317-RC	0.559	0.04519155
1641068_a_at	CG6134-RA	spatzle	0.545	0.023638569
1637703_a_at	CG15154-RA	Suppressor of cytokine signaling at 36E	0.542	1.35E-05
1631831_at	CG31040-RA	CG31040 gene product from transcript CG31040-RA	0.537	4.07E-04
1631742_s_at	CG32491-RQ	modifier of mdg4	0.537	0.00332743
1625542_a_at	CG4898-RE	Tropomyosin 1	0.536	0.001913851
1638875_at	CG6542-RA	Egg-derived tyrosine phosphatase	0.525	0.008692758
1633152_at	CG32491-RA	modifier of mdg4	0.523	0.005434811
1629129_at	CG7525-RA	Tie-like receptor tyrosine kinase	0.519	3.18E-04
1626492_s_at	CG8978-RB	Actin-related protein 2/3 complex, subunit 1	0.517	0.002901172
1635360_at	CG10830-RA	CG10830 gene product from transcript CG10830-RA	0.516	0.002748158
1638041_at	CG32491-RH	modifier of mdg4	0.516	0.012785054
1636765_at	CG32491-RJ	modifier of mdg4	0.516	0.005565568
1632381_at	CG14226-RA	domeless	0.506	0.026396735
1626461_at	CG4357-RA	Heat shock protein 60 related /// sodium chloride cotran	0.477	0.001494621
1636606_at	CG7623-RA	slalom	0.470	0.007295398
1637478_s_at	CG31000-RB	hephaestus	0.448	0.015236953
1641021_a_at	CG6297-RB	CG6297 gene product from transcript CG6297-RC	0.442	4.49E-06
1627953_at	CG32491-RI	modifier of mdg4	0.440	0.004287487
1626624_s_at	CG31794-RC	Paxillin	0.437	0.001420985
1624533_s_at	CG8651-RA	trithorax	0.427	2.59E-04
1638821_a_at	CG31732-RA	yuri gagarin	0.419	0.001551989
1634052_s_at	CG32491-RA	modifier of mdg4	0.408	0.00108066
1639568_at	CG31285-RA	liquid facets-Related	0.407	7.79E-05
1640867_s_at	CG7223-RB	heartless	0.395	0.004595591
1622976_at	CG32491-RB	modifier of mdg4	0.389	0.00638984
1627483_at	CT35609	modifier of mdg4	0.375	0.003102537
1628458_at	CG13777-RC	milton	0.324	0.004074056
1631500_at	CG32491-RR	modifier of mdg4	0.308	1.91E-04
1623807_a_at	CG13777-RA	milton	0.294	7.27E-04
1632404_at	CG5760-RA	tetracycline resistance	0.246	9.47E-05

1632715_at	RH40482	Tie-like receptor tyrosine kinase	0.220	2.80E-04
1625153_at	CG33105-RA	p24-related-2 /// CG31352 gene product from transcript	0.023	0.0014128
**Response to oxidative stress**				
1623256_at	CG5164-RA	Glutathione S transferase E1	9.331	0.001191725
1630487_s_at	CG32041-RA	Heat shock protein 22 /// Heat shock gene 67Bb	9.304	2.21E-05
1630593_at	CG33126-RA	Neural Lazarillo	3.415	0.019701043
1633471_at	CG11765-RA	Peroxiredoxin 2540-2	2.934	0.017057572
1639480_at	CG6871-RA	Catalase	2.592	0.009853024
1633391_at	CG8913-RA	Immune-regulated catalase	2.184	0.04989941
1639526_a_at	CG7717-RA	CG7717 gene product from transcript CG7717-RB	2.109	0.003677665
1638844_s_at	CG3714-RC	CG3714 gene product from transcript CG3714-RG	2.107	4.67E-04
1634219_a_at	CG1633-RB	thioredoxin peroxidase 1	1.935	0.005328337
1628352_a_at	CG8798-RB	Lon protease	1.669	0.002491571
1628257_s_at	CG12013-RB	CG12013 gene product from transcript CG12013-RC	1.636	0.016961023
1630941_s_at	CG16944-RA	stress-sensitive B	1.613	0.001486399
1639507_at	CG6666-RA	Succinate dehydrogenase C	1.538	9.02E-04
1636974_at	CG32146-RA	dally-like	0.634	0.036913905
1625048_at	CG8318-RA	Neurofibromin 1	0.578	0.030962145
1633290_a_at	CG4482-RA	moladietz	0.456	0.00841207
1640097_at	CG12002-RA	Peroxidasin	0.437	0.03795444
1625276_a_at	CG7266-RC	Ecdysone-induced protein 28/29kD	0.312	0.00480965

Supplementary Table 2. A list of primers.

Gene	Primer	Primer sequence (5' --> 3')	References
**eccDNA assay**			
rp49	RP49-F	gtg cga gtg ccg agt tca at	[14]
	RP49-R	tgg tgc tgc tat ccc aat ctc	
28S repeats	28S-F	tag cgg gtg ttg aca caa tg	[14]
	28S-R	acg agg cat ttg gct att tc	
Stellate repeates	stellate -F	gat acg ttc aac cag atg ggc t	[14]
	stellate -R	gca tat att ttc ggt gca tag caa t	
**QPCR analysis-1**			
CG14416	QT00497371		purchased from Qiagen
CG12498	QT00497364		purchased from Qiagen
white gene	QT00497357		purchased from Qiagen
CG32795	QT00497350		purchased from Qiagen
alpha-tubulin	a-Tub-F	aac ctg aac cgt ctg att gg	This study
	a-Tub-R	ggt cac cag agg gaa gtg aa	
Pre-rRNA	Pre-rRNA-F	ggc cgt att cga atg gat tta	[15]
	Pre-rRNA-R	cta ctg gca gga tca acc aga	
actin 5C	actin5C-F	aag ttg ctg ctc tgg ttg tcg	[16]
	actin5C-R	gcc aca cgc agc tca ttg tag	
rp49	rp49-F	aga tcg tga aga agc gca cca ag	[16]
	rp49-R	cac cag gaa ctt ctt gaa tcc gg	
su(var)2-5	Su(var)2-5-F	cgc aag gat gag gag aag tca	[16]
	Su(var)2-5-R	tcc tga aac ggg aat ggt gtc	
su(var)3-9	Su(var)3-9-F1	ctg gag gca ttg cca cta at	This study
	Su(var)3-9-R1	agg gct cgt tct cca att tt	
**QPCR analysis for transposons**			
Accord	Accord2-F	ttt cat tca aac aat tcc ctt acc t	[14]
	Accord2-R	agt tat gta gtc cat ttt ccc ggt t	
Frogger	Frogger-F	gaa ata cag atc ctc acc acg aag a	[14]
	Frogger-R	agg aat gca tat cct gaa tac gac t	
Ivk	Ivk-F	ata tca aaa cct aac aaa ccc cct t	[14]
	Ivk-R	cgt cca tcg tgc tat gat ttc tt	
G3	G3-F	acc ata cca gtt agt gaa acg gtg a	[14]
	G3-R	ctg ctt tat aca gcc gta att tgt tc	
R2	R2-F	ggg tga ccc ttt gtc tcc tat tct a	[14]
	R2-R	caa cgt ctt gtc caa caa tac ttg a	
Het-A	HeT-A-F	cgc gcg gaa ccc atc ttc aga	[17]
	HeT-A-R	cgc cgc agt cgt ttg gtg agt	
Mdg1	Mdg1-F	ctt cag tac cca gat ttc agc aaa	[17]
	Mdg1-R	ccg ctc cac atg ctt gtt ta	
I-element	I-element-F	tcg act tcg gac tgt cta ctc tat gt	[17]
	I-element-R	gtg acg agg gag gtg tgc at	
Roo	Roo-F	gtc tga ggc atc cgt ttg gt	[17]

	Roo-R	cgt gtg gtg agg ttt acg aca a	
R1	R1Dm-F	tgc gcc tac ggt gag gtt	[17]
	R1Dm-R	ccg aca gct acg cac ata cg	
Pogo	Pogo-F	cca aaa att taa cga cgc ctt t	[17]
	Pogo-R	gcg cca gcg cca aa	
Hobo	Hobo-F	caa gtg cga ccg tcg aca t	[17]
	Hobo-R	aag tga tgc cca aaa agt ttc ttt	
Copia	Copia-F	gag gtt gtg cct cca ctt aag att	[17]
	Copia-R	caa tac cac gct tag tgg cat aaa	
NOF	NOF-F	tgc atc gaa gct gtt tgc a	[17]
	NOF-R	gag ttt taa cgg tct tgc gtt tc	
Blastopia	Flea-F	ttg tag ctg gcc gca aga t	[17]
	Flea-R	ttg acc cct gtg act tcg tat g	
Juan	Juan-F	gac cgt ttt aat ata ccc gaa tac act a	[17]
	Juan-R	cgc cgc ctc ccc tac t	
ZAM	ZAM_fwd	act tga cct gga tac act cac aac	[18]
	ZAM_rev	gag tat tac ggc gac tag gga tac	
412	412_fwd	aaa gta cgg tcc aat gaa gac g	[18]
	412_rev	gtg gtg atg agc tgt tga tgt t	
Gypsy	Gypsy_fwd	caa caa tct gaa ccc acc aat ct	[18]
	Gypsy_rev	tat gaa cat cat gag ggt gaa cg	
297	297-F	ctg gca aag gga ttt cat ca	[19]
	297-R	tgc att cct aag gcc aaa tg	
1731	1731-F	tat ggg ctg agg cga taa ac	[19]
	1731-R	caa gtg gct cac tgc tgg ta	
blood	blood-F	tat cgc atg gca gat agc caa a	[19]
	blood-R	cgt gga att cgg aag tgg ttt c	
Invader4	INVDR_fwd2	gat tgg cca aga cat att aga gct tgg	[20]
	INVDR_rev2	gca tgt ttt cct tgc ttg tac gct c	
TART	TART_5′ UTR_F	gat aat agc cga taa gcc cgc ca	[21]
	TART_5′ UTR_R	aag aca cag cgg ttg atc gat atg	
**snoRNAs**			
CR33780 (snoRNA:660)	CR33780-F1	ccc ggt cta ctc acc tgt ct	This study
	CR33780-R2	cac aca ccc aga acc acc ac	
CR34591 (snoRNA:Ψ28S-1060)	CR34591-F2	cag cat ctc gct ttt gca t	This study
	CR34591-R1	ttt aga aga ggg cgt cca ga	
CR33637 (snoRNA:Or-aca5)	CR33637-F1	gca tga gtc gct ggt gta aa	This study
	CR33637-R1	ctg cga gca agt aaa aag ca	

CR34611 (snoRNA:Ψ28S-3571)	CR34611-F1	gac tca atg atc cag ttg tgc	This study
	CR34611-R1	tgc gaa ttg ggt aaa act cc	
CR34633 (snoRNA:Ψ18S-1086)	CR34633-F3	tcc gca cga agt atc tcc ag	This study
	CR34633-R1	gga aag tcg tgt cgc cta ac	
CR34546 (snoRNA:Ψ28S-2444)	CR34546-F1	gaa tgt gcg cat agc aaa ga	This study
	CR34546-R1	cac gca tgg aat gta gca ag	
CR33754 (snoRNA:Ψ28S-291)	CR33754-F1	gcc tgg cct aaa aag cat tc	This study
	CR33754-R1	atc cct aaa ccg tgc att ga	
CR32874 (snoRNA:Me18S-G1620)	CR32874-F1	tct ctg atg atc ttt tac aca gac ct	This study
	CR32874-R1	ttt tct gca cgg tta tga gc	
CR34703 (snoRNA:Me18S-A1806)	CR34703-F1	aac ttt act acc cga acc atc g	This study
	CR34703-R1	cag aat tac gtc cct tta cca gag	
CR34562 (snoRNA:Me28S-G1083a)	CR34562-F1	agc aat aac ttt cgc ccc ta	This study
	CR34562-R1	tcg cat tca aca tga gaa gta tc	
CR34565 (snoRNA:Me28S-G1083d)	CR34565-F1	gag ctt taa ctt tcg ccc cta	This study
	CR34565-R1	caa tct cgc att caa cag ga	
CR34563 (snoRNA:Me28S-G1083b)	CR34563-F1	gca tta act ttc gcc cct atc	This study
	CR34563-R1	acc aat ctc gca ttc aac aa	
CR34695 (snoRNA:Me5.8S-G74)	CR34695-F1	tgc tct gat gac aaa aac acg	This study
	CR34695-R3	agg aca cat agt gtc tca aca tga	
CR34537 (snoRNA:Me18S-A1061)	CR34537-F1	cta tta act ttc gtt cta ctg agt ggt	This study
	CR34537-R1	atc aga aaa ata tat ccg cat tta cc	
CR32807 (snoRNA:M)	CR32807-F1	tga ttt caa ctt cac tgc tga c	This study
	CR32807-R1	tat cag aga ctg ggg gca aa	
CR34684 (snoRNA:Me18S-C1096)	CR34684-F1	aga aca cat atg att aac cgt gat t	This study
	CR34684-R1	ttg gtc agt ccg ccc tag t	
CR33674 (snoRNA:Me18S-C1831)	CR33674-F1	atg ggc gga cag aat gct ac	This study
	CR33674-R1	ggc tgt cct cta cac ggt ga	
CR34558 (snoRNA:Ψ28S-1175a)	CR34558-F1	tgt ccc ttt tcc ttc gaa tc	This study
	CR34558-R1	tct gaa gca tca ccc aat ct	
CR34559 (snoRNA:Ψ28S-1175b)	CR34559-F1	aac cct ttt cct tcg aat cat	This study
	CR34559-R1	tca ccc aat ttg cac aat aaa	
CR34560 (snoRNA:Ψ28S-1175c)	CR34560-F1	tcc ctt ttc ctc cga atc at	This study
	CR34560-R1	cct gaa gca tca ccc aat tt	
**PCR templates for dsRNA production**			
dTCTP dsRNA	Forward	taa tac gac tca cta tag g (T7 promoter) atg aag atc tac aag gat atc	[13]

	Reverse	taa tac gac tca cta tag g (T7 promoter) acc ggt acg cgt aga atc gag	[13]
pSK(-) dsRNA	Forward	taa tac gac tca cta tag g (T7 promoter) atc gat aag ctt gat atc gaa ttc	[13]
	Reverse	taa tac gac tca cta tag g (T7 promoter) gca ccg cct aca tac ctc gct ctg	[13]
**QPCR analysis-2**			
CG9116	QT00955780		purchased from Qiagen
CG11893	QT00982534		
CG2010	QT00985068		
CG4105	QT00933226		
CG18473	QT00971516		
CG7884	QT00925211		
CG8084	QT00942536		
CG9021	QT00930461		
CG13207	QT00502187		
CG10131	QT00946834		
CG11836	QT00982233		
CG7142	QT00977354		
CG32356	QT00502383		
CG8277	QT00960141		
CG15671	QT01100638		
CG4680	QT00510041		
CG13722	QT00958230		
CG1374	QT00939960		
CG4393	QT00980714		
CG8805	QT00988498		
CG9908	QT00923153		
CG14109	QT00963907		
CG42365	QT00951818		
CG9632	QT00975765		
CG15905	QT00950579		
CG43740	QT00919506		
CG11345	QT00958188		
CG17835	QT00944447		
CG42334	QT00964992		
CG5955	QT00966350		
CG4881	QT00934829		
CG1738	QT00920143		
CG5873	QT00976822		
CG30457	QT00948675		
CG13813	QT00966063		
CG8785	QT00945399		
CG12858	QT00946904		
CG6864	QT00976031		

CG13689	QT00927136
CG2137	QT00941255
CG32239	QT00958349
CG11897	QT00984942
**Primers were designed by the Primer3 program (http://bioinfo.ut.ee/primer3-0.4.0/) under default settings but with minor modifications such as Drosophila mispriming library and 50-100 product size.**	

Supplementary Discussion

It has been reported that mammalian ATM inhibits both local Pol-II near the sites of induced DSBs and pan-nuclear Pol-I activities responding to induced DSBs[10, 11]. Because loss of Tctp impairs ATM activity for DNA repair [13, 22], it is possible that the increased Pol-I and Pol-II activities in *Tctp* mutants could be indirect effects of reduced ATM function. Western analysis of larval SG tissues showed that Pol-II activity is not increased in $atm^{6/6}$ mutant (strong hypomorph), compared with the wild-type level (Supplementary Fig.9a). Hence, Pol-II activity increase in *Tctp* mutants is unlikely due to reduced ATM function at least in developing SG. In contrast to Pol-II, Pol-I activity in atm^{6} was modestly increased but to a weaker level than that in *Tctp* mutants (Supplementary Fig.9a, arrow). Thus, a reduction of ATM activity in *Tctp* mutants may contribute to an increase of Pol-I activity but to a limited extent, if any.

In mammals, both hBrm and Brg-1, together with ATM-kinase, are required to repair IR-induced DSBs at γ-H2AX foci[23]. Since TCTP interacts with ATM to repair IR-induced DSBs, we tested whether Tctp and ATM might work with Brm in a protein complex formed at γ-H2Av foci (mammalian H2AX homolog in flies) for DNA repair. In nuclei of wild-type SG cells, γ-H2Av foci were formed at the chromocenter region and multiple places in chromosome arms, while most Brm proteins did not overlap with γ-H2Av foci (Supplementary Fig.10a). In $Tctp^{EY/h59}$, γ-H2Av staining was considerably reduced, but the overall Brm staining pattern seemed to be intact (Supplementary Fig.10b). We also generated flp-out clones of *Tctp*-RNAi or Brm^{DN} in SG. While *Tctp*-RNAi caused γ-H2Av reduction, Brm^{DN} clones showed similar γ-H2Av pattern as adjacent wild-type cells (Supplementary Fig.10d-e). These results suggest that ATM functions together with Tctp for DNA repair but not with Brm in developing organs of *Drosophila*.

In mammals, chromatin remodelers like Brahma-associated factors (BAF/PBAF) have various biological functions ranging from growth control to differentiation during normal development, such as early embryogenesis, neurogenesis, muscle differentiation and heart-tube formations [24, 25]. In addition, mouse embryonic stem cells (ESCs) require BAF/PBAF to maintain their self-renewal and pluripotent states such as hyperdynamic (open) chromatin and Nanog or Oct4 expression [24]. Mis-regulation of BAF/PBAF can result in various developmental defects and cancers such as malignant rhabdoid tumors (MRT), lung, colorectal and skin cancers [25, 26, 27]. Of BAF/PBAF subunits, SNF5/INI1/SMARCB is well-known for its regulatory function on BAF/PBAF [25, 26]. SNF5 mutation causes MRT, which can be ameliorated by Brg-1 (human homolog of *Drosophila* Brm) depletion in human MRT cell lines. As in mammals, *Drosophila* Brm remodeler requires SNR1 (human SNF5 homologue) as a negative regulatory subunit. *snr1* mutant flies show multiple phenotypes such as small eye, short life span, extra wing veins, suppressed PEV eye color and increased transcriptional elongation activity [28]. Interestingly, our data show that *Tctp* mutants have remarkably similar phenotypes with *snr1* mutants, some of which are improved by *brm* heterozygous mutation. Thus, our results suggest that Tctp may function as an additional inhibitory subunit to refine the Brm remodeler function. It will be interesting to see whether human TCTP has a similar inhibitory function to human Brm homologues (Brg-1 or BRM). If the interaction between Tctp and Brm is evolutionarily conserved in mammalian systems, our findings might provide valuable insights into finding the new regulatory mechanism for the human SWI/SNF remodeler family and new clues for cancer treatment.

Supplementary References

1. Cans C*, et al.* Translationally controlled tumor protein acts as a guanine nucleotide dissociation inhibitor on the translation elongation factor eEF1A. *Proc Natl Acad Sci U S A* **100**, 13892-13897 (2003).

2. Yoon T, Kim M, Lee K. Inhibition of Na,K-ATPase-suppressive activity of translationally controlled tumor protein by sorting nexin 6. *FEBS Lett* **580**, 3558-3564 (2006).

3. Amson R, Pece S, Marine JC, Di Fiore PP, Telerman A. TPT1/ TCTP-regulated pathways in phenotypic reprogramming. *Trends Cell Biol* **23**, 37-46 (2013).

4. Kal AJ, Mahmoudi T, Zak NB, Verrijzer CP. The Drosophila brahma complex is an essential coactivator for the trithorax group protein zeste. *Genes Dev* **14**, 1058-1071 (2000).

5. Moshkin YM, Mohrmann L, van Ijcken WF, Verrijzer CP. Functional differentiation of SWI/SNF remodelers in transcription and cell cycle control. *Mol Cell Biol* **27**, 651-661 (2007).

6. Vorobyeva NE, Nikolenko JV, Nabirochkina EN, Krasnov AN, Shidlovskii YV, Georgieva SG. SAYP and Brahma are important for 'repressive' and 'transient' Pol II pausing. *Nucleic Acids Res* **40**, 7319-7331 (2012).

7. Collins RT, Furukawa T, Tanese N, Treisman JE. Osa associates with the Brahma chromatin remodeling complex and promotes the activation of some target genes. *The EMBO journal* **18**, 7029-7040 (1999).

8. Armstrong JA*, et al.* The Drosophila BRM complex facilitates global transcription by RNA polymerase II. *The EMBO journal* **21**, 5245-5254 (2002).

9. Muller HJ, Altenburg E. The Frequency of Translocations Produced by X-Rays in Drosophila. *Genetics* **15**, 283-311 (1930).

10. Larsen DH*, et al.* The NBS1-Treacle complex controls ribosomal RNA transcription in response to DNA damage. *Nat Cell Biol* **16**, 792-803 (2014).

11. Shanbhag NM, Rafalska-Metcalf IU, Balane-Bolivar C, Janicki SM, Greenberg RA. ATM-dependent chromatin changes silence transcription in cis to DNA double-strand breaks. *Cell* **141**, 970-981 (2010).

12. Mehrotra S, Maqbool SB, Kolpakas A, Murnen K, Calvi BR. Endocycling cells do not apoptose in response to DNA rereplication genotoxic stress. *Genes Dev* **22**, 3158-3171 (2008).

13. Hong ST, Choi KW. TCTP directly regulates ATM activity to control genome stability and organ development in Drosophila melanogaster. *Nat Commun* **4**, 2986 (2013).

14. Vujatovic O, Zaragoza K, Vaquero A, Reina O, Bernues J, Azorin F. Drosophila melanogaster linker histone dH1 is required for transposon silencing and to preserve genome integrity. *Nucleic Acids Res* **40**, 5402-5414 (2012).

15. Larson K*, et al.* Heterochromatin formation promotes longevity and represses ribosomal RNA synthesis. *PLoS Genet* **8**, e1002473 (2012).

16. Lu X*, et al.* Linker histone H1 is essential for Drosophila development, the establishment of pericentric heterochromatin, and a normal polytene chromosome structure. *Genes Dev* **23**, 452-465 (2009).

17. Tan H, Qurashi A, Poidevin M, Nelson DL, Li H, Jin P. Retrotransposon activation contributes to fragile X premutation rCGG-mediated neurodegeneration. *Hum Mol Genet* **21**, 57-65 (2012).

18. Sienski G, Donertas D, Brennecke J. Transcriptional silencing of transposons by Piwi and maelstrom and its impact on chromatin state and gene expression. *Cell* **151**, 964-980 (2012).

19. Ohtani H, Iwasaki YW, Shibuya A, Siomi H, Siomi MC, Saito K. DmGTSF1 is necessary for Piwi-piRISC-mediated transcriptional transposon silencing in the Drosophila ovary. *Genes Dev* **27**, 1656-1661 (2013).

20. Phalke S, Nickel O, Walluscheck D, Hortig F, Onorati MC, Reuter G. Retrotransposon silencing and telomere integrity in somatic cells of Drosophila depends on the cytosine-5 methyltransferase DNMT2. *Nat Genet* **41**, 696-702 (2009).

21. Silva-Sousa R, Varela MD, Casacuberta E. The Putzig partners DREF, TRF2 and KEN are involved in the regulation of the Drosophila telomere retrotransposons, HeT-A and TART. *Mob DNA* **4**, 18 (2013).

22. Zhang J*, et al.* Role of the translationally controlled tumor protein in DNA damage sensing and repair. *Proc Natl Acad Sci U S A* **109**, E926-933 (2012).

23. Park JH*, et al.* Mammalian SWI/SNF complexes facilitate DNA double-strand break repair by promoting gamma-H2AX induction. *The EMBO journal* **25**, 3986-3997 (2006).

24. Ho L, Crabtree GR. Chromatin remodelling during development. *Nature* **463**, 474-484 (2010).

25. Yaniv M. Chromatin remodeling: from transcription to cancer. *Cancer Genet* **207**, 352-357 (2014).

26. Hohmann AF, Vakoc CR. A rationale to target the SWI/SNF complex for cancer therapy. *Trends Genet* **30**, 356-363 (2014).

27. Shain AH, Pollack JR. The spectrum of SWI/SNF mutations, ubiquitous in human cancers. *PLoS One* **8**, e55119 (2013).

28. Zraly CB, Dingwall AK. The chromatin remodeling and mRNA splicing functions of the Brahma (SWI/SNF) complex are mediated by the SNR1/SNF5 regulatory subunit. *Nucleic Acids Res* **40**, 5975-5987 (2012).
